# Evolvix BEST Names for semantic reproducibility across code2brain interfaces

**DOI:** 10.1111/nyas.13192

**Published:** 2016-12-05

**Authors:** Laurence Loewe, Katherine S. Scheuer, Seth A. Keel, Vaibhav Vyas, Ben Liblit, Bret Hanlon, Michael C. Ferris, John Yin, Inês Dutra, Anthony Pietsch, Christine G. Javid, Cecilia L. Moog, Jocelyn Meyer, Jerdon Dresel, Brian McLoone, Sonya Loberger, Arezoo Movaghar, Morgaine Gilchrist‐Scott, Yazeed Sabri, Dave Sescleifer, Ivan Pereda‐Zorrilla, Andrew Zietlow, Rodrigo Smith, Samantha Pietenpol, Jacob Goldfinger, Sarah L. Atzen, Erika Freiberg, Noah P. Waters, Claire Nusbaum, Erik Nolan, Alyssa Hotz, Richard M. Kliman, Ayalew Mentewab, Nathan Fregien, Martha Loewe

**Affiliations:** ^1^ Wisconsin Institute for Discovery; ^2^ Laboratory of Genetics; ^3^ Departments of Computer Sciences; ^4^ Statistics; ^5^ Chemical and Biological EngineeringUniversity of Wisconsin‐MadisonMadisonWisconsin; ^6^ Department of Computer ScienceUniversity of PortoPortoPortugal; ^7^ Department of BiologyCedar Crest CollegeAllentownPennsylvania; ^8^ Department of BiologySpelman CollegeAtlantaGeorgia; ^9^ College of the Menominee NationKeshenaWisconsin

**Keywords:** debugging code2brain interfaces, evolutionary systems biology simulations, names of identifiers in code, ontology computing, programming language paradigms and naming, fundamental modes of computing, flipped programming language design

## Abstract

Names in programming are vital for understanding the meaning of code and big data. We define code2brain (C2B) interfaces as maps in compilers and brains between meaning and naming syntax, which help to understand executable code. While working toward an Evolvix syntax for general‐purpose programming that makes accurate modeling easy for biologists, we observed how names affect C2B quality. To protect learning and coding investments, C2B interfaces require long‐term backward compatibility and semantic reproducibility (accurate reproduction of computational meaning from coder‐brains to reader‐brains by code alone). Semantic reproducibility is often assumed until confusing synonyms degrade modeling in biology to deciphering exercises. We highlight empirical naming priorities from diverse individuals and roles of names in different modes of computing to show how naming easily becomes impossibly difficult. We present the Evolvix BEST (Brief, Explicit, Summarizing, Technical) Names concept for reducing naming priority conflicts, test it on a real challenge by naming subfolders for the Project Organization Stabilizing Tool system, and provide naming questionnaires designed to facilitate C2B debugging by improving names used as keywords in a stabilizing programming language. Our experiences inspired us to develop Evolvix using a flipped programming language design approach with some unexpected features and BEST Names at its core.

## Introduction

Naming is hard for humans. Because names capture a wide variety of information that may serve diverse purposes, they can be difficult to give or use. If the information associated with names changes too quickly, these substantial difficulties can overtax people's capacities to track the changes, leading some to conclude (or hope) that names do not matter. However, experience shows that names matter in many contexts where they often convey meaning critical for decision making. For example, the usually irritating “thing‐speak” (e.g., “this thing is that thing”) could indicate something wonderful, terrifying, or boring, depending on the meaning of these context‐dependent representations (or implicit names). Without context, this sentence is meaningless: “thing” could be replaced by random character labels or randomly chosen, nice‐sounding words. Random character labels are dangerous names if their meaning is not clearly documented elsewhere, but randomly chosen nice‐sounding words are even more dangerous, as they rarely prompt readers to look up the actual meaning.

**Figure 1:**
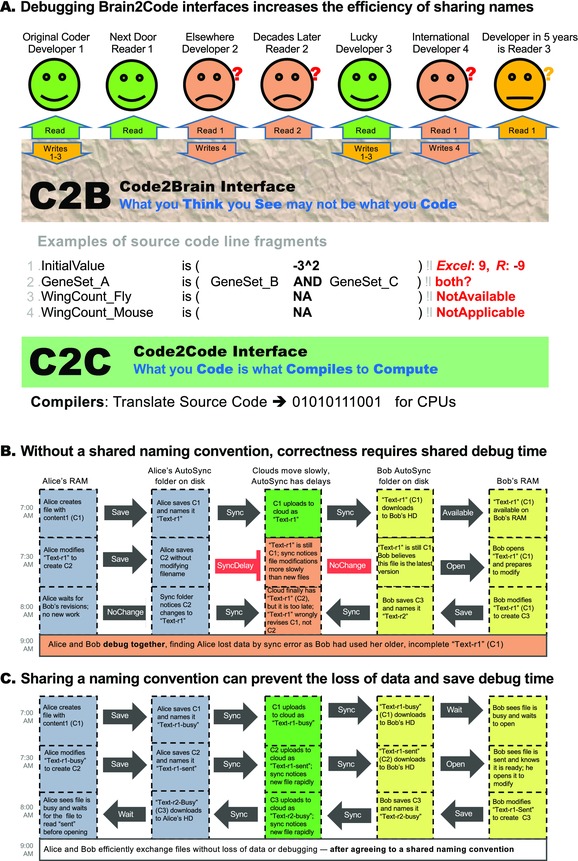
(A) C2B interfaces for writers and readers of computer programs are critical for computational science and the correct use of big data. The power of computational modeling for understanding the natural world has long been known and is essential for analyzing big data. Still, many scientists have been slow to engage with computational models. We suggest that the C2B interfaces assumed by many programming languages might carry a significant part of the responsibility as they may induce too much confusion for many scientists who are not trained computationally. It is not easy to design good C2B interfaces and near impossible to do so in isolation because of the curse of knowledge, which is difficult to escape for designers of programming languages (who need advanced programming skills to implement any language). This curse makes people forget how difficult the first steps were. As a result, they struggle to simplify problems appropriately for beginners. The only way of avoiding the resulting breakdown of communication is to debug the C2B interfaces of diverse potential user groups by comparing a language designer's ways of expressing (syntax) an intended meaning (semantics) to be implemented in a language with the meaning inferred by diverse readers of the code. This process is costly, as it involves talking a lot about communication errors in hypothetical programming scenarios of no immediate relevance to those who do the most important work for improving clarity. The goal is to highlight the blind spots in the designer's brain that tempt to prematurely accept a type system that does not well reflect the reality of those who might use the language. Poor C2B interfaces are caused by (brain) cache inconsistency, which makes naming difficult as every communicator stores local name definitions that are easily outdated. (B) Similarly, cache inconsistency can easily occur when collaborating in the Cloud. In one example, Alice and Bob collaborate in the Cloud, and file changes get lost because there is no shared naming convention (loosely based on an actual scenario observed, to our surprise). (C) In another example, Alice and Bob are able to efficiently exchange files without data loss, after agreeing to a shared naming convention (illustrating how naming and cache invalidation are two sides of one coin). In biology, these problems involve longer timescales and more collaborators, as all naming starts at independent locations with new observations. Accumulating enough observations results in naming confusion that forces a choice of costs: pay explicitly to standardize names (ensure cache consistency) or implicitly by losing research results to semantic rot. Hence, experts at the NIH recommended the development of tools that support naming.^65^

The main difficulty with implicit names such as “thing”—their ambiguity outside of a specific context—makes it difficult for others to exactly reproduce in their brains the information intended by the speaker. Content‐to‐context mismatches are not uncommon in programming and significantly degrade the quality of code2brain (C2B) interfaces discussed below (Fig. 1A). Such situations exemplify a distributed storage problem, where information travels at a finite speed (or not at all) and thus might not be locally available at a time when it would be needed to make a difference. When a brain does not receive new relevant content and has no reason to believe that the old content is now invalid, then it will intuitively apply the old content, assuming (wrongly) that it is up‐to‐date. Cache invalidation is the problem of letting that brain know that there is a reason to believe that this old content is no longer valid, and, therefore, the brain should trigger an update of the relevant content. This is a difficult problem because, in some instances, the relevant information (e.g., contexts, backgrounds, and type definitions) is never sent or received and hence travels at speed zero, as if the units in a distributed storage system were completely isolated. As noted by Phil Karlton,^1^, ^2^ “There are only two hard things in computer science: cache invalidation and naming things.”

As noted above and illustrated in Figure 1B, these two problems are different sides of the same coin, causing acute and sometimes chronic confusion in research when independent discoveries of the same phenomenon by different researchers lead to divergent naming efforts. This is widespread in biology, releasing floods of confusing synonyms. Beyond the heroic effort of disambiguation, researchers often have to work for a long time before a new phenomenon or entity is understood well enough to give it a nonconfusing name agreed upon by everybody (i.e., everybody's brain cache contains the same map from name to meaning). Without such a name, communicating meaning from one brain to another, or achieving “semantic reproducibility,” is very difficult.^3^, ^4^ We define all misinformation in situations with less‐than‐perfect semantic reproducibility (e.g., omissions, biases, contradictions, heterogeneities, ambiguities, and other distorting factors) as “semantic rot.”

Here, we argue that *naming* is far from trivial, even though it often appears so. It is essential for the semantic reproducibility of computational models in biology and for increasing the efficiency with which code can be written, understood, and reused. Trivializing naming in science might result in temporary increases in efficiency but can permanently obscure essential meanings, making it sometimes very difficult to determine the trustworthiness of results. We propose the Evolvix BEST (Brief, Explicit, Summarizing, Technical) Names concept, developed for simplifying name resolution. To place BEST Names as a concept into its larger context, we discuss the role of naming in several fundamental modes of computing to help identify and prevent naming‐related bugs. To investigate links of BEST Names to related work in ontologies, we identify “ontology computing” as defining a Turing machine derived from the functional (here called “Form Filling”) fundamental mode of computing. In Supporting Information (online only), we present reasons showing why precise naming of any nontrivial type is difficult for a group of communicators. We build on definitions of names, items, types, and contexts to argue that naming can quickly become infinitely complex and has its own version of the halting problem. Thus, despite the frequently expressed desire that names ought to be clear, precise, and descriptive, this aim is only attainable for names that represent relatively simple entities. Next, we present an overview of conflicting naming priorities, which require trade‐offs that substantially complicate naming (or add to confusion) but would be easier to reconcile by defining corresponding dialects using the BEST Names concept. We tested this in a series of naming tasks while developing Evolvix and found that BEST Names substantially eased the tension between conflicting naming priorities. The nature of these priorities was further revealed by analyzing feedback on questions designed to improve the naming process (see mini‐survey in Supporting Information, online only). In order to inspire more experimental biologists (and other noncomputing professionals) to use computational approaches, we sought to improve the naming process and developed a set of forms (Supporting Information, online only) to improve the quality of names. We report on various naming experiences and argue for an unconventional Flipped Programming Language Design approach when aiming to write a long‐term backward‐compatible programming language for a broad audience. This requires identifying a clear and intuitively understood way of expressing (i.e., the syntax for) the meaning of the concepts used in a program (i.e., the semantics). In Flipped Programming Language Design, much emphasis is placed on upfront debugging of the C2B interface of a language by heavily involving users in the design process before the pillars of the syntax and semantics of a language are locked in by implementation. The result is a much clearer, cleaner, and simpler language structure than achievable without such strong user participation.

## Naming and reproducibility in science

Semantic rot degrades scientific reproducibility at great cost to the scientific enterprise, resulting in mislabeled cell lines, irreproducible data analyses, and many other problems.^5^, ^6^ Preclinical biomedical research in the United States produces an estimated $28 billion worth of studies that are probably irreproducible for a variety of reasons, with about $7 billion (approximately 25%) caused by issues of data analysis and reporting.^5^ Statistical irreproducibility,^7^, ^8^ poor study design, and other major problems notwithstanding, experience suggest that omissions, shortcuts, and confusingly labeled names of variables and functions in analysis programs substantially contribute to the problem. As a result, many computational models and their results require a large and often prohibitive amount of work to build upon, even if they are reproducible in principle.^9^, ^10^

While bitwise reproducibility in virtual machines solves some problems,^4^ it does not address many of the floating point challenges of science (e.g., Ref. 11) and does not contribute much to the semantic reproducibility needed for extending previous computational work in a meaningful way. In fact, mere bitwise reproducibility tempts researchers to accumulate inessential complexity that can lead to the extinction of a line of work, as they omit critical information on how to extend their models. Such excessive complexity occurs when too many complicated systems build upon each other in nonintuitive ways and make the overall composition impenetrable for finite brains working in finite time. This is caused by too many indirections that make it difficult to keep track of what is represented by what, resulting in the danger of scientists standing on the shoulders of giants whose feet are slowly sinking into a swamp of irreproducibility. This can happen if computational results depend on long chains of approximations that build on each other and are difficult to check because it is not obvious which precise sets of elements (or numbers) are referred to by a given name. Such problems are also easily created by complex bioinformatic workflows, where results depend on the precise version of a tool with a given name, motivating the development of tools to address this problem.^12^

A related problem is reinvention. Science thrives on venturing into the unknown while building on previous results. However, important results in related disciplines can have unrecognizable names, and, hence, researchers often reinvent instead of standing on the shoulders of a giant on the other side of a naming fence.

### Naming challenges for integrating models in evolutionary systems biology

Mechanistic evolutionary systems biology (EvoSysBio)^13^ requires the integration of numerous diverse and complex computational models, each challenging enough to justify publication(s) and, hence, often built by different researchers using different tools. Some of these EvoSysBio models can be easily built by modularization approaches that explicitly wire only the connections between models, hiding inside details irrelevant for model interactions. Other models concern different subsystems that nevertheless interact in a shared physical space, which require synchronizing all parts from all models that access this shared space, such as a cell or an ecosystem. Technically, such models can also be built using a modularization approach; however, since almost everything in cells is connected to almost everything else, the cost of a modularization approach increases dramatically for large numbers of subsystems in a shared space: two models with five shared parts may only require five send–receive channels for each model (20 connections with potentially distinguishable events). However, it is never really possible to stop tinkering with them as biologists keep adding new biochemical pathway models that add to our understanding of a cell. There is a multiplicative element inherent to such scales: adding the 101st model will require communicating changes in a commonly shared molecule, such as adenosine triphosphate, the cell's energy currency, with 100 other models that could also affect its amount, requiring at least 100 explicit wirings in two directions. These considerations have inspired proposals to put the semantics of molecules in their name,^14^ so that combining different pathways in simulations of a cell becomes easy, as the same molecules in a shared physical space have identical names—as in a namespace.

The BEST Names approach presented was inspired by this very use case, as the range of biological systems amenable to rigorous mechanistic evolutionary systems biology analyses^13^ will be greatly reduced if no efficient methods can be found for fighting semantic rot from problems such as poor naming. Naming is trivial for three items, doable for 300, and then quickly becomes a swamp of complexity for larger numbers of items. This problem is compounded by the inessential complexity of poor computational tools that produce results that are mostly right enough (for the use cases of those who wrote them), but do not support building deeper computational hierarchies. Using such approximate tools without integrated error handling degrades research to finding needle‐like errors in haystacks of data, robs researchers of the time to make genuine discoveries, and forces them to engage in costly and frustrating naming exercises to determine if a result should be labeled “reliable” or “numerical artifact.”

Recently, a model has been constructed for simulating all molecules in a single cell.^15^, ^16^, ^17^, ^18^, ^19^, ^20^, ^21^, ^22^ Producing this one‐cell model required a large team and much time and effort: data bases were constructed for storing names, synonyms, and literature references. From this material, the actual list of parts was collated for simulation. This is not only a naming problem because all these entities require a name in the simulations, but also because they are known by many synonyms that require some form of entity resolution to merge them into a single identity, if that is indeed what they are within a cell. Entity resolution is the well‐known computational problem of identifying matches between records that look very different. Like the general problem of naming itself, entity resolution comes in many guises, such as taxonomic species description, ontology construction, library organization, linking, and others.

**Figure 2:**
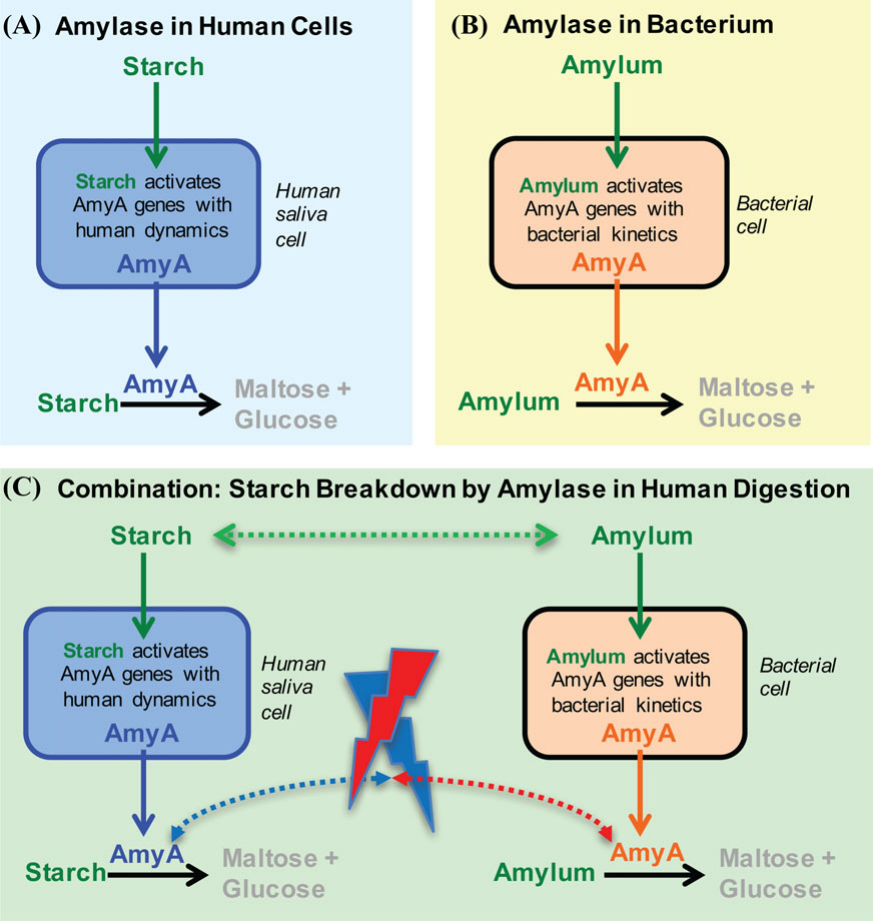
The challenge of combining independently developed models in systems biology, sometimes using different nomenclatures for the same entity (e.g., starch and amylum), or the same name for rather different entities (amylase is either under human or bacterial control resulting in very different time courses, as bacteria grow much faster than human cells^23^, ^24^, ^25^, ^26^, ^27^, ^28^, ^29^). Here, we illustrate a hypothetical scenario, where a model of starch breakdown in human saliva (A) was independently developed from a model of amylum breakdown by bacteria (B). How starch is broken down during human digestion can be better understood if researchers integrate these models (C), which could happen either by building a “supermodel” that manually wires each relevant change from one to the other or by combining them into a common namespace and letting the compiler do the wiring by interpreting the semantics encoded in the names of the simulated parts.

At the core of these naming activities is the goal of unambiguously identifying a given entity or meaning in a way that makes it easy to reference. An important difficulty in such work is illustrated in Figure 2 using a simple example of a biochemical toy model, where careless naming can easily derail simulation results. Briefly, the homologous enzyme called amylase (one of about 150 synonyms) is produced by human saliva cells and bacterial cells, each with their own genetic regulation.^23^, ^24^, ^25^, ^26^, ^27^, ^28^, ^29^ Both cell types break down starch, which is identical to amylum in the bacterial model. If they meet in the human digestive tract, the bacteria may grow (and produce more amylase), while human cells would be too slow for growth on the time scale of a meal. The separate gene regulation networks of both cell types require strict separation of amylase production, while both process the same substrate, starch (i.e., amylum). Resolving the naming problems in this example by hand is simple, but the same problems easily spiral out of control at the level of a cell, with its hundreds of thousands of types of molecules. The biggest challenge is to determine whether any simple omissions, as shown in Figure 2, hide among thousands of reactions that have been wired manually. This challenge of tracking synonyms is what the BEST Names concept has been designed to address efficiently, without the overhead of external databases. The diverse dialects enabled by BEST Names can be used to implement diverse naming strategies that prioritize different aspects of the named entity, while all pointing to the same single point of reference. Such simple (but not necessarily small) name trees are a powerful tool for linking complex simulation models unambiguously to real‐world entities (or at least highlight potential problems). If one single name is like a single thread, BEST Names can easily be woven into an extremely strong rope with many threads that will hold under many circumstances.

In online Supporting Information, we discuss why naming is essentially an infinitely complex problem with many diverse priorities that could benefit from being addressed one after another (each deserving a dedicated name, if needed). This avoids the need to satisfy complicated trade‐offs and different audiences all at once by either attempting to find the “perfect name” or choosing a random name, accepting a misleading name or remaining blissfully unaware about how confusing the corresponding name actually is for others (see also Table 1).

**Table 1:** Questions on aspects of naming complexity

**Aspect of naming**	**Property of**	**Modified by**	**Affects**	**Potential questions**	**Potential answers**
Explicitness	Part, whole	User relevance and ease; authority defines standards	Users	How many interesting details from lower levels of content are packed into the Name?	List some or all elements of the set that is being named
Clutter	Whole	As above	Users	How many uninteresting details from lower levels of content are included?	Listing the elements of subsets of subsets … makes for tedious reading, even though it increases precision (see “Form Filling” mode of computing)
Audience (intended)	External	Experience of the naming authority, feedback	Perceptive naming authorities	How will the names be used? As predicted by the authority?	Differ for different authorities
Audience (actual)	External	Quality and usefulness of names	Perceptive naming authorities	Which names are accessible for actual users? Which support exists (dictionaries, name trackers, immutability, etc)?	Differ for different authorities and may also depend on the audience (e.g., if allowing for different BEST Names dialects)
Mode of computing (Table 2)	Whole, external	Machine type	Names users, authority	Which modes of computing are used for naming? Is it allowed to mix modes when naming, despite the potential confusion?	Name contains any address from a locally linear space, or all content, or content fragments, or any queries for element subset conditions, or a mix
Assigning	Whole, external	Item or pattern for which type is unknown	Authority, users	Is the Name assigned to an Item or Pattern that is observable in the real world? What is the most appropriate name for this content?	Searching for good names can take time. Resulting names can be temporary or local until they are centrally standardized (to avoid distributed naming)
Inventing	Whole, external	Invented type for which no real‐world equivalent is known	Authority, users	Which name best describes the properties of a Type or Pattern that is not observable (i.e., Fiction)?	Types exist without items. They are either made up or not yet identified. Their names reflect properties
Naming authority	External	Local, distributed, or global users	Naming process; users	Who names newly found content? Who can list all local names in a context, and how many at a time? Who names the authority and how is it found?	Authorities can be context defined (address)‐, self‐appointed‐, other‐appointed‐, self‐naming‐, other‐naming‐, local‐, distributed‐, global‐, absolute‐, etc.
Label purity	Whole	Naming authority	Stability	Pure names are pure labels without any other meaning. How independent is the name from anything that is being named?	Names can depend on storage location, content, context, type, or specifics of these. Pure names are free from any interpretation other than “label of content” (like a collision‐free hash key pointing to content)

Note: Many aspects of naming contribute to its complexity: a multitude of competing perspectives and potential criteria, whose importance is often only clear with hindsight. To help navigate naming complexity and facilitate more conscious naming decisions, we present some questions of potential interest. An exhaustive list is beyond the scope of this study and would also have to investigate address‐names, relative names, naming‐ambiguity, brevity, name‐usage‐complexity, name‐search‐complexity, maturity, namespaces, absurdity, versioning, intuition, standardization, and many other aspects that can affect the *Whole* name, only a *Part* of the name, or something *External* to the character‐sequence of the name itself (as indicated in the column “Property of”). In this table, “users” refer to persons or programs that use a name that had been given by a naming “authority.”

## BEST Name dialects^1^

**Figure 3:**
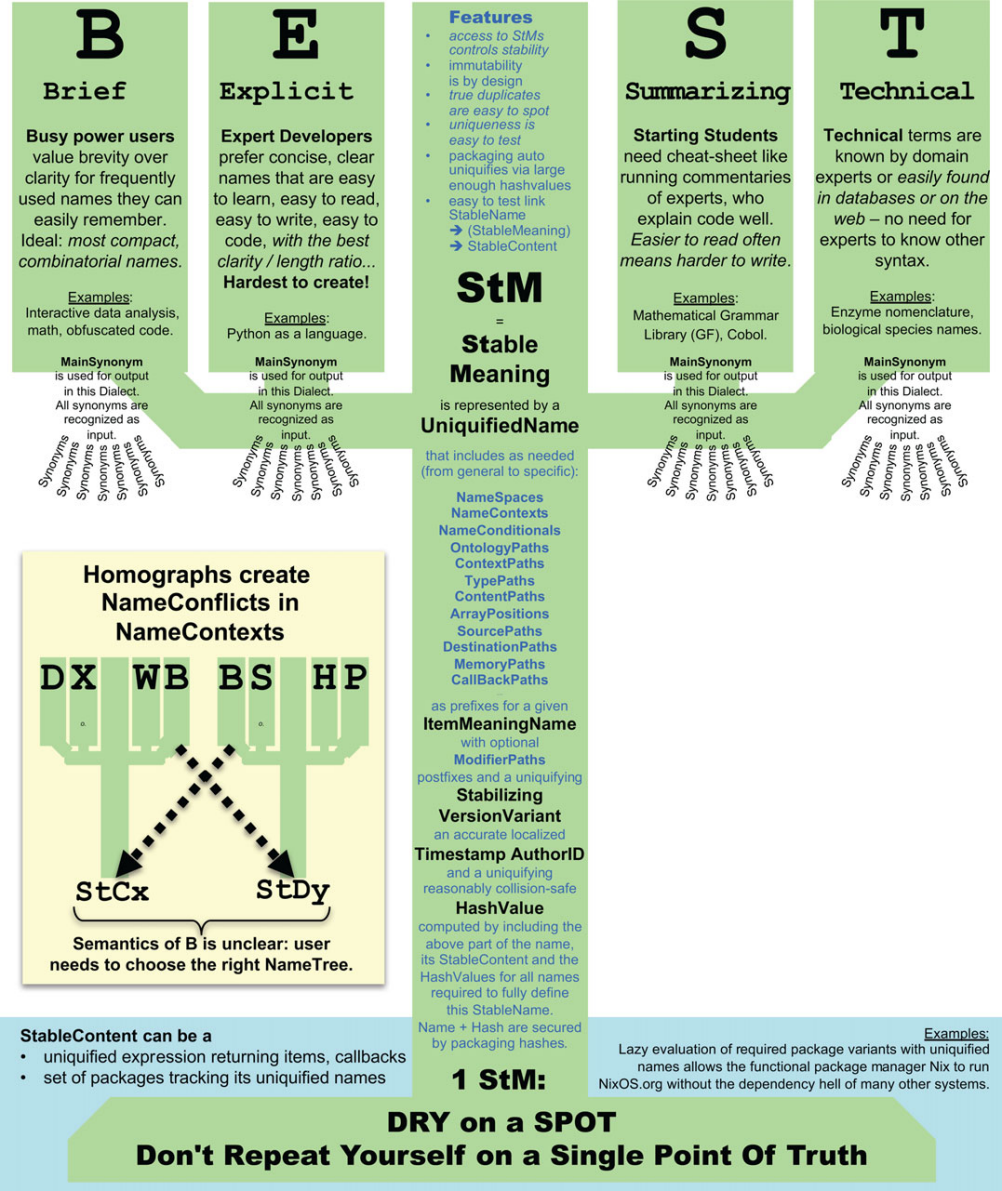
BEST Names can simplify naming in complex biological models, if all names and synonyms in a context are consistently mapped to exactly one StableMeaning (StM) as implemented by pointing to one StableContent. The insert shows what goes wrong if two BEST Names trees that should be separated happen to not be, due to a naming conflict: in that case, the synonym “B” would ambiguously map to two StMs. There is no point in biology to have computers automatically resolve this, because by the time a computer reaches this point, it will have completed the part that is most difficult for humans: finding the needle in the haystack where this miswiring actually occurred.

A brief overview of BEST Names is provided in Figure 3 and in the acronym itself, describing four dialects reflecting different types and levels of expertise in any area:

**Table:** 

Brief	for **B**usy power users who value brevity to reduce typing
Explicit	for **E**xpert developers expecting ease of writing and reading
Summarizing	for **S**tarting students who need a cheat sheet
Technical	for **T**echnical standards linked to, as needed

More dialects can be defined as needed, but only the Explicit Name is essential, as it provides the best compromise between readability and brevity (and is similar to the single name that other languages accommodate; thus, BEST Names do not enforce a rigid naming scheme). All SynonymNames within a dialect, as well as all BEST Names dialects within a namespace, point to the same uniquified StableMeaning (StM), which is defined by StableContent that represents all relevant semantic details (denotational semantics, structural semantics, algebraic semantics, operational semantics, or any other approach to semantics, as defined by the context, implementing the StableContent associated with a BEST Name). Exactly one StM (pointed to by names) links to exactly one StableContent that is unchangeably linked to its version information and a uniquifying hash‐code to ensure that it follows the DRY on a SPOT principle (Don't Repeat Yourself on a Single Point Of Truth).

Technically, BEST Names in a given namespace are managed in a data structure that provides a local key‐value map of each name to its unique StableContent. If BEST Names synonyms in such a namespace map to more than one uniquified StM, then a naming conflict has occurred (see homographs in Fig. 3). A local collection of StMs provides the equivalent of a BEST Names dictionary. Various efficient data structures now exist to support dictionary‐type collections that maintain a useful degree of order.^32^ See earlier work on BEST Names for a draft of important implementation functionality.^3^

### Precursors of the BEST Names concept

After the first introduction of BEST Names,^3^, ^30^ closer inspection revealed precursors that had inspired its development. For example, we used something similar in the evolution@home project Simulator005^33^, ^34^, ^35^, ^36^, ^37^ for disentangling the longer meaning of observed modeling variables from the Brief Names used in concise simulation reports, which demonstrates that equivalents of BEST Names could be used in other languages, albeit requiring additional implementation work.

A combined variable name had previously been proposed to make it easy to see simultaneously which equations in systems biology models an identifier might refer to and what the actual biological meaning was.^38^

While not exactly an identifier in code, languages such as General Algebraic Modeling System (GAMS; https://www.gams.com) or Python have simple syntactic additions that allow recording the equivalent of a Summarizing Name; Python's DocString can also handle arbitrarily large texts and has in ReStructuredText a powerful documentation system.^39^ Systems for combining code and documentation have a substantial history, which includes tools that process a mix of code and comments into a presentation optimized for readability.^40^

The long history of complex naming efforts in biology repeatedly inspired diverse dialects for coordinated naming of new discoveries. For example, naming epidemiological Ebola virus samples^41^ recently defined dialects equivalent to Brief (“Abbreviated name”), Explicit (“Shortened name”), Summarizing (“Full name”), and Technical (“<DEFINITION LINE>” and “GenBank Accession Number”).

### BEST Names in more detail

BEST Names capabilities in a modeling language would make it easy to highlight the problems in the abovementioned amylase example (Fig. 2), so that researchers could manually fix them efficiently and no longer have to search for them like a needle in a haystack. Computers cannot fix these problems easily, but they can highlight them easily by using ideas like the BEST Names concept.

An ideal name conveys the essence of a semantic unit to readers and allows them to maintain focus by avoiding disturbances in reading flow, such as the need to look up meanings or excessive visual clutter from unnecessary explanations. Readers at different levels of familiarity with the underlying concepts will require different details to protect the readability of a code. The conflicting needs of a diverse user base are impossible to meet with just one way of representing names: this conflict is best resolved by providing multiple identifiers for the same stable meaning. Thus, for developing Evolvix, we have given up the goal of finding single names that work for all users. Instead, we will use BEST Names explicitly to enable communication between diverse audiences by supporting dialects corresponding to users’ natural usage and topic familiarity. As all dialects must unambiguously map to identical StMs represented by corresponding uniquified names, there must be no doubt about what a particular identifier might mean at any given point in time. Key dialects are described next (Fig. 3).

### Overview of dialects

#### Brief Names

Brief Names are for experts or specialists who use certain functions so much that they value the option to memorize more and type less. The Brief dialect values brevity over readability. An effort should be made to use common abbreviations, associations, and mnemonics, while eliminating as much semantic ambiguity as possible.

#### Explicit Names

Explicit Names are for programmers who prefer something that is both short and memorable. They do not need introductory information as they are past the beginner's stage and now look for more efficient coding. Any explicit naming scheme should exploit opportunities for combinatorial regularity as much as possible to simplify learning. Explicit Names should combine the strengths of Brief and Summarizing Names.

#### Summarizing Names

Summarizing Names are for beginners who often need to learn related underlying concepts. These names must be as didactic, precise, and explanatory as possible and should be field tested on beginners to improve clarity. The emphasis of Summarizing Names must be an introductory presentation that avoids unnecessary jargon. Examples that “describe what the name stands for” can be found in the online Supporting Information (see Figure S3 in Supporting Information for the Brief Dictionary of the Project Organization Stabilizing Tool (POST) system).

#### Technical Names

Technical Names are for experts and should reflect the name of the corresponding semantic unit in an appropriate ontology. They should enable a technically literate reader to quickly identify key concepts at hand without knowledge of other syntactic details, which are irrelevant to the problem domain.

#### Synonyms

Synonyms can be defined in arbitrary quantities for any BEST Name, as long as they do not create naming conflicts (an identifier pointing to more than one StM). The position of “first synonym” is special in that it must hold the default identifier that will be used when a representation of its StM is requested in the corresponding dialect.

#### Mixed use

Mixed use must be possible at all times for BEST Names, such that any identifier from any dialect can be used in any sequence that would be permitted when using only one dialect. The integrity of identifiers must be respected by any implementation in the sense that identifiers can occur as fractions of other identifiers without causing name clashes.

#### Translating between dialects

Translating between dialects should be easy for any system implementing BEST Names, such that any synonym from any dialect enables access to (1) all synonyms of its StM in all dialects, (2) all data and functions in its StM, and (3) at least one form of linkable ID that facilitates quick StM access for external code.

Preferred synonyms are used for representing the dialect in output (there can only be one preferred synonym per dialect). Unpreferred synonyms are fully recognized as preferred synonyms, but are not actively used when producing output in their dialect. The resulting name trees of all StMs must always remain independent (Fig. 3).

#### Defining new dialects

Defining new dialects for special purposes is encouraged, as long as they do not compromise other required functionality, all synonyms in all dialects remain unambiguous in their corresponding namespaces and this can be checked automatically. In Evolvix, the names of user‐defined dialects are not allowed to consist of only a single letter (reserved for future expansions of the Evolvix standardized naming system beyond “B,” “E,” “S,” and “T”), and must not clash with any letter‐case combination of the names “Brief,” “Explicit,” “Summarizing,” and “Technical.” We recommend a similar convention for other systems adopting the BEST Names concept.

#### StableMeaning, StableNames, and StableContent

An StM is the underlying meaning represented by all of its BEST Names synonyms. StMs can map to any concepts or details from an ontology or be defined by the local context. StMs can be further documented in associated info texts. The abstract StMs that BEST Names point to can be more fine‐grained than the concepts typically used in ontologies. Ultimately, StMs are defined by combining whatever definition of semantics the language compiler will associate with them by providing corresponding StableContent (e.g., the operational semantics of the code they point to, the structural semantics of the data they point to, and the notational semantics of names or other text details that document meaning in human readable form). StableNames (see Fig. 3 for composition) are the tangible representations pointing to abstract StMs, which are implemented by the StableContent that represents all relevant semantic details (denotational, structural, algebraic, operational, … semantics as defined by the context). This mirrors the simple, intuitive, real‐world interpretation that a name is a box; meaning is what is inside.

## Experiences with BEST Names

So far, we have a number of naming projects, either ongoing or near completion, which have used the BEST Names concept extensively, including a renaming of the 16 Boolean truth functions and of aspects of fundamental modes of computing. The most elaborate use case produced the StabilityCodes of the POST system presented online as Supporting Information.

### The potential of BEST Names

We focused on biological synonyms in modeling, but complicated naming trade‐offs exist elsewhere in programming, as also discussed in another thorough analysis.^42^ Trade‐offs vary substantially, hence BEST Names do not require synonyms for all four common audiences defined here, nor for all words in a language or variables in a program.

BEST Names can act much like glossaries in textbooks: they do not define all words, but collect those most likely to trouble target audiences and guide their interpretation and usage in a particular context. Knowledgeable readers do not have to use glossaries, but for newcomers they extend a warm welcome into the field. Similarly, BEST Names can offer flexible support for any of the four common audiences and may accommodate more specialized audiences via the definition of additional dialects.

The only requirement is that at least one name is mapped to each StM; all else is optional. This makes BEST Names no more difficult than naming in other programming languages, yet it offers the flexibility to include more representations of meaning from different perspectives and thereby increases semantic reproducibility.

Deeply integrating this simple and versatile concept into the syntax, parser, and compiler of a language could further simplify various forms of name processing (e.g., when formatting for different purposes). As the general design of Evolvix (http://evolvix.org) is being reviewed to incorporate experiences from previous language implementations^43^ before committing to a specific implementation, we continue to find BEST Names useful for finding short, medium, or long keywords for beginners and experts.

The potential of implementing BEST Names in the core of a general‐purpose programming language can be substantial. BEST Names can help find better names during development and thus ease refactoring. Allowing for dialects had a noticeable liberating effect in discussions of Evolvix syntax design, substantially affecting long‐term design decisions. The BEST Names design is one of many examples where the biological background that drives the development of Evolvix inspired a feature that makes Evolvix a programming language that is particularly attentive to the needs of biology. The needs of biology are likely to present more opportunities to improve general‐purpose programming capabilities.

## Ontology computing, BEST Names use cases, and naming in fundamental modes of computing

Table 2 describes four fundamentally different modes of computing (MOs) that have been proven to be equivalent in their computational power (but not equally useful for all problems). In this context, it is possible to define ontology computing as a derivation of functional programming. We deliberately chose unconventional names to help readers think about these MOs in new ways and to identify the role of names (and naming bugs) specific to the corresponding computation.

**Table 2:** Ontology computing and other modes of computing affect the role of names in programs and bugs

**Modes of computing**	**Storage space navigation**	**Content data (longer term)**	**Code changing data**	**Execution data (shorter term)**	**Role of names**	**Bugs related to names**
**Structured Commanding** Store data and computing instructions in a sequence of memory locations, then follow the sequence of instructions (may include conditions and loops) and load, manipulate, or save data accordingly to get solutions.	**Arrays** In its most basic form, all content is stored in a very long array of bits or bytes that is subdivided in various ways.	**Array elements** The content of array elements contains the data that are being processed. Variable names can be seen as more readable names for memory addresses.	**Code sequences** Some of the available data are changed by executing sequences of commands in scripts or functions.	**Starting pointer** Execution of all computing starts by following a pointer to a well‐defined function or script, which will point to the next one, until the task is completed (or interrupted).	**Array locations** Names are human readable labels pointing to a fixed‐size box position with content of interest in storage space. Much work goes into storing content exceeding fixed box sizes.	**Pointer bugs** Variable names pointing to the wrong box usually return the wrong content. Function names pointing to the wrong address lead to bugs or security holes. If content does not fit the allocated box, names become misleading and the underlying assumptions of the code are violated.
**Functional Form Filling** Define the names of all wildcards (that can be replaced by anything), the content of all forms, and the inputs to replace the wildcards. Execution replaces wildcards in forms by filling in specified input in the given order.	**Namespace** All possible names for wildcards and forms build a namespace that can be used for storing anything, irrespective of size.	**Forms** All content is represented as forms that contain a combination of previously filled‐in content and wildcards	**Fills** Forms are modified by filling the places of wildcards with specified content. This can create new forms to fill.	**Starting forms** Filling the initial forms with initial content triggers the rewriting of all other forms in specified order to deliver the last rewritten form as output.	**Abstract roles** Names identify patterns in dynamic content with widely varying sizes. Thus, costs of using static names or retrieving content may be unpredictable.	**Replacing bugs** Consequences of too many substitutions executed on top of each other can be unintuitive. Bugs occur when the formal definitions specify unintended substitutions.
**Ontological Dictionary Defining** A special variant of form filling, where the entries of an ontology are wildcards, and their definitions are forms, possibly using other wildcards. Computing is defined as replacing all wildcards to get a fully explicit definition for a dictionary entry.	**Namespace** All possible character sequences for entries of the ontology's dictionary define its namespace. A position in space is occupied if a name definition is provided. Conditional definitions can provide subspaces for resolving ambiguities.	**Term definition** A term name is equivalent to a storage address, where the content matter behind the name is given by its definition. If context dependent, this houses multiple definitions, as found in dictionaries for words used in different contexts.	**Cross links** A term defined by other terms that are defined by still other terms creates a network of links that defines the code to be executed. Cross links easily create parallel and/or recursive structures that can make it as difficult as the halting problem to know which definitions are circular (leading to infinite loops).	**Retrieving term definitions** Each explicit definition computes a full execution tree. The levels of nesting equivalent to generalized computing depend on how many definitions are expanded or contracted. Aiming to compare similar terms from independent ontologies of the same area thus easily defines data structures too complex to complete comparisons.	**Definitions** Names are keys to dictionaries and ontologies. Naming is complicated by the large shared name‐spaces, often with controlled term lists to minimize accidental ambiguities. For defined ambiguities, all conditions for resolving the ambiguities are given.	**Ambiguity bugs** Naming errors cause two major problems: accidental ambiguity in use (from typos or sloppy use of terms in other definitions, resulting in dead pointers) and a mismatch between the name and definition used for a given term (by ignoring key C2B interfaces in users). Such naming errors can result in dead or misguided links, contradictions, unnecessary ambiguity, and circular definitions that obscure meaning.
**Logical Solution Isolating** Declare the input of all potential solutions, then apply all relevant constraints to distinguish actual solutions from nonsolutions.	**Sets** Sets can store elements and subsets. Thus, the identities of existing sets, subsets, and elements define a nested storage space.	**Elements** Elements can be either basic or composite, where the latter define data in the form of subsets.	**Query logic** Sequences of nested queries that successively narrow down an original broad set of solutions until the final answers are isolated.	**Requirements** It is often faster to start execution with the ultimate requirements and proceed by evaluating only those needed to meet these. Forward execution often computes unneeded details.	**Query results** Names identify combinations of sets and elements directly or indirectly specified using some logic formalism; great for querying sets, but not for numerical computing.	**Set content bugs** Poor name choices make it difficult to determine the boundaries of sets queried; errors occur when elements are included that should be omitted and vice versa. Poor naming of infix operators with perplexing precedence rules can further obscure the content of a set.

**Modes of computing**	**Storage space navigation**	**Content data (longer term)**	**Code changing data**	**Execution data (shorter term)**	**Role of names**	**Bugs related to names**
**Concurrent network traffic** Define the network by listing all nodes, all links between them, and all actions affecting the network. Simulate time by executing all ongoing actions concurrently to change the network until it represents the solution.	**Nodes, links** The space of networks is defined by possibilities for the existence of nodes that store data to be acted on with other nodes; whether concurrency is physical or conceptual is irrelevant.	**Stable network between actions** Data are stored in those nodes and links that exist locally. This inherent locality is difficult to recognize in some algorithms, but it conceptually exists in all networks.	**Concurrent patterns** Many actions executed by nodes form repeatable patterns of change in their local memories. Describing concurrent patterns efficiently in code can take many forms.	**Transient states** Concurrent patterns are defined as possibilities realized only when the necessary content is in place. The initial state of networks determines possibilities, making forward simulations a method of choice.	**Labels in transition systems** Names of nodes and actions define implicitly the links that govern the simulation of concurrent processes that might interact. It can be challenging to track relevant names when they are created dynamically.	**Tracking bugs, typos** If minor changes in names create major effects and no support exists for catching typos, then naming bugs rewire networks to become misleading. Concurrent networks are harder to debug, if identifiers can change meaning with contexts.
**Physical world** Physics framed as computing (to learn from its stability): store matter in space and define how sequences of actions allow it to change over time. Use energy as transient storage that affects what happens next.	**Space** Positions in 4D space–time define potential storage places with limits for the amount of content matter that can be stored there. Each 4D position can be seen as an implicit name.	**Matter** Content can be seen as networks of specific atoms that occupy given positions in 4D space–time. Each content matter can be an implicit name.	**Time** Time can be seen as all potentially occurring action patterns defined by how they would change implicitly named matter in space, contingent on all conditions being met. Each timed localized specific potential action pattern can be seen as an implicit name.	**Energy** Energy can be seen as the present‐time enabler selecting some of the many potential actions by meeting their energy requirements. Thus, each timed localized specific potential action pattern that has been enabled can be seen as an implicit name (usually called “historic event”). Energy is temporary storage as it only exists in transit.	**Coordinates, alternatives** Physics defines four basic types of implicit names (timed position, content matter, potential, and executed actions). These run the physical Universe and suffice also for concurrent Networks Traffic. The implicit types enable infinities of combined derived names for more complex patterns (or “traits”).	**Delineation bugs** Lower level implicit names like relative positions can generate attempts to access elements beyond the limits of an array; such bugs can be hard to find without automated help. As communicators can dynamically (re)name anything at will, naming is superinfinitely complex and extra care is needed for agreeing on the same delineations.

Note: Columns highlight parallels between the modes of computing given in rows, such as (imperative) Structured Commanding, Functional Form Filling, Ontological Dictionary Defining, Logical Solution Isolating, Concurrent Network Traffic, and the Physics World (matter–energy in space–time). This very foundational view enables a new perspective on the role of names in each mode of computing and the opportunities and dangers names present for all programmers. While proofs show that infinite time and resources allow each mode of computing to accomplish any task another mode can do, in practice each mode greatly simplifies some types of tasks, but not others. Names were chosen to provide a fresh perspective on these tasks, some of which are particularly fundamental to computing (Structured Commanding, Form Filling, Solution Isolating, and Network Traffic). Most general‐purpose languages combine aspects from these fundamental modes of computing to facilitate the efficient implementation of diverse complex scenarios, but support for some aspects is often retrofitted or limited. A more streamlined and thorough integration of fundamental modes of computing could simplify programming and the construction of many derived modes of computing such as Ontology Computing (derivable from Form Filling) or simulating the Physics World (derivable from Network Traffic).

BEST Names can be seen as a simple entity‐resolution forwarding device, such as an alias or link, as known for a long time in programming languages (e.g., typedef in C++), albeit with the same rights as the original name.

BEST Names are not made to be computationally complete, such as the big ontologies in biomedical science (e.g., SNOMED and UMLS)^44^, ^45^, ^46^, ^47^, ^48^, ^49^, ^50^, ^51^, ^52^, ^53^, ^54^ and others that support ambiguity resolution, which requires the management of conditional environments and essentially makes these ontologies Turing complete.

Many ontologies also allow for synonym resolution. For example, SNOMED and NIH UMLS^44^, ^45^, ^50^, ^52^ (https://www.nlm.nih.gov/research/umls/; http://www.ihtsdo.org/snomed-ct), with their metathesaurus and semantic network, are designed to help medical professionals quickly navigate medical synonym complexity in stressful situations by listing frequently used synonyms and all their alternative meanings. Such real‐world demands require support for synonyms that are clear to humans, but ambiguous for computers, since decisions in a clinical context leave little time for formal synonym resolution. Thus, such systems necessarily support multiple levels of synonymy.^52^ In contrast, BEST Names can only minimize ambiguity by asking modelers to review all names that appear not to be unambiguous (see definition 12 and definition 14‐3 in Reference 52 for potential definitions). As in other ontologies, these richer synonym resolution tools require maintaining additional systems as they allow ontologies to be transformed into full Turing machines, as pointed out in Table 2. Thus, the comparison of ontologies^50^ can be an undertaking that is as predictable as the halting problem, if the ontologies are not trivial and contain significant ambiguity^52^ or circular definitions.

Powerful ontology systems are the tools of choice if a relevant ontology already exists (e.g., at http://protegewiki.stanford.edu/wiki/Protege_Ontology_Library), but that is not always the case when building smaller systems biology models. In fact, these smaller models might aim to link to different ontologies in order to properly identify the various parts in the system they model. In this case, BEST Names can facilitate linking to entries in other ontologies by allowing these to be added as synonyms. However, enforcing semantic types and relations is beyond the scope of the BEST Names concept and best accomplished by a corresponding virtual machine or type‐checking compiler.

When the BEST Names concept was first presented, the problem of semantic reproducibility was highlighted.^3^, ^4^ BEST Names address this problem, in part, since different synonyms can communicate different semantic aspects, but all have to point to exactly one StM. The strength of BEST Names is that all of them can be used interchangeably in the synonym namespace as long as they remain unique (or have been uniquified, i.e., artificially made unique by appending a pure name long enough to guarantee uniqueness).

Their use case focuses on the diversity of biological names in modeling, which requires a simple and near ubiquitous way of entity matching on the basis of limited information. Since this type of match is done before executing a program, it is easier to involve users in providing more helpful information. We aim to facilitate the efficient meaningful combination of different models developed by different researchers. A similar aim is driving diverse work in the systems biology modeling community.^13^, ^14^, ^15^, ^16^, ^17^, ^18^, ^19^, ^20^, ^21^, ^22^, ^55^, ^56^ It also motivated the development of the concept of “semantics‐based adaptable interface modularity,”^14^ which shares with the BEST Names concept the idea of actively managing names to enable the efficient merging of models. Both methods aim to find common stable meanings from different models, thus allowing the names from both models to be treated as synonyms.

The synonym problem is being addressed by diverse frameworks for data integration.^57^, ^58^, ^59^, ^60^, ^61^, ^62^ For example, entity matching can be interpreted as identifying synonyms from different sources.^57^, ^60^ There are certainly many problems in biology where more database‐intense approaches of entity resolution are appropriate; however, the BEST Names concept appears to be much more lightweight and easier to use from within source code. Provenance systems can also resolve synonym naming difficulties by tracking various names of items in different contexts (see the new W3C PROV standard with formally defined semantics^63^). Again, the full complexity of allowing for arbitrarily nesting groups of user‐defined provenance information, as allowed by the PROV standard, can be seen as a separate programming language in itself; such complexity does not always help to solve the much simpler problem of “unambiguous synonyms only.”

Naming is complicated, and various other discussions have indicated many reasons,^1^, ^2^, ^3^, ^12^, ^14^, ^17^, ^30^, ^38^, ^39^, ^40^, ^41^, ^42^, ^43^, ^44^, ^45^, ^46^, ^47^, ^48^, ^49^, ^50^, ^51^, ^52^, ^53^, ^54^, ^55^, ^56^, ^57^, ^58^, ^59^, ^60^, ^61^, ^62^, ^63^, ^64^, ^65^, ^66^, ^67^, ^68^, ^69^, ^70^, ^71^, ^72^ such as BEST Names do not aim to solve all naming problems (a task almost as complex as describing the world), but they do aim to help the writers and users of code to navigate the trade‐offs that are a necessary part of any attempt to communicate semantics by choosing a name. Also, the possibility to link to an StM that is frozen in time and could thus be stored and reactivated as needed creates the possibility to use ideas from the Nix functional programming language,^66^ in order to further improve long‐term backward compatibility.

## Perspective: code readability, stability, and flipped programming language design

The decision to support BEST Names throughout Evolvix and all its simulation and modeling aspects^3^, ^30^, ^31^ has prompted revisits of bigger naming questions in languages: how to remove the danger of absolute names, separate pure from impure names, construct ontologies, integrate imported data, facilitate local naming by independent authorities, and improve support for associated information texts, meta‐data, and literate programming.^1^, ^2^, ^3^, ^14^, ^30^, ^39^, ^40^, ^41^, ^42^, ^52^, ^53^, ^54^, ^55^, ^56^, ^57^, ^58^, ^59^, ^60^, ^61^, ^62^, ^63^, ^64^, ^65^, ^66^, ^67^, ^68^, ^69^, ^70^, ^71^, ^72^ Writing readable code is an art.^69^ We have come to think that BEST Names can help improve the state of this art.

The BEST Names concept was inspired by the very real need of modelers to find a way to track the meaning of the many variables in systems biology models.^3^ We also realized that newcomers prefer longer names, whereas power users do not like to type that much (and it is surprising how quickly newcomers can turn into power users, simply by repeating the same task many times). The comments we gathered in our small informal survey on naming seem to confirm these length preferences (see online Supporting Information). If unresolved, this fundamental tension will relegate any language to be either a basic beginners’ language or short and fast code for power users, remaining forever cryptic to everybody else. This naming tension is likely to keep blocking the path of beginners’ languages from developing into power‐user tools, thus adding inessential complexity^71^ to computational biology's necessarily heterogeneous audience. BEST Names provide a systematic way of resolving this tension.

Using this conceptual tool, we have been aiming to design a language that is powerful enough for sophisticated computation biology, yet simple enough to have a gentle learning curve and inviting enough for biologists who would otherwise never dream of modeling or programming. Eventually, we discovered that, despite the mantra of professional programmers to “use the right language for the job,” we could not find a general‐purpose programming language that was designed by biologists for biologists facing biology's complexities. While there exists a wide variety of specialized tools and domain‐specific languages (e.g., see http://SBML.org), none provides a long‐term backward‐compatible platform for reliable computing in biology, as needed for making progress in evolutionary systems biology at a reasonable speed.^13^ It is not impossible to use other general‐purpose languages, and in principle computational biologists could also use machine code as they did initially. However, the success of scripting languages like Python has shown that higher level abstractions tend to make programmers much faster when writing code. Evolvix can provide additional levels of abstraction that greatly simplify repetitive general‐purpose programming tasks in the context of biology. For biologists, such efficiency means built‐in support for importing diverse data, analyzing sequences, comparing statistics, modeling replicators (that could be nested), and simulating chemical reactions, ecological systems, and population genetics—all within a single model that is easy to modify and provides enough numerical accuracy for tracking extremely rare events (and automatically highlights when higher precision may be needed). In contrast, currently, biologists either program around the lack of such features in a general‐purpose language, start to write tools that address their needs, or simply pick another question. Thus, current computational biologists spend much of their time fighting inessential complexity^71^ to the detriment of research time.

These inefficiencies motivated the decision to transform the special‐purpose modeling language Evolvix (efficiently simulating mass‐action system time series^31^) by adding general‐purpose programming capabilities without sacrificing clarity, expressivity, or long‐term backward compatibility (see http://evolvix.org). The discovery of BEST Names was an important milestone in this effort, since it allows a language to support noncomputational biologists and comp‐bio power coders equally well—thereby removing a fundamental objection to the question of whether such a language can be designed at all. With this fundamental tension resolved, there is no reason not to construct different dialects for maximizing the access of user groups with diverse needs to Evolvix functionality. Simultaneously, this responds to the NIH Data and Informatics Working Group's call for naming support systems^72^ that address substantial and ongoing globally distributed naming challenges, where biologists face the problems highlighted in Figure 1B over the time span of years (e.g., recent Ebola outbreaks^41^), decades (e.g., Enzyme Classification numbers), or centuries (e.g., taxonomy). Other disciplines could easily benefit from naming support too.^2^

Since long‐term backward compatibility is essential for enabling biology students of future generations to build on the models developed by students today, one of the first Evolvix requirements is to find a way of measuring progress toward this goal such that it enables a balance between the following two extremes: (1) enforce strict control over the Evolvix standard and its implementation, only allowing for code that builds on concepts that are tried and tested. While this is great for stability, it creates so many constraints on a development environment that it frustrates most computational biologists, who are looking more for opportunities to innovate than for ways to lock down code. For them, a stimulating research environment is important and requires a flexibility not easily reconciled with the long‐term stability needed for Evolvix. However, they would probably use the opportunity to publish their code if this was made simple by an appropriate package and documentation infrastructure. (2) Allow innovations to flow freely to address the abovementioned problem. This replaces one problem with another, as the path of innovation is not predictable. If all innovative ideas worth testing also have to become stable software packages for the long term, then the resulting inessential complexity^71^ will quickly destroy the system (and has done so for many legacy languages). Simplicity is to be highly valued in a standard. Designing Evolvix for simplicity is as much about what is not included as about what is. Thus, innovations should be easy to propose and easy to thoroughly review to keep the core of Evolvix simple.

To this end, we have been developing the StabilizingZone in the POST system (see online) using the BEST Names approach. It provides a framework for moving source code from less stable levels (MockupModel, NewNonfunctional, OperatesOften, PreProbing, and QualityQuest) gradually toward more stable levels (ReviewedRelease, StableSource, and TrustedTested). The latter are reserved for tools that have been (1) thoroughly reviewed, (2) tested by long‐term production use, and (3) are backed by mature scientific theories (see Summarizing Names in Supporting Information for details of this design.)

**Figure 4:**
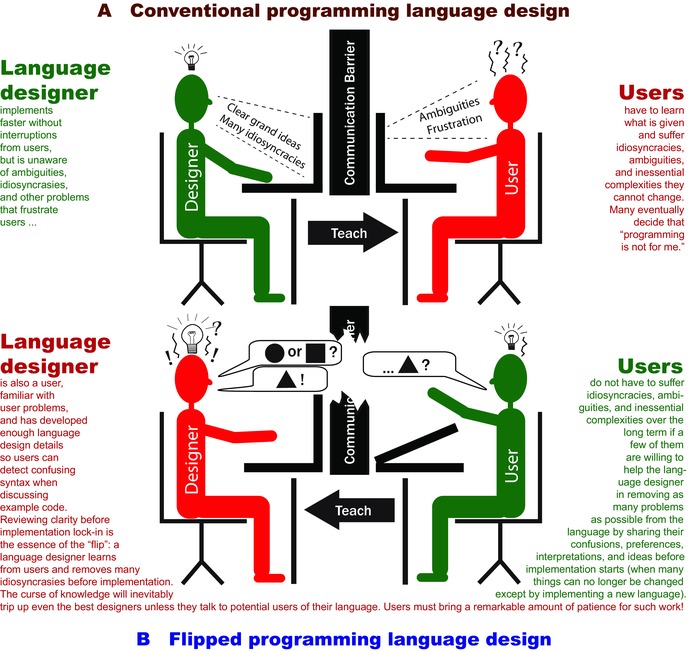
The Flipped Programming Language Design approach. (A) Most programming languages are designed by advanced programmers aiming to solve important types of problems in a better way; usually implementation is urgent and capabilities allow coding to start early. Few take the time to collect enough user and expert feedback during language design to break the curses of knowledge and ignorance. These curses make designers oblivious to idiosyncrasies and important missing features that frustrate both beginners and experts using the language. Many changes can be added after implementation starts, but fundamental redesigns are often prohibitively costly (e.g., Fig. 1A, changing names of logic operators). (B) Flipped Programming Language Design turns the tables in important ways by putting language designers in the hot seat (red) tasked with minimizing previously unnoted problems in language proposals as highlighted by users. Delayed implementation allows fundamental redesigns where needed. As an example from a different field, consider the 1940 Tacoma Narrows Bridge. Designs could have been changed before implementation, if more would have been known about “exceptional winds.” After construction, nothing could be done until it collapsed (see film at https://archive.org/details/SF121). It is often more difficult for a language designer to anticipate how a programming language for biology will be misread or fail than to solve such problems (as judged from several dozen redesigns of Evolvix); thus, repeated rounds of rigorous review by users and experts are critical. Not all words and concepts in a language need maximal scrutiny, but basic concepts and operators certainly do.

When developing with BEST Names for the long term, discipline and review are essential to avoid quickly cluttering namespaces with *ad hoc* names by misappropriating good names for general concepts to define much narrower terms (making them unavailable for a backward‐compatible unambiguous standard). While name clashes are difficult to produce with long names (which are also difficult to spell right), competition for Brief Names is fierce and requires careful balancing. To enable such long‐term developments for Evolvix keywords, all letter sequences that start with a whitespace have been reserved as such—following the lead of LLVM's argument about enabling long‐term growth of keywords (http://llvm.org/docs/LangRef.html#identifiers). Thus, Evolvix breaks with the programming language tradition of reserving keywords and instead reserves selected punctuation prefixes. Given the impact of such a requirement on users, we used the Flipped Programming Language Design approach (Fig. 4) to find the ideal symbol. This approach proved itself by ruling out tempting, but visually complex, alternatives before settling on a simple, unobtrusive symbol that is already widely used (e.g., in domain names): the dot. The only difference is that it directly prefaces all user defined item names in Evolvix without exception. It enables the use of BEST Names for keywords in Evolvix.

This also provides a clean slate for renaming everything in a programming language and lifts discussions with users to a new level about what might be useful to them. This new perspective motivated us to develop the Flipped Programming Language Design approach (Fig. 4) capturing many of the important experiences and values of our design process, and ensuring that development of the Evolvix architecture is driven by its mission to make accurate modeling easy. Approaches exist for engaging users, (e.g., Agile programming and use‐case driven design) and inviting user input to language development (e.g., Python PEP proposals, or regular user meetings held by the Titanium Project^73^).

BEST Names provide a powerful tool for incorporating user feedback, especially when combined with Flipped Programming Language Design to enable a much better integration of feedback from multiple rounds of reviews by users across disciplines and levels of expertise. Candid reviews (formal or not) can balance the language triangle of keeping simplicity, increasing expressivity, and reproducing the semantics of today's code for future researchers. We developed naming forms to help identify and assess candidate keywords for such long‐term use in code (see online Supporting Information).

Poorly chosen names are a big problem in biology, whether misleadingly labeling keywords and variables in programs or test tubes in the laboratory.^5^ But while programmers perfect their logic skills and biology students hone their experimental techniques, students in the humanities invest their time in developing the craft of clear and graceful writing. Attempting to combine all three with learning statistics and other essential modeling skills for simulating biological pathways is overtaxing and inefficient. This approach might be compared to starting a journey by beginning to assemble the necessary car from scratch. Such requirements will limit modeling quality. The division of labor in constructing cars may be analogous to a separation of concerns that could greatly improve the quality and use of quantitative models. Ideally, biological modeling is mostly about biology, just as driving cars is mostly about the journey and not about building the car (see online Supporting Information).

Our experiences with naming suggest that editors with a clear grip on the semantics of English have much to contribute to the clarity of programming languages designed for a broad audience (see online Supporting Information). Just as the Plain Writing Act of 2010 (http://www.plainlanguage.gov/) helps to guide word choices to simplify the communication of complex laws and regulations, a programming language with a well‐crafted set of BEST Names can make computational biology more accessible. Our domain expertise in navigating the language triangle and the opportunity to design general‐purpose programming capabilities (Table 2) for Evolvix from scratch provide a unique opportunity. Applying the Flipped Programming Language Design approach may result in the first general‐purpose programming language designed by biologists for biologists facing biology's complexities. It may even be useful elsewhere, as everything can be added later to a programming language, except simplicity.

## Conflicts of interest

The authors declare no conflicts of interest.

## Supporting information


Social contracts about cars and computersNaming is a hard problem in scienceCommon naming problems in programming and modelingBlacklisting confusing keywords in simulations of biologyUniquified names by versioning or by hashingPerspectives on naming from the humanitiesOnline referencesMini survey on improving namesNaming forms: debugging tools for Code2Brain interfacesA Project Organization Stabilizing Tool (POST) system for evolving order and stability from innovation in chaotic environments

## Footnotes

1 BEST Names were first presented in 2014, contributing to discussions on reproducibility,^3^ and then in 2016 at the “Workshop on Data Science, Learning, and Applications to Biomedical and Health Sciences,” held on January 7–8, 2016 at the New York Academy of Sciences (https://sites.google.com/site/dslabhs2016/proceeding-papers).^30^ We have been developing and using BEST Names throughout that time. Our work in developing Evolvix as a user‐friendly model description language with a focus on biology spans algorithm development and simulation work aiming for a deeper understanding of specific biological systems.^13^, ^31^ BEST Names have been inspired by, benefitted from, and served well in the overall mission of Evolvix to make accurate modeling more accessible.

2 For example, in mathematics, it has been observed that the “number of systems of terminology presently used in graph theory is equal, to a close approximation, to the number of graph theorists.”^74^ This might stem from an emphasis on axioms and formal results, requiring a clear view of the structure of operations; naming is comparatively less important. A mathematician illustrates this way of thinking by pointing out that from a strictly logical point of view, it would not matter if “points” and “lines” were called “frogs” and “kumquats” (see p. 397 in Ref. 75). Thus, to maximally reduce clutter from naming, many mathematicians prefer single letter variables that maximize brevity at the expense of readability for nonexperts. The resulting multiplication of synonyms, near synonyms, and homographs leads to steeper learning curves for beginners and extra work for experts comparing results. BEST Names could reduce costs of names in mathematics just as in biology.
