# Supplementary material for: Evolvix BEST Names for semantic reproducibility across code2brain interfaces
Source: Ann N Y Acad Sci. 2016 Dec 5;1387(1):124–44. doi: 10.1111/nyas.13192 (PMC5299481; doi:10.1111/nyas.13192)
Supplement: Supplementary file 1 — Social contracts about cars and computersNaming is a hard problem in scienceCommon naming problems in programming and modelingBlacklisting confusing keywords in simulations of biologyUniquified names by versioning or by hashingPerspectives on naming from the humanitiesOnline referencesMini survey on improving namesNaming forms: debugging tools for Code2Brain interfacesA Project Organization Stabilizing Tool (POST) system for evolving order and stability from innovation in chaotic environments [file NYAS-1387-124-s001.pdf]

Evolvix BEST Names for semantic reproducibility across code2brain interfaces

Loewe, Scheuer, Keel, et al. (2016-09-12)

Annals of the New York Academy of Sciences
http://dx.doi.org/10.1111/nyas.13192 ; updates see: http://evolvix.org/naming

Overview of online Supporting Material

		Page
1.	**Social contracts about cars and computers**	2
2.	**Naming is a hard problem in science**<br>Definitions of naming and more – 6 infinite naming challenges – 8 impact of naming on reproducibility – 9 two sides of one coin, merging – 11 life cycles of names – 12 C2B interfaces explained – 13 coding error classes – 14 defining semantic rot and semantic reproducibility – 17 cost of names	5
3.	**Common naming problems in programming and modeling**<br>Beginners vs experts – code vs math – compare implementations – merge models – 20 read foreign code bases – 21 dynamic names	19
4.	**Blacklisting confusing keywords in simulations of biology**	22
5.	**Uniquified names by versioning or by hashing**	23
6.	**Perspectives on naming from the humanities**	25
	1. Experiences with Flipped Programming Language Design of Evolvix	26
	2. Naming experiences with Evolvix Development	30
7.	**Online references**	31
8.	**Mini survey on improving names**	33
9.	**Naming forms: Debugging tools for Code2Brain interfaces**	42
10.	**Introducing Project Organization Stabilizing Tool (POST) system *RRv1***<br>***for evolving order and stability from innovation in chaotic environments***	49
	50 Preface - why is this interesting – 52 the problem – a solution – 54 introduction of ‘hello world’ example: using POST for writing a paper – 56 high-level structure – 58 how personalized medicine, cancer research, and evolutionary systems biology may benefit from POST – 59 area specific controlled vocabulary in POST – 63 defining and evaluating *TrustedTested* – 66 POST functional design requirements – 68 rules for automating and extending POST Home Folders – 69 Translation in POST – 70 current status – refs – 73 InfoFlow overview – 74 Dictionary of POST BEST Names	

1. Social contracts about cars and computers

Why are we allowed to drive such dangerous machines as cars if we individually do not understand them well enough to build or fix them? The answer comes in the form of implicit and indirect, yet powerful social contracts between the users of cars and the manufacturers of cars who developed the collective expertise on how to make these complicated machines easy to use for most humans (most of the time). While we still need to learn about brakes and steering wheels, we can ignore most technical details. Yet remarkably, knowing how to build a car is not enough for driving today. The collaborative use of many cars on shared roads has given rise to social conventions that are completely unnecessary from a purely automotive technological perspective. But these conventions are vital for the semantic reproducibility of coordination signals required for collaborative driving in concurrent traffic networks. Imagine it was difficult to recognize the right side of the road, whatever that may be.

These non-technical conventions are critical for efficiency as they facilitate fast decisions in life-or-death situations. Not all research is a life-or-death experience, but most research could be made substantially more efficient, by social contracts that settle "on which side of the road" to drive and thereby greatly reducing inessential complexity. While care is needed to ensure that such contracts do not inhibit the innovative forging of new paths that necessarily start as single tracks, it was demonstrated by the initial human genome project how much such coordination could contribute to efficiency in research.[1, 2]

The level of semantic reproducibility achieved by red traffic lights is impressive. Such reproducibility of interpretation would greatly increase the efficiency of computational modeling or any other programming, yet such clarity seems like a distant dream. The gap is exemplified by the "No Warranty" disclaimers in packages frequently used for biological analyses such as *Excel, Matlab* or *R*. Naïve readers might interpret these disclaimers to indicate that not even elementary operations like adding two numbers are guaranteed to work. Few non-technical users realize the extent to which this is true, as numerical limitations exhaust the precision of 64-bit floating point numbers before counting the bacteria in a few hundred humans (most CPUs cannot add efficiently a single cell to a count of $2^{52} \approx 10^{16}$ cells, since rounding errors annihilate the addition of numbers that differ too much in absolute value. It is as if computers are silently running out of names for such numbers.

The real problem is not the existence of such limits, but rather the absence of warnings that can make it prohibitively difficult to check if said problems actually occurred or not. Addressing the problem of how to calculate correctly in light of numerical limitations is not trivial, [3-12] the subject of international standards[13-16] and essential for reproducibility of computational results in biology.[17]

The absence of warnings about ambiguous code that is potentially misleading also creates many unexpected problems in another area of importance for calculations: operator precedence. As seen in the example in Figure Main1A (see main text; when calculating "-3^2")

neither *R* nor *Excel* in their latest versions[1] warn about the potential ambiguity of the “-“ character (“hyphen”), which could either denote a negative number or a subtraction operator; the interpreters of arithmetic expressions in both *Excel* and *R* seem to assume that users have determined the correct interpretation in independent empirical tests or read the manual (which turned out to be more complicated than anticipated in the case of *Excel* – thanks to naming problems; see footnote below).

Discussing such details is – in the words of the car analogy – like diagnosing a highly technical problem with how to control the motor; we have done this here, not because this example is of particular importance, but rather to illustrate the broader point that computational biology is unlikely to become as efficient as it could be, if such low-level problems keep disrupting biological trains of thought. Unfortunately, numerous similar problems exist for the many programming languages with non-trivial rules for determining the priority of binary operations in complicated arithmetic expressions; even seasoned programmers are easily confused by these rules.[18, 19] Creating a syntax that is convenient to use and difficult to misread is a surprisingly hard challenge,[19] yet of high importance for a language that aims to simplify accurate modeling in biology as few biologists have the substantial training necessary for dealing with these

---

[1] This observation was replicated on a number of recent versions of R and Excel, the last ones of which were: *R* version 3.2.4 (as of 2016-03-10) and *Excel* 2016 version 15.25.1 (as of 2016-08-26 with all preferences for error checking switched on, including “Show Errors For …” indicating “Formulas that result in an error” [sic], while the default option “Turn on background error checking” remained chosen). Here are the empirical results:

Excel: entering = -3^2 yields ➔ 9 ; yet entering = 0 -3^2 yields ➔ -9 ; at least one of these is surprising.
R: entering = -3^2 yields ➔ -9 ; entering = 0 -3^2 yields ➔ -9 ; no surprise.

Searching (below) showed the following operator precedence rules relevant for our example (from higher ➔ lower):

Excel: … ➔ - negation (as in -1) ➔…➔ ^ exponentiation ➔…➔ + – addition and subtraction ➔…
R: … ➔ ^ exponentiation (right to left) ➔ – + unary minus, plus ➔…➔ + – (binary) add, subtract ➔…

These differences in operator precedence explain the results: Excel apparently reads the first “-“ as “negative number indicator”, and the second “-“as “subtraction”; It is not clear how R interprets “-“, as both interpretations, negative sign and binary operator, produce the same result given the operator precedence rules defined above. Moreover, the statement is mathematically ambiguous and would require parentheses for clarification, as whether it is (-3)^2 or -(3^2), is impossible to tell. We discuss these details to illustrate how many such small problems in programming languages can turn reproducibility into the big challenge that it is.

Naming challenges in documentation. The attempt to find the relevant documentation presented a rich source of complicated naming challenges of interest in itself to other parts of this study; it illustrates that names have to be readily accessible to users of these names, or else the use of such names turns from fast helpful indirection into an expensive detour, as in the case of Excel here.
Excel: Despite producing many hits, the built-in help of Excel 2016 for Mac did not produce relevant information on the problem described in an unrealistically long search time, as no appropriate search “names” (terms) were accessible. Results merely suggested that “subtracting in Excel is easy” (see help page “Add and subtract numbers in Excel 2016 for Mac”); local search terms used were: “arithmetic” (31 hits), “algebra” (10 hits), “subtraction“ (38 hits), “subtract” (78 hits), “minus” (57 hits), “operators” (30 hits), “precedence” (2 hits), “negative” (101 000 hits), or merely “-“(86 500 hits); in contrast, entering “Excel negative number vs minus operator precedence” in two independent web search engines quickly produced the following link: https://support.office.com/en-us/article/calculation-operators-and-precedence-48be406d-4975-4d31-b2b8-7af9e0e2878a which claimed on 2016-09-01: “Applies To Excel 2007”; a similar link for Excel 2016 could not be found in the given search time; thus we assume continuity of implementation when using Excel 2007 online documentation for inferring operator precedence in Excel 2016. While probably not unreasonable in this instance, reproducibility can be greatly complicated by separating implementation source code from documentation texts.
R: By comparison, it was easy to find the relevant information in the built-in help of R by entering either “help (“-“)” or “help(Arithmetic)”; several other “obvious” searches failed though (eg. “operator”, “precedence”, “minus”), indicating that searches in *R* also face naming challenges.

subtleties. These problems can be re-interpreted as a form of naming,[2] but are outside of the scope for this study.

Naming is simplifying for a purpose. If done well, it blends into the background to a degree that makes it almost unnoticeable, like the "names" referring to various abstract concepts that we learn as part of learning any human language. While human languages blend well into the background and enable us to focus the eye of our mind on a given topic, programming languages demand much more attention (usually drawn away from the research that inspired the programming). However, too much automation is counter-productive.

Oversimplified naming, which does not guarantee useful error messages when faced with ambiguities, will greatly exacerbate the challenges of computational biology. Such error messages are pivotal for convenient automatic name conversions are 'probably correct most of the time', but not always. If converters cannot guarantee that all potential problems are brought to the attention of users, then uncontrollable biases in data can only be avoided at great manual debugging cost. For example, a simultaneous data analysis by some of us (for another study) happened to report an experimentally observed numeric range recorded as **10-20** in an *Excel* spreadsheet; much to the surprise of the investigators it eventually read **Oct-20** (and none of us could recall being warned). Of course, *Excel* was not designed for our informal 'range syntax'; neither was it designed for storing gene numbers (a substantial fraction of which are "auto-converted" as well [20, 21]).

A fuel gauge of a working car that indicates a full tank is neither questioned nor manually checked. It also does not 'auto-convert' an empty tank into a full one in an attempt to spare drivers the inconvenience of having to stop to refuel the car; not even in 5% of the cases. Practical experience makes it is easy for us to see the need for such a car to be repaired.

As illustrated here, neither our general computational expertise nor the social contracts between developers and users have caught up with the subtleties of transforming data.

---

[2] It is possible to reinterpret the choice of 'operator syntax' as the problem of naming the formal details of a given operation, and 'numerics' as the search for efficient methods that determine a reasonably fitting explicit name (also known as floating point number) for a real number that can only be known by implicit names (such as arithmetic operations like 1/3, the direct exact result of which can never be stored in a binary number system). Numerical problems also represent the generic naming problem of including too many or too few elements (here: real numbers) in a set represented by a given name such as a single floating point number with rounding errors.

2 Naming is a hard problem in science

What is a name? In simple terms, *a name is a label on a box with content.*

Using a name means to access the content of its box.

Thus, a *name is also a specific search that leads to the content of said box*.

Knowing the 'right names' means knowing how to search for desired content.

Thus, naming is the construction of an indirection that creates a link, which leads from the name/label/search-details to its content. This view suggests an interesting conceptual similarity: a memory cache can be seen as an indirection between immediately accessible data (in the faster cache) and more distant data (in the slower box of memory). The former can be seen as a more readily accessible name for the latter. Thus, for two processors the problem of cache-invalidation becomes equivalent to the problem of two speakers who wish to detect whether both refer to the same content when using a given name. Hence, cache-invalidation and naming are indeed two sides of the same coin (see main text, Introduction).

Using a name. Accessing a name can imply great differences in how the content is affected in different situations, including referring to the notion of, making a copy of, getting the actual content (without a copy), traveling from elsewhere to get to the content, traveling through the box to other content (inside of the box or elsewhere), or trying to access the content of the box (but lacking permissions), deleting the content, reserving space for the content, or many other actions.

Definitions. While we treat ***names*** and ***labels*** as interchangeable here, ***identifiers*** might imply a broader meaning, which can include a *list of conditions* and *set memberships* that – taken together – can identify some desired content by specifying its ***type***. Thus, there may exist many, one, or no items of a given type. We might further distinguish identifiers from the ***identity*** of a particular ***item***, which could be taken to mean the whole of all ***sets and elements*** inside of this item as organized in a well-formed hierarchy (as defined by ZFC set theory [22], together with all interactions between these elements and the resulting traits that are relevant for this item (and may also include links to the environment). In this view, *identities can be very complex and might be identified by infinitely many identifiers or names*.
All such content referred to by a name always exists within a ***context*** which also has a name. If we want to refer to the content of a name without deciding whether it's content or context, then we call it an ***item***. Thus, each item is a set that exists in some context (ideally known by name), has some content (which may be represented explicitly or implicitly and for which the item provides a context), and belongs to a type (which defines all traits that are relevant for identifying this item); given this diversity, items can have many explicit and implicit names.

Naming an item, then, can be defined as the process of searching and finding an explicit or implicit label for some content that makes it easy to search and find again said content. Whether "finding" requires the construction of a new name or re-identifying an existing name, is immaterial to the observation that giving and using names are fundamentally similar processes, albeit running in opposite directions:

Naming: **given** (*a specified content*) ➔ **search & find** (*an appropriate name*)
Storing: **given a pair** (*name, content*) ➔ **find** (*a place to store and link both*)
Accessing: **given** (*a specified name*) ➔ **search & find** (*the corresponding content*)

Thus, using names requires methods for knowing or storing the names to be used (and a system for accessing the item referred to by the name). Naming becomes pointless when its name is more complex or more difficult to find than the item to be accessed. For example: when breathing we do not name individual molecules of air to check them out of their library, which has also been named ‘atmosphere’. In contrast, this very approach works well for managing books named by catalogue numbers. In theory, individual molecules of air could of course be named implicitly by specifying their coordinates in the space-time continuum; however, this only further illustrates that situations exist, where the cost of explicitly naming every item is both prohibitive and unnecessary as all items could be addressed implicitly. These considerations could be summarized by the old observation that humans give names only to who or what they value. See also discussion on *the cost of names* below.

2.1 Diverse perspectives on infinite naming challenges for communicators

Types of communicators. As stated above, names reflect some statement about importance that is related to the named item. Often, names serve the purpose of communication between communicators that can use the item. These communicators could be humans or animals with brains, or brainless computers or other machines capable of some information processing. Generally, there exist at least three perspectives on naming:

- The naming communicator connecting the name and the item
- The sending communicator using the name for any purpose
- The receiving communicator interpreting the name

For communication to be free of semantic rot (see below), all three communicators have to agree on a shared mapping between the name and its content, which serves as the definition of the name. For this to happen, the sending and receiving communicators have to learn the definition of the name from the naming communicator with perfect accuracy. It is important to note that this triangle of communicators exists irrespective of the nature of the communicators, which could include humans, computers, complex virtual machines, very simple look-up code, hard-wired tools, animals, plants, cells, signaling molecules, phosphorylation chains, and many other biological communication processes, as well as physical interactions between elementary particles or molecules (where communication is defined as interaction and the naming communicator would be the shared laws of physics).

There are, of course, many more communicators than those three that may share a name. Given the infinities of potential priorities for naming and the infinities of potential users for names (which reflect corresponding queries for content) it becomes clear that a great diversity of names can easily exist for any item. This is true even in a perfect world where all communicators would have perfect access to and understanding of the named item, perfect communication while naming, a perfect memory of agreed names (for all time), and perfect agreement about the priorities of naming. The latter implies a perfect understanding of the use

cases of names, their frequencies and costs, perfectly balancing all related trade-offs into perfect naming priorities for this item.

In an imperfect world, constraints on knowledge and time, differences of opinion, motivation, and many other factors, will create additional layers of infinities for any given naming problem.

Pure Names... Given these complexities, it is tempting to give up and use the first names that come to mind. Indeed, finding good names is a very hard problem. Recognizing this, Needham introduced the notion of "pure names" [23] which are characterized by the complete absence of any information that humans would consider to be useful: no hints about place, type, content, origin, destiny, preferences, usability, relations to anything, including anything memorable. These names are purely random-generated labels that can be quickly generated anywhere for any item as long as a high-quality source of randomness is available.

Such random names do not carry any meaning at all, except for their ability to serve as a label for the item they point to; thus they do not provide any hints that might help to remember or reconstruct them, nor do they provide help for finding where they point to. Such names are like islands that can only be found by those[3] who already know where they are.

… can collide. Their uniqueness depends on a very low probability for generating the same name twice by chance. This probability is usually not as low as one might naively suspect (see the birthday problem in hashing below); hence, pure names may need a substantial length if they are to provide a reasonable guarantee of uniqueness among the set of all elements that have ever received and will ever receive such pure names. Details depend on the use case and are ideally known before implementation, as its limitations are hard-coded by the length of supported hash values.

As soon as the probability of name collisions increases, such "pure" names can turn into a management nightmare if no precautions have been taken to handle the case, where two identical names are no longer unique, but instead (are expected to) point to two different boxes, each with its own content (the data structures known as hash-tables have efficient mechanisms for that). Below we give a Figure with collision probabilities due to the birthday problem to help choose a reasonable trade-off.

Classifying properties of names using four simple states. Table Main1 in the main text highlights many more aspects that turn naming into a challenge and provides questions that illustrate their impact on naming. These aspects can be organized in ways that highlight a certain potential property of a name, reflecting a some naming priority, aspect, or approach. It is possible to turn many if not all of these aspects of names into "pure essential properties" that define

- a corresponding well-specified "perfect" state of completion for the property,
- a catch-all for one or more complete opposites that totally lack the "perfect" property,
- a catch-all for the many intermediary states, where a bit of the property is present, and
- all other names to which this property is not applicable in any meaningful way.

[3] These are also called „name servers“ in a distributed computing context.

2.2 Challenges of naming and reproducibility in scientific computing

All names are imperfect … almost all the time. Reasons are easy to see when contrasting the uncountable infinitely many perspectives of how to evaluate infinitely many potential names with the short amount of time and other resources actually available for naming any item. Such imperfection usually means that someone will eventually want to find a better name. Thus, many, most, or all names are temporary on the medium, long, and very-long term, respectively. It is beyond the scope of this study to investigate the long-term limitations created by the fact that human languages change. Ignoring such changes, we are concerned with naming, because complex simulation systems critically depend on the reliability of their knowledge of certain chosen names that are vital for interacting with other systems. Thus any complex simulation system is as temporary as the definitions of the names it assumes to know.

… and limit reproducibility. This temporary nature implies that the stability and usability of names is intricately linked to the goal of making scientific computation reproducible and extendible in ways that do not degrade models to brittle bit-wise reproductions of previous results. Bitwise reproducibility is great, but most scientists would much prefer it to be supplemented by semantic reproducibility, which is defined as the availability of all metadata about every aspect of a computation that is necessary for the scientists, who have not performed the work, to interpret correctly the input, the output, and the individual steps of a computation. Such semantic reproducibility critically depends on the availability of names that reference items and or types that can be readily accessed for the purposes of interpreting the name. Whether these items or types are defined in source code or by conceptual references to real world entities does not really matter, as long as these reference links are not degraded.

Type information is critical for reproducibility. Within a compiler, data structures are frequently stored in a format that makes no sense in the absence of corresponding type information. In fact, the definition of classes in computer science usually creates a separation between the abstract type definition which is stored in one place and the definition of objects which are stored elsewhere in a compact format that does not repeat the information that is shared between all objects of the same class (and therefore stored only once with the definition of a class). Communication protocols, such as ASN.1, formalize this process when they transmit objects in the *Type-Length-Value* format, which only transmits the name of an object's type, the length of its content, and its actual content. Separating the content of items from their type information has long been used to improve the efficiency of communication, but critically depends on sharing the necessary type information reliably by other means. Much of this type information is an agreement on how to name various details of items, but it also includes crucial assumptions that are to be made, e.g. if certain values are transmitted, if nothing is transmitted, and whether there are limits to the range of values that might be allowed.

For communication to work a shared frame of interpretation must always exist.
If communication of specific shared type definitions is not possible or not practical, more general communication protocols can be used that build on a general type definition language that has been externalized before and thereby replaces more specific "shared agreements". However, even general standardized type systems, such as XML, JSON, or YAML have to be shared in advance by all communicators who do not wish to share more detailed information about the type of content they wish to communicate.

Importance of automated versioning. When developing software, it is unfortunately very easy to drop the seemingly mundane detail of precise version information of the code that defines the corresponding type of names if versioning is done manually. Tracking such details requires organizational and computational overheads that mostly translate into explicit or implicit naming complexity which implements a system that allows for the reliable deduction of type name version information among all communicating parties. It is costly to develop such automated versioning solutions for a new application; thus many developers ignore this problem until it is too late. However, such overheads can in principle be greatly reduced by delegating the tedious tracking work to an appropriately implemented compilers; while the cost of such an implementation will likely exceed the cost of any single ad hoc solution for a given application, it is easy to see, that moving such automated versioning tools into a compiler provides an excellent return on investment for the whole software ecosystem, as the corresponding capabilities are now ready to use for every developer of every app – “batteries included”. Implementing such ideas greatly benefits from the Flipped Programming Language Design approach chosen for Evolvix; such generalized versioning can quickly become unworkable or unnecessarily complex if a given compiler-language pair cannot adjust some fundamental design decisions that easily get in the way (such as not storing any versioning information).

Versioning by hash and dependency tree. This problem can be substantially simplified if there is a way of delineating unique complex sets of type definitions that change over time. For example, NIX is a functional programming language with delayed evaluation that removes all problems from incompatibilities between diverse software packages that demand conflicting dependencies [24]. It does so by renaming each version of each software package it installs by combining its usual package name with a long-unique hash value that essentially uniquifies the name. This allows many mutually incompatible versions to peacefully coexist in one system; newly installed packages are then redirected to the corresponding assigned unique names for the right version of the packages they need. Thus, different incompatible versions that create great headaches on other systems can peacefully coexist if installed by NIX. This approach is powerful enough to run NIXOS [24], which is a Linux version that is entirely managed by NIX. Thus, seemingly simple and slightly cumbersome renaming exercise can prevent uncounted hours of debugging nightmares in what is otherwise known as “dependency hell”.

2.3 Naming and cache invalidation are two sides of the same coin

Shared name definitions and caches. The above need for definitions shared among independent communicators that each have their own localized storage creates a deep connection between the problem of naming and the problem of cache invalidation: if the meaning of names is allowed to change, such as when a type definition is unilaterally changed, then how is the other party supposed to know that this meaning changed, if the last copy that defined that meaning is available but not clearly recognizable as no longer valid? If there was a naming scheme that would uniquely identify every version of a type that ever existed, by a name that is guaranteed to be unique, and every communication including content of that type was to also communicate the precise unique name of that type (and its version), then the recipient communicator can compare the received type name with the locally stored type name and determine whether an update of the local type definition is necessary for interpreting the content

or not. Updating a type definition by downloading (for computers) or asking (among humans) is not difficult in principle, albeit creating a small slowdown. However, not know whether a given type definition is actually applicable is a much more complicated decision problem.

Merge conflicts in Git. Thus, content that comes with a correctly controlled version number that references a required type for interpreting itself can easily be used for invalidating a local version that has a different version number. This can be used to highlight “merge conflicts”, where the computer cannot easily predict correctly and unambiguously, which version is the latest revision that includes the latest changes; the distributed version control system “Git” [25] has is very good at bringing such merge conflicts to the attention of users and demands a “simple” naming decision that identifies one of two conflicting files as “more recent” and the other as “outdated” (this is indeed simple for a handful of files that were all recently created, but that is not a case where Git is needed or used; much of the frustration developers experience with git comes from the challenging nature of performing this “simple” naming tasks for large sets of changes).

Auto-merged loss of data in the cloud. The same problem of tracking names and content exists for all distributed computing systems, including storage in the cloud. Yet not all systems have naming conventions or other mechanisms for safely handling all cases. An example is given in the main text (Fig. Main1B) where Alice keeps editing a local document that is automatically synced via cloud to Bob’s computer. As soon as Alice has finished editing, she tells Bob in real time that she’s done. Bob, assuming “the cloud works”, starts editing his local copy of Alice’s document right away, but does not realize that he actually works with an older version and does not contain the latest important edits from Alice. If Bob continues to work on a file with the same name, either Alice’s or Bob’s changes are likely to be lost (and if not require merging in a painstaking manual process). An equivalent scenario was experienced by some of the authors when using a major “cloud” that was accidentally observed to create such problems (and also make them again disappear without a trace, deleting Alice’s edits along the way; observing this required making local copies of the files that were affected). To circumvent these problems, the following very simple shared naming convention was developed. For example:

If all agree on the shared naming convention that …

All changing files end with suffix anyfile_v1r0p1_XY–**Busy** indicate

- only user XY is allowed to change the file (“busy editing this file”)
- everybody else is allowed to copy and read the file, but must never add changes.
- the file unchangeably becomes version variant: “version 1 release 0 patch 1”

All static files end with suffix anyfile_ v1r0p2_XY–**Sent** indicate

- user XY has completed all changes of the file and submitted it (“sent to others”);
- changing from “Busy” to “Sent” also creates a new unchangeabe version variant
- nobody (including owner XY) is allowed to change this file, making it immutable;
- everybody can make a copy of the file and change the suffix to “-Busy” after increasing the version variant number and including the initials of whoever intends to change the file (thus taking responsibility).

… then no data would be lost without the knowledge of users; open communication, a bit of synchronization and the commitment to always increase the version variant number (or an automatic time-stamp) greatly reduce the need for tedious manual merging, but cannot completely avoid it, say if by accident two persons edited the same text.

Usability. As much as this simple solution can solve the problem in principle, assuming perfect adherence to this policy might be challenging in groups that are less than well-trained and highly disciplined. Thus, creating the necessary shared name-space for human users requires much effort, which could be greatly reduced by a labeled transition system that implements a corresponding finite state machine for the states in which any text-file could find itself.
This system would store all possible transitions to other valid states and support users by automatically following the specified policies for copying/naming/renaming, whenever a file is read or modified.

Semantic rot. This example illustrates also, how semantic rot works. Semantic rot first affects the file name (which no longer links to what Alice and Bob think it links to). This triggers more semantic rot either by dropping edits or by reducing the time that Bob or Alice can spend on fighting semantic rot elsewhere. Scenarios like these are becoming increasingly important, as applications migrate to and through the clouds of distributing computing.

The name of the coin. This section would be incomplete without a tell-tale episode illustrating how difficult it can be to draw conclusions about naming that are rather obvious with hindsight (or from a different perspective). We had been using the “two sides of a coin” metaphor for a very long time before one of us eventually realized the irony in our situation: If naming and cache-invalidation are really two sides of the same coin, representing the struggle to appropriately share the same reality among multiple interacting communicators, then “naming” and “cache-invalidation” are indeed two *names* for something so big, important, and challenging that it integrates the two biggest problems in computer science. The fact that we struggle to name this, even if we use the length of a Summarizing Name does reduce the real impact on our struggles to write reliable distributed computing code or to name the parts of biological systems consistently. While it is clearly beyond the scope of our study to attempt finding an appropriate single name, it is clear that different dialect and synonyms could greatly illuminate model building if such deep connections can be captured appropriately.

2.4 Life cycles of names

In order to avoid problems like those of Bob and Alice in distributing computer scenarios, a well-defined life cycle for names becomes critically important. Here is not the place to define this, so a brief sketch as to suffice. The life cycle of a name starts with the realization that a type is needed for referencing an item that is to be named. Both type and item require an implicit name in the form of a storage location before there is any name that can possibly become meaningful. Thus, questions of naming are related to questions of memory management, since the relative location in memory is equivalent to an implicit name that can always be given (even if most programming languages never allow accessing it). However, even if the content of a name did not require storage, the name itself still could.

The life cycle of names is intricately intertwined with the life cycles of any other bodies of information that are described as text and employ some form of code in which names matter. Such life cycles of sequences of characters include the combined changes of any program ever written, the full set of updates to any database ever compiled, the software development life cycle in general, the provenance of each entry in an ontology, as well as the life cycle of the ontology itself, the cyclic improvement of scientific models that introduce and drop names as needed, the revisions of legal laws, books, dictionaries, etc. and finally the evolution of language itself. It is often impossible to tell where one of these lifecycles stops and another one starts, as they form an intricate net of definitions that might also be captured in some form of ontology computing (as defined in Table Main2 of the main text).

2.5 Code2Brain interfaces

One way code. In Fig Main1A, of the main text an overview of C2B interfaces is given. Every time a human brain inspires the writing of code, the B2C interface translates the ideas in the brain into characters in a file that can be compiled. Computer science has excelled at developing methods of ensuring that the code written by humans is translated correctly into the binary code that a CPU can execute. This combination of B2C and C2C interfaces has powered much progress in computer sciences. However, this model works best if an isolated programmer writes the code and uses it only as long as the brain remembers the meaning of the various names and structures that are important for understanding code. On some occasions this can be a matter of weeks or months, on others it can be years or decades. The details depend on the complexity of the problem, the documentation of the code, the knowledge of the programmer, but to a very large degree also on the quality of the names that are used in the program source code for denoting variables, functions and other entities.

Language definitions. One might think that programming languages are orthogonal to this problem, because they are carefully designed and documented, much more so than most applications that are much more specialized. Thus one might expect little ambiguity about what a certain syntax means. This may be true for the designer of that language and everybody who knows the specification. However, this does not necessarily hold for all code written in that language. Why?

Writing code in a misleading language. Before a coder can write code, the coder needs to read other code that somewhat resembles fragments of the code to be written. Thus the coder needs to use her C2B interface for reading code and constructing a mental representation of the meaning of this code. The fragments of meaning implied in a programming language definition are organized by a programmer nto a sequence of expressions that she believes can solve the problems the code is written for. If the coder interprets (i.e. reads) code wrongly, then semantic rot at the C2B interface will have impacted that coder's ability to write reliable code. If this becomes a learned error, it will translate later into cascade of errors, as this misleading mental representation is repeatedly used to create code with corresponding errors of meaning with multiplicative effects on all users of the code.

Reading code in a misleading language. New developers who might join the effort or try to debug a program will also use their C2B interfaces to construct a mental picture of what the program is doing and why this might cause the intended results or not. This process can be greatly accelerated by compilers that automatically check for common errors by alerting programmers to the precise line and symbol that created the problem. However, errors not caught by the compiler will still need to be resolved by users. It can be useful to distinguish the severity of coding errors as discussed next.

2.6 Coding Error Classes

It is not reasonable to expect any non-trivial program to be free of errors from the start. As compiled by Dr. Ray Panko on the Human Error Website (see for more details and refs),

http://panko.shidler.hawaii.edu/HumanErr/Index.htm

the basic human error rate when it comes to typing or other mechanical activities is about 0.5% with some variability that depends on the precise activity. Experts can be substantially more accurate, but rarely reduce their error rates more than 10 fold compared to non-experts. Rates of logic errors are higher than those of mechanical errors. They are also more difficult to detect than mechanical errors. Yet by far the most difficult errors to detect are errors of omission.

Thus getting computer code to work correctly is always associated with substantial efforts that are summarized under the label “debugging”. Over the years a substantial arsenal of weapons has been accumulated by computer scientists in efforts to automatically detect as many “bugs” as possible automatically, when compilers analyze code in order to translate them into other code that can be executed more directly on a machine that operates at a lower level.

Programming errors can be organized in a hierarchy according to how difficult they are to find (and thus how much damage they can cause; difficulty increases with each point):

- Literal (insect) bugs: long gone are the days, where small crawling insects could cause computer malfunction by walking across sensitive electronic components.
- Known errors for which compilers create clear and accurate error messages. While these might frustrate beginners by standing in the way of a working program, experts often give the recommendation to have compilers report as many errors as possible; every error caught earlier does not have to be caught later at much greater cost.
- Errors that always crash a program at run time are next easiest.
- Errors that often crash at run time and produce obvious nonsense help by making their existence clear beyond the shadow of a doubt.
- Errors that rarely crash and usually produce full results, but not always can be very difficult to fix, because most of the time it is not clear that they exist at all and if they do, it is often not clear how to reproduce them.
- Errors that are rare can be very difficult to find on distributing computer systems, where no two runs of the program will result in exactly the same execution path, because of a great variety of diversifying circumstances.

- It can be useful to consider what randomly changed bits can do; some programming errors can cause computers to behave as if the crawling insects of old can again randomly make connections, or as if a cat walked over the keyboard, or as if obviously malicious input is being provided. Good programs catch these cases.
- The second to hardest errors to catch are errors in the logic of the program, because these require a deep understanding of the purpose of the program and its problem domain. These errors are often caused by omissions (what should be there, but isn't).
- The hardest errors to catch are the errors in the logic in the programming language because programmers assume that the language designers will have done a good job by selecting a logic that adequately represents the problem domain of the programming language.

Programmers can always program around the deficiencies of any Turing complete language; however, this requires solving the same problem, albeit without the support of a compiler infrastructure and the freedom to define an appropriate syntax. Figure Main1A illustrates a few examples where semantic rot is caused by poor naming of constructs in programming languages, that lead to ambiguities which are interpreted in one way by many and in another way by many others.

Even though *Excel* and *R* interpret -3^2 differently, neither produces an error message warning users that their assumptions (C2B) could be wrong. It may come as a surprise to many programmers that even a frequently used operator such as the logical "AND" can easily be misinterpreted as "UNION" in the context of the Venn diagrams so frequently used in biology, even if programmers meant to obtain the "INTERSECTION" of two sets.

2.7 Defining Semantic Rot and Semantic Reproducibility

Let us assume the total amount of meaning M_t in a message *M* can be quantified by a measure of semantic units (such as the number of correctly re-identified elements of a set or the percentage of bits of a message; the details do not matter here). Let us further consider the communication between an encoding communicator *E* that encodes and sends *M* to the decoding communicator *D*, that receives and decodes *M*. We are interested in defining the semantic reproducibility, Re, and the semantic rot, Rt, in this simple example. To keep it simple, we assume that *E* perfectly encodes and sends all intended meaning M_{tE}, and does not add anything unnecessary or confusing. We further assume complete and perfect transmission of M_{tE} to *D*, where upon decoding it will be represented as M_{tD}. Thus we can assume that any discrepancies between M_{tE} and M_{tD} are entirely caused by errors on the decoding side, which simplifies classification somewhat. Thus any encoding from E can result in:

- something D can decode correctly
- something D thinks it can decode but does it the wrong way
- something that causes D to start "decoding" messages that were never sent
- something that D cannot decode at all (but can skip)
- something that causes D to crash (unable to decode any further)

and more cases that all distill down to either missing true content (knowingly or unknowingly) or making up wrong content (assumed here to be done unknowingly). Thus, *D* will think it received

$$M_{tD,subjective} = M_{tE} - M_{tD,Known2Miss} + M_{tD,Unknown2MadeUp} \quad \text{(eq. 1)}$$

and is only aware of communication problems on the order of

$$M_{tD,Known2Miss}$$

that grossly underestimate the true communication problems, which also include those that *D* is unaware of ($M_{tD,Unknown2Miss}$, $M_{tD,Unknown2MadeUp}$). Thus, a careful comparison with *E* would reveal that *D* actually only received:

$$M_{tD,objective} = M_{tE} - M_{tD,Known2Miss} - M_{tD,Unknown2Miss} - M_{tD,Unknown2MadeUp} \quad \text{(eq. 2)}$$

Where $M_{tD,Unknown2MadeUp}$ is subtracted, as it replaces the resting and non-confusing silence sent from *E* (such as before or after sending) with confusing and draining misinformation. It is interesting to note that in most real-life circumstances,

$$M_{tD,objective} < M_{tD,subjective} \quad \text{(eq. 3)}$$

and that equality can only be approached under the ideal conditions, where *D* can somehow guarantee to catch and reverse all instances, where it would otherwise make something up and not know it, and catch and remedy all instances where it would otherwise miss something and not know it (assuming such a remedy is possible with *E* and eventually leads to success).

Now we can define the semantic reproducibility, R_e, of content as it is in the process of transmitting from *E* to *D*:

$$R_e = M_{tL} / M_{tS} \quad \text{(eq. 4)}$$

Similarly, we can define semantic rot, R_t, as any subtraction or addition of the original message:

$$R_t = 1 - R_e \quad \text{(eq. 5)}$$

For this to work, sender and receiver of *M* would of course have to agree on how to measure semantic content. Generally, any system X that can measure the semantics of another system Y has to be more expressive (i.e. be able to differentiate and capture more cases of interest) than the system that is being measured. Thus, Kurt Gödel's Incompleteness Theorem would apply here [26, 27]. This theorem states that it is impossible for a sufficiently expressive formal system *S* (that is, one capable of performing basic arithmetic operations) to assign a truth-value to each of the statements in *S*, nor is it possible for *S* to prove that its derivations will always be consistent. To accomplish these tasks, one needs a more powerful formal system *S**, whose own completeness and consistency also cannot be proven without referring to a yet more

powerful system. This means that the semantic reproducibility of a transmitted message cannot be proven by that very message itself, as any message that could complete such a proof would certainly have to be capable of elementary arithmetic and would thus constitute a mathematical system complex enough in Gödel's sense, for his Incompleteness Theorem to apply.

Therefore, it is much easier to transmit a message without semantic rot than to precisely quantify the amount of transmitted rot without severely limiting the semantics of the message, such that it is too simple for Gödel's theorem to apply to it.

Such greatly simplified messages are completely defined by their type systems, which live between two extremes: they are either guaranteed to be correct, which means they are so simple that they are approximately useless outside of extremely well-defined narrow use-cases; or they are powerful enough to be used for anything in principle, which makes them so complex that it is impossible to guarantee their correctness. Thus, quantifying the semantic reproducibility of scientific research would have to rely on a type system with a logic that very closely mirrors the physical world. How to construct such a system, which has to deal with ambiguity and vagueness in a rigorous way remains an open question in logic research [28]. In our own research, we have repeatedly encountered situations where the Boolean logic that allows only for true and false statements falls short of describing situations where the answer is precise, but represents a probability between true and false, or it is impossible to compute an answer on time, a case in which it would be inappropriate to apply Boolean logic.

These limitations of Boolean logic are likely to repeatedly trigger the need to work around their limits when constructing biological models where many statements are less than clear-cut; yet, the need for imprecision and non-applicability in biology does not reduce the utility and efficiency of Boolean logic for many cases where it applies. In Evolvix, these tensions are being resolved by developing the data type "**BioBinary**", which requires two bits for storing one of the four states of the "**OKScale**", which helps to better capture the many exceptions observed in biology, without artificially forcing them into inappropriate True or False statements that generate semantic rot by forcing all code to pretend a level of clarity that simply does not exist. Using the Brief, Explicit, and Summarizing Names for the Type OKScale, which will become a keyword in Evolvix (hence no leading dots):

```
OKS  OKScale                        OKScale_UsedFor_Enumerating_BasicAlternatives_of_BioBinary_Types
```

Similarly, each of the four alternative states (including some synonyms) are defined as:

```
OK   WorksOK  Working  Standing  Works_OK__UsedToSay__WorkingFilled_StandingReliably_FullCheck_All_True
OKO  SlowOKO  Slowing  Mediocre  Slow_OKO__UsedToSay__SlowsOrStarts_MediocreImmature_FullCheck_Has_Maybe
KO   StopsKO  Stopped  KnockOut  Stops_KO__UsedToSay__StoppedFailed_KnockOutNilWorks_FullCheck_All_False
MIS  MIStake  Mistake  Nonsense  MIS_take__UsedToSay__NotApplicable_NonsenseMistakes_PartCheck_CannotSay
```

Supporting these 4 states for each BioBinary in Evolvix may provide an opportunity for substantially reducing semantic rot in applications of scattered big data that substantially varies in its applicability.

Since closed-world systems like computer programs are well-defined, and naming presents the enormous challenges discussed above, it is possible to summarize a formally rigorous naming approach as follows:

Time spent in getting the naming balance is time spent writing documentation, which ideally should help outsiders without the current naming context to understand the meaning of the various short names that are not understandable outside of this particular type of context. Thus again, the definition of a type system (here for context) carries the information necessary for rigorously interpreting a set of names.

Experts of a local context who use names frequently tend to abbreviate them; outsiders new to the context usually prefer longer names with more information that helps them connect to semantics defined by the given context. This is the essence of communicating by pointing to the "thing", stating it does some "thing" which is clarified in a very specific context by pointing to another "thing". An equivalent email reply:

> *"please find my answers in the link https: //drive.google.com/ open?id=alcnekwejtb",*

was received as part of this study, and made perfect sense to the recipient at the time.

A more serious example closer to programming languages is given in Figure Main1A. The statement, Set A is Set B AND Set C is surprisingly ambiguous, given how many people consider it to be unambiguous (hard core logicians will recognize the logical "and" in a Venn diagram and will conclude that "A" is the intersection; many other persons, when upon reading B AND C imagine areas and convince themselves that both areas are included, hence claiming that "A" contains the union. This has contributed to the resolve to rename logic operators in Evolvix in order to increase the clarity of the language for non-programmers (for fragments of this work in progress, see Section 6.1 on pages 26-27).

2.8 The cost of names

Explicit and implicit names have fundamentally different cost structures. For implicit names, the cost of naming is non-existent because items already have implicit names that are ready to use and defined by a specific type context (array indexes, geolocation coordinates and more). However, the cost of interpreting such implicit names can easily become prohibitive if the cost of understanding the semantics of the context exceeds the time and processing capabilities of the communicator aiming to use those names without semantic rot.

For explicit names, the cost of naming in a way that avoids semantic rot can be prohibitive because the given time and information needed for naming is not sufficient for finding an appropriate name that is correctly interpreted by a very large number of communicators. This cost is only worth paying when very large numbers of communicators actually need to use a name correctly and the consequence of incorrect use are severe. However, this cost can be

enormously reduced by avoiding the need to balance the many competing demands on a name [29], which have to reach a perfect tradeoff if the named item can only ever possess a single name. If, however, it is possible to give each item multiple names, and to add such names over time, then the cost of naming is greatly reduced: different aspects of importance can be captured by different names that point to the same item, even if they do not appear to be otherwise related. Also, names could be added as new communicators join the conversation, removing the need to anticipate all possible future users for names (or change names to fit future users by making them incomprehensible for past uses.) The BEST Names concept disentangles very large universal naming needs that would otherwise create impossibly difficult tradeoffs in very many naming scenarios.

It is impossible to calculate the real cost of naming and the semantic rot induced by bad names. Indeed, recent studies provide a clear indication that these costs are enormous, on the scale of billions of dollars every year for preclinical biomedical studies that are not reproducible [30]}

3. Common naming problems in programming and modeling

The concept of BEST Names has been developed by modelers to handle synonym overload in biology. It became clear that naming in the following use-cases also involved related trade-offs between longer, more readable names and shorter, more cryptic names.

3.1 Beginners vs Experts

Beginners and experts need convenient access to the same semantic units but have different preferences. Students like names with more information about the semantics of an identifier to reduce lookup time. Experts remember and abbreviate to reduce processing time, even if outsiders can no longer follow. Speed and readability are important, but can rarely be satisfied simultaneously by identifiers with a single name. A combination of parser and ‘pretty-printer’ could automatically translate between different dialects (assuming all names have been defined appropriately). This would allow readers to choose their own readability trade-off.

3.2 Code vs. Math

Names ideally follow the priorities and culture of the environment in which they are used. Thus, identifiers in source code should reflect at least some aspects of their semantics, even if abbreviated. Names in mathematical equations are usually reduced to single letters with subscripts. Abstracting such details away maximizes compactness and facilitates a quick grasp of the structure of mathematical expressions – at the cost of hiding most biological semantics in systems biology models. The resulting semantic irreproducibility can have severe consequences, when e.g. confusing indices in (n1 - n2) swaps hosts and parasites. Code in programs is often more explicit to facilitate debugging. Model analyzed in code and equations thus can add two manually managed ‘dialects’ per publication, which is inherently error prone and hinders reproducibility. The manual work involved could be much reduced by providing automated support (e.g. for detecting inconsistencies).

3.3 Compare different implementations of the same model

It can be desirable to have independent implementations of simulation models of the same biological system, yet building these often adds synonyms. In the absence of automated support, a precise comparison of the different names in these codes can cost substantial amounts of time for complex models.

3.4 Getting different models to interact with each other

It can be advantageous to combine independent models addressing different aspects of a larger problem. For example, different biochemical pathways in the same cell are usually studied by different researchers. To study interactions between these pathways, the models will need to be thoughtfully combined to ensure that different molecules get distinct names in the combined model, even if they had the same name in their original, more limited model (see the Amylase

example in Section 3). Conversely, identical molecules should get identical names, even if they were different originally (e.g. '`H2O`' and '`Water'`, each from a different model should map to a common identity). Adjusting identifiers manually in small models is easy, but lack of automation in large models becomes prohibitive quickly. These problems have stimulated the development of tools for extracting specific reactions from the Biomodels database to reuse them as bricks in larger, combined models (e.g. [31]). Wrestling with this combination problem has also revealed a fundamental difficulty with applying the concept of 'modularity' as currently understood in software engineering to 'modularity' in living cells [32]. Software modules usually seek to hide details behind small, elegant interfaces, which are then said to become implementation independent ('black-box modules'). Cells operate in the opposite way. They are made of 'white-box modules' that all share one name space (with varying degrees of probability as mediated by spatial structure in cells). Since every molecule in a cell can in principle interact with every other molecule in the same cell, Neal et al. [32] developed the "semantics-based adaptable interface modularity" (SAIM) approach to building white-box models. It recommends that entities carry their semantics in their name. This can be a daunting proposition if the semantics becomes more intricate, or it can limit the precision of models if no appropriate measures exist for dealing with 'imprecise names'. Many formal systems are Turing complete, and a system for describing 'the' semantics of entities in names is likely to be either very limited or Turing complete (i.e. can theoretically express any computation ever performed if given infinite resources). While it is unclear where the limits of white-boxing are, Neal et al. [32] did computational biology a service by highlighting that white-box code-level coupling can bring all elements into the same namespace without fear of confusion, since all elements have been properly named, so that only those elements interact, which are supposed to [32]. Such semantics based interfaces require careful management and automatic support to become reliable in light of human typo error rates [32, 33]. Installing and maintaining these tools might be too involved for ad-hoc use.

3.5 Reading and documenting a foreign code base

Much time in modeling is invested in trying to understanding models by reading the code of strangers (which may include the author of said code after a year) hence, no quick help from friendly colleagues. Venturing into a foreign code base is like a research expedition unto itself. It ought to be possible to record notes of key findings (e.g. on the meaning of x). Ideally such findings go where they were found and are likely to be used again. To translate names of variables, functions and classes from the naming idiosyncrasies of one programmer into those of another is a never ending thankless task. As a reviewer of this paper pointed out, everyone has their favorite naming conventions. We can confirm from experience, and paraphrase: everyone has a favorite coding dialect. And it is usually not one of the four identified in the Evolvix BEST Names concept; thus, a proper implementation of BEST Names must facilitate the creation and management of any new dialects. Languages, where compilers have learned to check for naming clashes and that can 'pretty-print-translate' code between any dialects, will stimulate new ad-hoc dialects that help navigate a new code base from a different perspective (guaranteeing integrity of the original code). Scientific software is often implemented in contexts that provide little time for documentation. Hence, tools that can facilitate repeated rounds of editing could make coding more pleasant, by making it easier to find good names.

3.6 Dynamic names are challenging

Situations where names change faster than processors can update their (cache) memory of them provide another way of showing why cache invalidation and naming are very closely related problems (see discussion above and in main text).

While it may seem that cache invalidation is only important for the memory of fast CPUs, it is equally important in research where

(i) many researchers can work for a long time before a new phenomenon becomes clear enough to give it a non-confusing name. This implies many confusing updates to local 'brain-caches' along the way.

(ii) The same discoveries can be made by different researchers independently, resulting in different names, which happens often in biology. This use of different names creates the equivalent of undetected invalid cache memory that generates much work for nomenclature and ontology committees who seek to reduce confusion from such invalid entries (e.g. names look different, but really point to the same thing underneath; or one name is a collection of things that need to be identified individually.)

The same dynamic unfolds in most non-trivial software development projects: code structures may need names long before their true purpose is clear enough for giving them an appropriate name; and different programmers may independently develop parallel structures. Little can be done to avoid these fundamental difficulties of naming in principle (yet efforts to take a few moments before naming a data structure to find a good name are surprisingly useful).

However, computers could make the human task **much** easier by offering their stellar memorizing, sorting, and comparing capabilities to help humans find the needles in the haystacks that cause problems. This is essentially what the integrated development environments of modern compilers do: automate and simplify code navigation.
Still, there is much room for improvement beyond providing simple graphical user interfaces or other well-known tools. The goal is to get computers to produce as much modeling code as possible automatically, without loss of accuracy, while providing a more efficient Code2Brain interface that preserves semantic reproducibility for all who attempt to work on a model.

4. Blacklisting confusing keywords in simulations of biology

It can take considerable effort to identify the confusion caused by some commonly used names in trans-disciplinary research environments and to identify alternatives. Hence, storing the results of such lessons learned might be worth the effort.

In Evolvix, the goal is to avoid computer science jargon words that have a clear meaning in biology that might reasonably be used in a model to describe actual biology. Avoiding such jargon can be difficult, since it is quite entrenched. However, this will be worth the effort, since it greatly simplifies discussions of Evolvix code across the domains of Code and Biology.

**Ambiguous Term**	**In Biology**	**In Computer Science**
inheritance	passing DNA to offspring	object-oriented coding concept
polymorphism	DNA variants in populations	mechanism to call functions
vector	carrier of gene or disease	data structure or linear algebra
root	helps plants store nutrients	starting point or a powerful user
tree	woody plant with a trunk or ancestral tree of descent	data structure
graph	shows data in a plot	data structure of a network
class	taxonomic unit	object-oriented coding concept

5. Uniquified names by versioning or by hashing

It is often desirable to quickly and systematically obtain names that are either guaranteed to be unique or that are unique with such a high probability that naming collisions can reasonably be assumed to not occur over the life-time of a given system; we call such names “uniquified”. Algorithms that produce such names are of particular interest for BEST Names implementations, as they provide the uniquified names required for defining Stable Meanings.

Versioning. To guarantee uniqueness, a versioning system can be employed that increments a counter whenever a new version needs to be uniquified. Such names can be small and the system can be fast; however, it also requires the central management of the version counter and hence a communication cost that can be prohibitive in distributed computing contexts.

Hashing. An alternative strategy based on hash functions does not depend on such central management, but in return does not come with a guarantee of uniqueness, and generates names of considerable size. Such instantly (most likely forever) unambiguous names can be generated from hashes computed for a unique content to be named using a hash function, provided the hash has sufficient length and randomness.

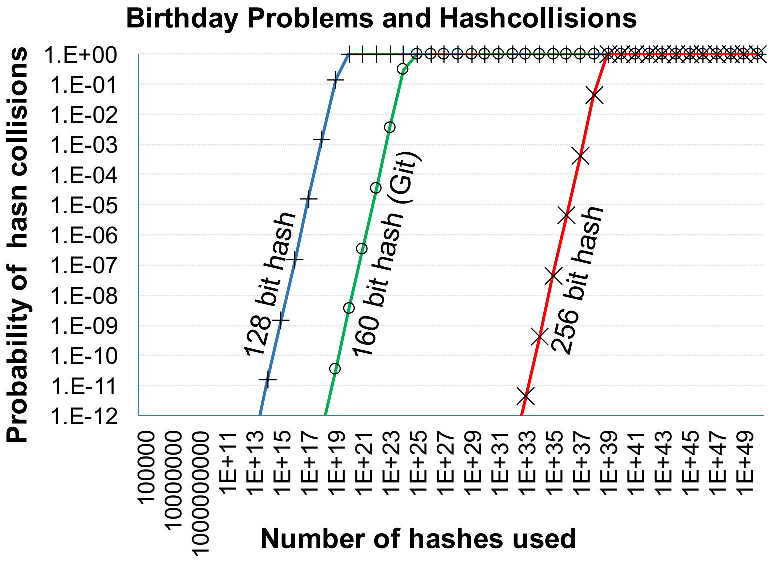

Figure S5-1: How likely are collisions between uniquified names?
The answer is predicted by the birthday problem, which gives the probability that any two or more persons from a group of given size share their birthday.

The length of a hash value and the number of hash values used will determines the probability with which randomly selected hashes will collide after the given large number of uses has occurred. The birthday problem is well understood under the assumption that the content and the hash function are both indeed perfectly random. While this is a reasonable initial assumption for most purposes if the hash-values are long enough, it is important to note that hash functions are still an active area of research, and deviations from perfectly random distributions usually increase the probability of collisions substantially. The event to avoid for uniquified names is a hash collision between any two or more randomly generated hashes that end up to be the same (and thus by definition are no longer unique). This is known as the “birthday problem”, from the observation that in any room it is much more likely that two persons have their birthday on the same day, than most would suspect.

The names computed by hash functions are either pure names (when the input to the hash function is completely independent from the content to be named), or their polar opposites, where all content of the box to be named is fed into the hash-function for generating a hash value that finger-prints the full content that is to be named.

In such systems, names can be used to lock in guaranteed content. They enable elegant data structures, such as block-chains and Merkle-trees.[34] These rather abstract sounding advances are critically important for implementations of many important applications, such as Git, peer2peer file-sharing networks, the ZFS file system, and many other systems that provide guarantees for the correctness of the content they store. Figure S5-1 illustrates some of the trade-offs involved in selecting hash functions.

Hash functions, collisions, and versioning are all active areas of research to be explored elsewhere.

6. Perspectives on naming from the humanities

The following two sections report on a rather unique project involving two humanities editors who employed their English expertise to improve the overall clarity of a programming language design for non-programming outsiders. Improving clarity is a key goal for the development of the Evolvix modeling language, which aims to greatly simplify the construction of biological models (and hence also programming) for biologists.

Please refer to the main text for a brief overview of "Flipped Programming Language Design," a process by which important names are rigorously reviewed repeatedly by developers and potential users before implementation of the programming infrastructure that makes a compiler work and simultaneously locks into place many of the big pillars of a language such as logic operators, elementary math, basic data structures and many other things that cannot or should not ever be changed for a language without changing its name. The following two texts have been written independently and have been edited only slightly to improve clarity for technical readers.

Summary: It is well worth listening to very diverse voices; cross-disciplinary work highlights different assumptions made in separate fields. The value of a clear grasp on English syntax and semantics is paramount for the semantic reproducibility of program code across Code2Brain Interfaces. Experiences like these suggest that a humanities education could have more value than might otherwise be assumed by a software industry that aims to produce stable, maintainable and understandable code. After all, the clarity of code is mediated by the clarity of names given in the code. What is true of software application code and software libraries is even more true for programming languages.

While many programming concepts require training in formal logic, semantics, syntax, and beyond, much of their formal elegance is lost when using them turns into an exercise in memorizing many syntactic rules and exceptions that are unnecessarily complicated due to arbitrary language implementation decisions or poor naming strategies. This accidental complexity may not matter for computer science students who work with computers all the time and are steeped in such exceptions. For those without such training, the concepts are difficult enough, but the inessential complexity [35] resulting from poorly chosen names and the resulting substantial increase in time to learn the basics means that many will not be able to learn the concepts, even if they otherwise could and actually would benefit from the use of a programming language in their research.

We do not need very many programming languages, just one that works as expected would be enough. Engaging editors from the humanities is not a silver bullet to create such a language if there was not already an idea for how to build one. However, they sure help avoid many of the semantic cliffs that are easy to miss when we trade clarity for speed. Maybe pair-programming is so effective because all code will have had to cross at least one more brain before it is committed.

6.1. Experiences with Flipped Programming Language Design in Evolvix Perspective of Editor 1

My background in humanities became very useful in unexpected ways after I joined the lab to support the development of Evolvix, a programming language being designed to maximize ease of use for intelligent non-programmers. I have since been highly engaged in the naming process for some parts of Evolvix, most notably renaming the well-known truth function operators used in Boolean logic and the development of what we called the Evolvix Stability Schema Scaffolding for supporting the organization of work and data in Evolvix (to better reflect its general usefulness, we renamed it as the POST Network while finalizing this paper, and it is described in the Supporting Online Material of this paper).

It should come as no surprise that my lack of training in computer science and advanced mathematics meant that some programming concepts required for discussing the syntax of a general model description programming language were difficult to understand.
Far more surprising, however, was the ease with which I was able to join the discussion and make important contributions. My ability to productively interact with the group resulted less from my formal training in historical research and more from my experiences trying to communicate history research results in accessible prose during a decade of study in the field. Three years of experience editing undergraduate papers as a teacher also helped hone my abilities to examine language in fine detail and guide others as they tried to clarify their ideas. The following example presents a single, early contribution that, I believe, enhanced the clarity of truth functions in Evolvix. While I have made many more contributions in a variety of ways, this one provides an easy-to-understand example that suggests the benefits of including trained writers in the language design process. Afterward, this essay concludes with sets of strengths and weaknesses that I see in the Flipped Programming Language Design Process.

As early as my first week in the lab, I began assessing the semantics of truth function operators as I interpreted them from the names presented to me by other members in the lab. While I understood such words as “AND” and “OR” in the common English usage, I had no prior experience using them as operators in a formal logic context. Before I joined the lab, our members had determined several semantic ambiguities in how logic operators were interpreted from the perspective of non-logic trained biology undergraduates. They had made significant progress in renaming them so that someone without training in programming or formal logic might understand them intuitively. These earlier renaming efforts facilitated my understanding of the concepts, as did the Venn diagram representations attached to each name. However, several of the then-current names still confused me, including the following:

OnlyUsual NotOnlyUsual

OnlyOther NotOnlyOther

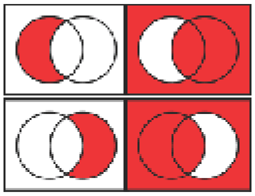

Here the terms “Usual” and “Other” represent input set 1 and input set 2, respectively. Lab members had previously determined that a good way to define operators that produced outputs that contained values strictly in the Usual or Other set would be to use the word “Only” (see left column above). To get an output of all values except those strictly in the Usual or Other set, we should only have to negate the ”Only” statement. However, this naming strategy seemed ambiguous to me: “Only” can function as an adjective and an adverb, which means it can modify either “Usual” or “Not.” Thus, while lab members interpreted the term as “Not(OnlyUsual)” – a negation of OnlyUsual – an inexperienced user could easily interpret the same name as “(NotOnly)Usual,” which could mean everything in the combined input sets:

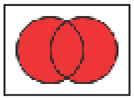

or even:

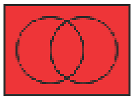

referring to everything *literally*, whether known or unknown. After some discussion, I suggested that the solution would be to find a short, commonly used word that could only be an adjective (and therefore only modify a noun) and that conveyed a sense of exclusivity. I suggested the idea of “Pure,” and a brief examination showed that it was a good solution because its denotation is “unmixed,” and it cannot be misconstrued as an adverb. This revision was done remarkably quickly despite the fact that the previous best solution had been stable for quite some time; the solution I brought as an outsider to the problem stands until today and looks as follows:

PureUsual NoPureUsual

PureOther NoPureOther

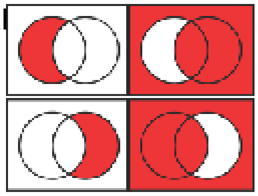

Note that, with the revised names, it is still possible to put brackets around the three-word name, but now the parentheses only make sense in the following context: “No(PureUsual).” If an unexperienced user were to mentally place parentheses around the first two words, “(NoPure)Usual,” the term becomes nonsensical because “NoPure” would indicate the absence of *any* pure output, whether Usual or Other, but the use of “Usual” still allows for an output of “PureOther,” creating a contradiction that is easy to catch intuitively. Thus, “(NoPure)Usual” is meaningless (as well as a non-intuitive reading of the word string), eliminating the ambiguity barrier that hindered comprehension of the earlier name strategy.

After eight months of participating in the Flipped Programming Language Design Process as I have experienced it in the development of Evolvix in our lab, I can now see the following strengths and weaknesses of the process.

Strengths:

1. **Collaborators from other branches of the Academy can provide fresh eyes.**

   Although my training was in history and cultural anthropology (neither focusing explicitly on topics like writing mechanics), the humanities' strong emphasis on effective writing for a broad audience provides significant training in linguistic form and style. Scientists and programmers may also be good writers, but many of them do not consider the production of English prose narratives as the ultimate product of research. Moreover, humanities students are trained to explore and analyze diverse worldviews in their research work, making it easier to forecast the needs and expectations of varied audiences. If programming languages are to become more intuitive and accessible for a broad audience in addition to becoming more efficient, then expanding the types of people who help create such languages will be *essential*.

2. **Programming concepts become more intuitive.**

   Many programming concepts require training in formal logic, semantics, syntax, and beyond. For those without such training in programming and computer sciences, the concepts can be difficult. Poor naming choices only add to the problem, leading to unnecessary complexities and lost time trying to map names to meanings; many will likely not bother to learn these programming concepts as a result, even if they could and might actually benefit from the use of a programming language in their research.
   I have found that many of the names developed in our lab have helped me understand difficult concepts better and more quickly, and several have been so useful that I have chosen to employ them as abbreviations and annotations in my day-to-day work.

3. **It is easier for non-programmers to contribute than many would expect.**

   While I needed explanations of various programming concepts in order to work effectively, my lack of experience in programming, mathematics, and other clearly relevant disciplines has not yet prevented me from making valuable contributions to the Evolvix language design. Since I bring an important skillset that complements (instead of overlaps with) the skills of other lab members, I have consistently been able to offer unique contributions to the naming and structuring activities under consideration in our lab. Thus I believe that effective human language communication skills are as important to programming language design as math and logic if a language is to be widely used and understood.

Weaknesses

1. **Expect more time explaining.**

   My lack of programming experience has helped me see things in ways a programmer would not, but it has also created a steep learning curve. When contributing to the design of a large, complex system, it is helpful to understand that system to the greatest extent possible. But I do not know how, for example, the algorithms at the core of Evolvix work, and I never will. The same could be said for many other parts of this language. As I have attempted to connect the dots during my work on various parts, I have consistently wanted to know how these parts fit into the whole; it is not always easy to "fly blind," and my lack of overview may have prevented me from suggesting even better solutions to various problems. This means that people with more complete knowledge must take time to explain to me what I am working on, a process that could also be seen as part of language review as I keep asking "why" in places where computer scientists might not. In a nutshell, the result tends to be slower progress but better results.

2. **No basis of comparison.**

   Because I have no idea what other programming languages look like, I have no concept of how our language compares to others – I am working in a vacuum. This can provide benefits (I don't internalize poor practices developed generations ago and adopted by virtually all programmers), but it also means that I have no benchmarks to measure progress against the strengths and weaknesses of other programming languages. It is therefore likely that some of my suggestions reinvent the wheel or are simply not practical, since they would conflict with too many important and well-chosen terms in computer science. Without programming knowledge, contributors wear blinders that may feel frustrating and inefficient, but are probably the inevitable consequence of bringing an outsider's perspective to anything.

3. **A few contributors from the humanities do not make a general public.**

   Because one of the key strengths of the humanities is exploring varied peoples, cultures, world-views, etc., humanities-trained contributors may feel as if they are asked to speak for the world. In the initial stages of language development, such contributors can provide much needed skill in conveying complex concepts and terms more simply, and they can offer insight into what diverse audiences need or expect if they are to use the language. But these efforts must be complemented with the opinions of a much larger sampling of the population; people trained to study and understand other people still have misconceptions, biases, and other limitations. The Flipped Programming Language Design Process will certainly not resolve all ambiguities in how outsiders read the code of programming languages just by running semantics past a small number of unorthodox contributors. However, I have become convinced that regularly listening to the perspectives of some pioneering outsiders will go a long way towards simplifying programming languages and ultimately also towards expanding the pool of contributors.

6.2. Naming experiences with Evolvix Development Perspective of Editor 2

When I was asked to help with providing names for Evolvix, I was immediately struck by the prosaic nature of the task. Naming? That must be the most basic and elemental of tasks associated with computer programming! Why such a fuss over it? But it occurred to me that it is also one of the most important and fundamental steps for anything that is created in this wide world of ours. Baby-naming is sacred in many cultures; indeed, anyone who has children knows how fraught with emotional meaning (both good and bad) this ritual can be. Families have shunned new parents for not favoring certain relatives with a namesake, and have also warmly welcomed back prior outcasts just for christening their firstborn in honor of great-grandfather Arnold. All I can say is, poor child.

Which brings me back to the task of naming for Evolvix. The idea of a “poor child” whose moniker is such a clunker that he will never be accepted on the playground is apt in this regard as well. It quickly became apparent at our naming meetings that it was a priority to avoid inadvertently choosing a scary sounding or confusing name. It is as hard as it sounds. First, the impulse in naming, at least in my case, is to always try to consider the audience for which you are naming. This audience was daunting in many ways: it included computer programmers and engineers, biologists and other scientists, as well as high-school students – all with very diverse experiences and expertise.

What names might qualify as a common denominator for virtually anyone without seeming to be too coarse-grained or simple to the point of losing any significant meaning? Second-guessing, normally a frustrating diversion in most processes, turned out to be a remarkably successful tactic in the naming process. Does it sound overly complicated? Is it misleadingly simple? Can anyone read this the wrong way? After finding a name we deemed to be a good candidate, we tested it by comparing it against a series of criteria that we had established as important in the corresponding naming process so far. Detecting ambiguity and ensuring words were used correctly was a top priority. Hilarious misuse of a word? Laugh and come up with another name.

There are some high-level concepts and algorithms in computer programming that do amazing things. One might think these actions deserve apt names that convey what they do in a breathtakingly elegant way. However, even programmers have found it very difficult to find such names, so many whimsical names are given instead. With fits of rarified humor woven throughout computational systems, it almost begs you to join in on the fun. Why not let our imaginations rip?

As an example, watch a young child play a sophisticated computer game some time. You’ll notice that she is certainly not aware of networks, arrays and code sequences, yet she is solving problems within the rules of the game. She is world-building. She creates life forms out of her imagination, depicting actions and features and giving them names.

Facing the complexities of rational naming, many programmers prefer to choose irrational, accidental or even randomized names to avoid the slowdown necessary for choosing good names. I can’t help but wonder if this task could be made easier by combining the intuitive, natural instincts of a child with the structure and sophisticated analysis of an adult, a process that I believe Evolvix helps facilitate.

7. Online references

1. Lander, E.S., L.M. Linton, B. Birren, *et al.* 2001. Initial sequencing and analysis of the human genome. *Nature*. **409**: 860-921.
2. Venter, J.C., M.D. Adams, E.W. Myers, *et al.* 2001. The sequence of the human genome. *Science*. **291**: 1304-1351.
3. Demmel, J. & H.D. Nguyen. 2015. Parallel Reproducible Summation. *IEEE Transactions on Computers*. **64**: 2060-2070.
4. Goldberg, D. 1991. What every computer scientist should know about floating-point arithmetic. March. http://www.validlab.com/goldberg/paper.pdf.
5. Kahan, W. 1997. Lecture Notes on the status of IEEE standard 754 for binary floating-point arithmetic. Accessed Oct 2004. http://www.cs.berkeley.edu/~wkahan/ieee754status/IEEE754.PDF.
6. Kornerup, P. & D.W. Matula. 2010. *Finite precision number systems and arithmetic*. Cambridge University Press. Cambridge ; New York.
7. Muller, J.M. 2010. *Handbook of floating-point arithmetic*. Birkhäuser. Boston.
8. Scott, L.R. 2011. *Numerical analysis*. Princeton University Press. Princeton, N.J.
9. Sauer, T. 2012. *Numerical analysis*. Pearson. Boston.
10. Verdonk, B., A. Cuyt & D. Verschaeren. 2001. A precision- and range-independent tool for testing floating-point arithmetic II: Conversions. *ACM Transactions on Mathematical Software*. **27**: 119-140.
11. Verdonk, B., A. Cuyt & D. Verschaeren. 2001. A precision- and range-independent tool for testing floating-point arithmetic I: Basic operations, square root, and remainder. *Acm Transactions on Mathematical Software*. **27**: 92-118.
12. McCartin, B.J. 1998. Seven deadly sins of numerical computation. *American Mathematical Monthly*. **105**: 929-941.
13. IEEE Computer Society. 2008. "IEEE 754-2008 IEEE Standard for Floating-Point Arithmetic". pp. 1-70. New York, NY: IEEE.
14. ISO. 2012. *Information technology - Language Independent Arithmetic - Part 1: Integer and floating point arithmetic (ISO/IEC 10967-1:2012(E))*. International Standards Organization. Geneva, CH. http://standards.iso.org/ittf/PubliclyAvailableStandards/c051317_ISO_IEC_10967-1_2012.zip.
15. ISO. 2001. *Information technology - Language Independent Arithmetic - Part 2: Elementary numerical functions (ISO/IEC 10967-2:2001(E))*. International Standards Organization. Geneva, CH. http://standards.iso.org/ittf/PubliclyAvailableStandards/c024427_ISO_IEC_10967-2_2001(E).zip.
16. ISO. 2006. *Information technology - Language Independent Arithmetic - Part 3: Complex integer and floating point arithmetic and complex elementary numerical functions (ISO/IEC 10967-3:2006(E))*. International Standards Organization. Geneva, CH. http://standards.iso.org/ittf/PubliclyAvailableStandards/c037994_ISO_IEC_10967-3_2006(E).zip.
17. Reed, M.B. 2011. *Core maths for the biosciences*. Oxford University Press. Oxford ; New York. Companion website: http://www.oxfordtextbooks.co.uk/orc/reed/.
18. Jones, D.M. 2006. Experimental data and scripts for Developer Beliefs about Binary Operator Precedence. *Last accessed 2016-08-29*. http://www.knosof.co.uk/cbook/accu06.html.
19. Razali, N., J. Noble & S. Marshall. 2015-10-26. Operators and Precedence in Programming Languages. *PLATEAU'15*. 53-56 (http://dx.doi.org/10.1145/2846680.2846690).

20. Zeeberg, B.R., J. Riss, D.W. Kane*, et al.* 2004. Mistaken identifiers: gene name errors can be introduced inadvertently when using Excel in bioinformatics. *BMC Bioinformatics*. **5**: 80.
21. Ziemann, M., Y. Eren & A. El-Osta. 2016. Gene name errors are widespread in the scientific literature. *Genome Biol*. **17**: 177.
22. Baumgartner, J.E., D.A. Martin, S. Shelah*, et al.* 1984. *Axiomatic set theory*. American Mathematical Society. Providence, R.I.
23. Needham, R.M. 1993. "Names". In *Distributed Systems (2nd ed.)*. S. Mullender, Ed.: 315-326. Reading, MA: Addison-Wesley (Pearson); a related talk by Needham: http://wwwhome.cs.utwente.nl/~sape/gos/chap12.pdf.
24. Dolstra, E., A. Löh & N. Pierron. 2014. NixOS: A Purely Functional Linux Distribution. . *J. Functional Programming*. **20**: 577-615 http://nixos.org/~eelco/pubs/nixos-jfp-final.pdf
25. Chacon, S. & B. Straub. 2014. *Pro Git*. Apress. Berkeley, CA. https://git-scm.com/book/en/v2.
26. Gödel, K. 1931. Über formal unentscheidbare Sätze der Principia Mathematica und verwandter Systeme, I. *Monatshefte für Mathematik und Physik*. **38**: 173–198.
27. Gödel, K. & M. Davis. 2006. The Incompleteness Theorem - Centenary Issue. *Notices of the AMS*. **53**.
28. Smith, N.J.J. 2008. *Vagueness and degrees of truth*. Oxford University Press. Oxford ; New York. Table of contents only http://www.loc.gov/catdir/toc/fy0906/2009278000.html.
29. Liblit, B., A. Begel & E. Sweetser. 2006. "Cognitive perspectives on the role of naming in computer programs". In *Proceedings of the 18th Annual Psychology of Programming Workshop (Proc. PPIG 18)*. P. Romero, Ed.: pp. 53-67, September 2006, University of Sussex, UK. http://www.ppig.org/sites/default/files/2006-PPIG-2018th-liblit.pdf.
30. Freedman, L.P., I.M. Cockburn & T.S. Simcoe. 2015. The Economics of Reproducibility in Preclinical Research. *PLoS Biol*. **13**: e1002165.
31. Neal, M.L., M. Galdzicki, J.T. Gallimore*, et al.* 2014. A C library for retrieving specific reactions from the BioModels database. *Bioinformatics*. **30**: 129-130.
32. Neal, M.L., M.T. Cooling, L.P. Smith*, et al.* 2014. A reappraisal of how to build modular, reusable models of biological systems. *PLoS Comput Biol*. **10**: e1003849.
33. Krause, F., J. Uhlendorf, T. Lubitz*, et al.* 2010. Annotation and merging of SBML models with semanticSBML. *Bioinformatics*. **26**: 421-422.
34. Knecht, M. & C.U. Nicola. 2013. A space- and time-efficient Implementationof the Merkle Tree Traversal Algorithm https://arxiv.org/pdf/1409.4081v1.pdf. *IMVS Fokus Report*. 35-39
35. Raymond, E.S. 2003. *The Art of Unix Programming*. Addison-Wesley (Pearson). Boston, MA, USA. http://www.catb.org/~esr/writings/taoup/.

8. Mini survey on improving names

There are many different ways to approach naming, which is the common task of finding a label that refers to a specified content in a given context. A person's approach to naming is influenced by a wide variety of factors, including personality, discipline, experience, life history, and other traits. These differences in background often cause people to have different priorities and outlooks on naming.

To further explore the diversity of naming approaches with a view on how to improve naming as a process, we solicited informal feedback among ourselves and from colleagues. We received a total of 32 responses reported below,

- 16 from persons who identify as "non-programmers" (10 "bio"; 6 "non-bio") and
- 16 from persons with substantial programming experience (6 "bio", 10 "non-bio"),

where among programmers and non-programmers, some persons had a background in biology (total 16 "bio") and some had not (total 16 "non-bio"). Their overall feedback is informally summarized in a paragraph below each question, and their more detailed responses are presented in aggregated bullet points beneath the summaries (each person could provide as many naming priorities as they desired to).

Conclusion: The variation in responses supports the notion that a system such as the BEST Names Dialects can facilitate naming by disentangling conflicting naming priorities, which surface, when different users of a common language meet in the same namespace and thus need to accommodate a variety of naming priorities.

Questions: Bold text below marks questions or text in the survey sent by email.
Summaries: Non-bold, non-italics below mark our overviews of responses received.
Responses: Italics below mark answers received and aggregated into similar groups.

In the questions below, the word "Aspect" was chosen to be a deliberately broadly defined property; interpret it any way you like, possibly including: precision, meaning, brevity, completeness, type-indication, classification, fun, formality, correctness, ease-of-use, memorability, speed of finding, speed of reading, speed of learning, adherence to a system, and/or many more ...

8.1. When you give something (or someone) a name, what aspects of the name do you consider to be important?

8.1.1 … In a general context?

The most frequent aspect desired by respondents is that a chosen name is not overly complex, remains jargon-free, short, and easy to pronounce and spell (13 respondents). Many respondents also indicated they desire names which are descriptive, reflecting at least some meaning (12 respondents). Others supported similar aspects, such as precise, unambiguous names (10 respondents) and names which are easy to learn and remember (7 respondents). A

smaller but significant number of respondents preferred name choices that are intuitive / rational (5 respondents); unique or “catchy” (5 respondents). Others valued an acceptable “sound” (3 respondents) or its history (3 respondents).

- *Not overly complex, either conceptually (e.g. jargon), or in actually length, pronunciation, spelling, etc. (13)*
- *Descriptive, implies at least some meaning (12)*
- *Precision, not ambiguous (10)*
- *Easy to remember/learn (7)*
- *Intuitive / rational name choice (5)*
- *Uniqueness, “catchiness” (5)*
- *Sound of the name (3)*
- *History of the name, any root words, prefixes, suffixes (3)*
- *Relevance to users of the name, intended use of name (2)*
- *Ability to distinguish this object from others (2)*
- *Avoiding possible misinterpretation, particularly for foreign speakers (1) or people from other fields (1)*
- *Not distractingly contrived or catchy (1)*
- *Shouldn’t go against previously established norms or language systems (1)*
- *General response to the name (1)*
- *Possible shortenings (e.g. initials) (1)*
- *Context-appropriate (1)*
- *Frequency of use (1)*
- *Make sure not copyrighted (1)*
- *Fun (1)*
- *Connotations (1)*
- *Whether the object already has a name (1)*
- *Subject type (1)*
- *Easier if name gives clues as to how relates to other relevant names (1)*
- *Hard to answer for general context (1)*

8.1.2 … In the context of computer programming (variables, functions, etc.)

For important aspects of names in the context of programming, preferences leaned heavily in favor of descriptive, easy-to-infer meanings (20 respondents; see also approachability, usability, ease of typing and remembering) and unambiguous, unique names (9 respondents). Many preferred short names (9 respondents). To some it seemed important that preferred names were, respectively, internally consistent (following any established project guidelines, 5 respondents), clearly defined right away (4 respondents), precise (4 respondents), and consistent with conventions (4 respondents). Several respondents hinted at what might be described as a need for building names by combination when requesting names that can be customized in order to become capable of being used in multiple contexts for different-but-related purposes (3 respondents; see also ‘able to use with modifiers’, and ‘creates patterns of names’). In addition to pointing to the general aspects above, these points were given:

- *Descriptive, easy to infer meaning (20)*
- *Not likely to be confused with another, not ambiguous, unique (9)*
- *Short (9), e.g. one letter with sub- or superscripts for math in case there is a need to write by hand (1)*
- *Internally consistent, follows any established project guidelines (5)*
- *Clearly defined right away (4)*
- *Precise (4)*
- *Consistent with current conventions and accepted technical terms, especially those related to programming (4)*
- *Customized for a given context or able to be used in multiple contexts for multiple (different but related) meanings (3)*
- *Able to be used with modifiers (paired with a variety of names) (1)*
- *Ability to be applied to a broader context to create a pattern of names (1), as simple as possible (3)*
- *Easy to remember (2)*
- *Easy to type and spell, easy to read as part of code (2)*
- *Approachability, e.g. will other people want to use it? (1)*
- *Understandable for people who are not domain experts (1)*
- *Not too many synonyms (1)*

8.2. Let's assume you learn about a name that someone else has given to something (or someone) and you can get all the information you want about this. What would you generally want to know and are there aspects that make learning about this name easier or harder for you?

8.2.1 … In a general context?

There were fewer points of agreement on this question compared to previous ones and 5 respondents found this question difficult to answer in a general context. Most wanted to know the basic meaning of the name or how it relates to its content (12 respondents). Fewer were interested in how the name linked to what they already knew (7 respondents), whether any synonyms and/or abbreviations existed (6 respondents), and what the name's history, original context, and root words were (6 respondents), or why this name was chosen (6 respondents). A number of diverse concerns were brought up by one or two respondents, such as pronunciation (2+1), additional examples (2), connotations, and various aspects that could be used to assess the quality of the name.

- *Want to know basic meaning, how name relates to object (12)*
- *Links to what is already known (7), mnemonics (1)*
- *Synonyms or abbreviations (6)*
- *Name's history, original context, root words, prefixes, suffixes (6)*
- *Why this name was chosen (6)*

- *Examples of things with this name, example of use in context (2)*
- *Precision (2)*
- *Pronunciation (2)*
- *Whether it sounds similar to a different thing (1)*
- *Associated with a context (1), but not so specific that it loses sense of the broader context (1)*
- *Connotations (1) or dependencies on certain contexts (1)*
- *Length (2)*
- *Intended audience / who will use the name (1)*
- *If the name is close to name of different (unrelated) concept (1)*
- *If the name seems to fit the thing being named (1)*
- *If it is simple but still unique (1)*
- *Links/citations/references on web (1)*
- *If it is well-documented (1)*
- *Consistency (1)*
- *Background of the person (s) who did the naming (1)*
- *Whether the name was hard to choose, and other choices considered (1)*
- *Easy to read (1)*
- *Not sure how to answer this (4)*
- *Hard to answer for general context (1)*

8.2.2 …In the context of computer programming (variables, functions, etc)

The largest point of agreement (10 respondents) was that names should clearly imply or describe known meanings that are easy to find. Some desired an example of how the names are used in context (6 respondents). Fewer responses preferred knowledge of why a name was chosen and which alternatives were considered (4 respondents), the types and domains of use (3 respondents), any similar names with potential for confusion 3 respondents), as well as various other aspects. Several pointed to the previous question, indicating a substantial overlap of naming in a general and a computational context (4 respondents).

- *Name clearly implies the described meaning; else desire to know that meaning, or wish to quickly and easily find the meaning (10)*
- *Examples of how used in context (6)*
- *Why this name was chosen, other alternatives considered (4)*
- *See point 2a above (4)*
- *Type and domain of use (3)*
- *Related variables, other names which could potentially be confused with this name (3)*
- *Specific, unique (2), harder with ambiguous or contradictory info on a name (1)*
- *Naming documentation (2)*
- *If fits with pattern, conventions (2)*
- *Links to what is already known (1), relevant previously published materials (1)*

- *Synonyms or similar names (2)*
- *Must be clearly defined (2)*
- *Easier if can relate to simple analogy (1)*
- *Easier to understand if already have background on thing being named (1)*
- *How the compiler "understands" the name (1)*
- *How name fits into broader context (1)*
- *Concise (1)*

8.3. Let's assume you read a new name (or word), but you don't get any other information. What aspects of that name make it easier or harder for you to understand what this name (or word) means?

8.3.1 … In a general context?

Most noted that it is harder to understand completely new words, and determining the meaning of such words is easier if they are available in dictionaries and published papers, or if words are constructed around recognizable roots or fragments that form parts of words (15 respondents). Nearly as many stated that they would use information about a word's context to help understand it (12 respondents). Many suggested that words are easier to understand if they sound like something common or already known, but only if the association is not misleading (10 respondents). Other responses noted that simple and descriptive, meaningful names help (combined 6 respondents), while abbreviations, jargon, context dependence, and the inclusion of numbers or personal names all make it more difficult to understand the name (combined 5 respondents). Examples and good documentation help (2 respondents).

- *Names are harder to understand if completely new; easier if can look up in dictionary (or previously published papers) or recognize root words or word parts or logical "clues" to meaning (15)*
- *Context helps (12)*
- *If it sounds like something already known or something common, assuming this association is not misleading (10)*
- *Simple (1) but still descriptive (3)*
- *Meaningful name (2)*
- *More difficult if an abbreviation (1)*
- *More difficult if jargon (1)*
- *Only one definition per name helps, especially if not commonly used (1)*
- *Harder to understand in multiple context-dependent meanings (1)*
- *Harder if name includes numbers or specific personal names (1)*
- *Examples or illustrations (1)*
- *Good documentation helps (1)*
- *Spelling, pronunciation (1)*
- *Sounds good (alliteration, consonance, etc.) (1)*
- *Hard to answer for general context (1)*

8.3.2 … In the context of computer programming (variables, functions, etc.)

The largest point of agreement was that consistently used contextual clues are key to understanding new words (11 respondents). Smaller numbers suggested descriptive words (4 respondents) and recognizable words associated with other known fields or recognizable languages, if the associations make sense (4 respondents).

- *Context, assuming used consistently (11)*
- *Descriptive words (4)*
- *Similar to other recognizable names, from other known fields or other recognizable languages, assuming these associations make sense (4)*
- *Root words, recognizable word parts (3)*
- *Variable type (2)*
- *Simple, short (2)*
- *Specific, clear meaning (2)*
- *Can understand why name was chosen (1)*
- *Easier if name related to meaning (1)*
- *Not jargon (1)*
- *Fits with analogy (1)*
- *Important if begins with underscore or capital letter (1)*
- *Follows conventions (1)*
- *Precise (1)*
- *Memorable (1)*
- *Not Greek letters (1)*
- *Able to be pronounced, i.e. not a symbol like "!" (1)*
- *See point 2b above (2)*

8.4. Are there situations, where you would prefer names, respectively, that are …

8.4.1 … longer?

A plurality of respondents favored longer names if the concept being named is very large, complex, or confusing (7 respondents), requiring longer names to remove ambiguity and be more descriptive (7 respondents), or uncommon situations required more explanations (combined 7 respondents). Justifying increases in length of names included improved understanding, readability, and the need to remember less; or if names included additional information like metadata, or modifiers, or as generated from the combination of meaningful parts (combined 7 responses). While a few felt that esthetic considerations could justify increasing name length, at least one respondent felt that longer names were under no situation preferable.

- *Concept being named is confusing, complex, larger action e.g. file hierarchies, functions (7)*
- *Need longer name to be precise or unambiguous, need to be descriptive (7)*
- *Not commonly used (3)*
- *Unfamiliar situations, or if named for new audiences or students (2)*
- *General ideas (1) , little to no context (1)*
- *Increase readability (1)*
- *Easier to understand (1)*
- *Don't need to remember or memorize as much (1)*
- *If compound name with meaningful parts (2)*
- *If longer because includes modifiers (1)*
- *Sometimes helpful to include metadata in names (1)*
- *Longer better if used by/for people who already have a relevant background to the thing being named (1)*
- *Baby names, to allow for later (shorter) nicknames (1)*
- *To make it sound nice (1)*
- *Very important objects, e.g. emergencies (1)*
- *Better for titles (1)*
- *Under no situation (1)*

8.4.2 … shorter?

Most respondents preferred shorter names all else being equal, but named different conditions for when shorter names would be acceptable. Some required maintaining precision (6 respondents), others that short names were only used for something simple, basic, or small tasks (5 respondents), or there be a particular need to read or write efficiently when used often, such as for variables in mathematical equations (combined 7 responses). Some thought that unique names did not need additional clarification and could be short (3 respondents), while others preferred shorter names in general (combined 4 respondents), or only locally (1 respondent). Some saw the potential for shorter names to simplify remembering, reading or communicating (3 combined respondents), and parallels to question 1b were noted.

- *Preferred to be short but still precise (6)*
- *Something simple, basic, smaller task (5)*
- *Increase efficiency (1), speed of writing/reading (2)*
- *Used often (2)*
- *Preferred for variables (2)*
- *Don't need additional clarification, unique name already (3)*
- *Generally preferred (2)*
- *Always prefer shorter names (2)*
- *Programming in local contexts (1)*
- *Abbreviation for a longer name don't want to have to remember (1)*

- *Easier to remember (1)*
- *Pet nick names (1)*
- *Must be clearly defined right away (1)*
- *Easy to communicate (1)*
- *Reading aloud (1)*
- *Domain expert (1)*
- *If it increases consistency or easier to read (1)*
- *Relating many different objects (1)*
- *See point 1b above (1)*

8.4.3 … other preferred properties?

Fewer respondents noted additional preferences. Some restated their preference for short names in general, and in particular for concepts related to math (3 respondents). Other single respondents emphasized the need for different names for different audiences or use cases; the need for additional explanations or a dictionary that maps short to long names, or might even adjust the balance between length and descriptiveness, or require longer names when needed to reduce ambiguity. The importance of a common naming scheme for related functions and variables was highlighted as well as paying attention to case to improve readability.

- *Prefer shorter names (2), especially for math (1)*
- *Common name for similar functions and variables (1)*
- *Technical names when need to be very precise or show expertise (1)*
- *General names when trying to collaborate with people from other fields or with general audience (1)*
- *Consistent internally and with standard conventions (1)*
- *Case is important for readability (1)*
- *Would like dictionary to map short to long names (1)*
- *Explanations for names (1)*
- *Longer names when need to reduce ambiguity (1)*
- *Balance between length / descriptiveness (1)*

8.5. Summary of additional comments from respondents

Further comments also indicated that the willingness of respondents to engage in discussions about naming varied greatly: from the recommendation to use *ad hoc* names or efficiently delegate this tedious work in order to avoid wasting too much time on it (at one extreme), to the willingness to invest time according to its importance, to actually enjoying the process as one that facilitates a deeper understanding of the item that is being named, and hence possibly stimulating new discoveries (someone described naming as “illuminating and fun”). Some noted that even though naming can be sometimes tedious, sticking with the process until a really good name can be found was important and worth their time. When exactly that endpoint was, however, was difficult to pinpoint according to some comments. Working together as a group on

naming can be rewarding, stimulate creativity and open up new horizons in the form of encountering new disciplines. It is also likely that collaborative naming can be substantially improved. For example, a larger group could be broken down into small independent groups that can quickly generate and evaluate new names and thus efficiently generate naming proposals for efficiently combining them in a quest to find better names. Survey responses suggested that this and other systematic process improvements might be able to substantially increase the speed at which different credible naming proposals can be made and evaluated.

Challenging aspects of naming that emerged, included its general complexity, the lack of knowing when exactly a name was “good enough”, the need to revisit names sometimes more than once until a good one could be found. Naming discussions are frustrating for people who want get quickly to using the thing being named and then find themselves stuck in a discussion on names (“there are more important problems”). One person remarked building a “tolerance” for naming discussions, while another thought that *ad hoc* names were usually good enough to convey the meaning necessary in a programming context. One person remarked on the ability of names to include or exclude, highlighting the great responsibility that comes with naming.

By and large, respondents with a math/computing background seemed mostly interested in limiting the time spend on naming and preferred by and large shorter names; Not everybody responded to the mini survey, and a noticeable majority of non-respondents had computational backgrounds. Hence the observable bimodality in the following mini histogram should be interpreted with much caution and may at best to be taken as an incidental observation in need of more systematic scrutiny.

If you could change the naming process anyway you wanted, what would you do?
(Please check with x *any* that apply or describe what you would suggest) It was allowed to choose more than one:

0x__ **1. "Don't ask me to work with others on naming:**
naming my things is my business, naming your things is your business"

2x__ **2 "I see that naming is necessary, but I hate doing it, so**
please do it for me and just tell me the conclusions"

10x_ **3 "While I don't enjoy this, I don't mind it either as long as it's not too much"**

6x__ **4 "If it is important, I could improve names for a long time"**

15x_ **5 "I like naming, because**
it makes me think about what is being named in a more precise way"

____ **6 ... (please add as many other statements as you like) produced:**

1x__ *"Naming is very important, therefore I would refine names jointly with domain experts till the names' meanings stick to my mind and to their minds. Only then, I'd stop refining and would start producing".*

How to use these Naming Forms

1. The goal of these forms is quality improvement for Naming, which requires evaluating different *Names* for use by diverse audiences. Different equivalent (i.e. strictly synonymous) *Names* are defined as

- **different ways of expressing** ("Syntax")
- **one precisely defined meaning** ("Semantics").

These forms were made only to improve the quality of names and naming processes, not for investigating human capabilities. They do not record what is needed for useful conclusions on the latter. The forms aim to uncover hints of how a multitude in a larger population might misunderstand the names investigated to motivate searches for better alternatives; such goals are incompatible with the study of human capabilities.

2. Why you should stay anonymous if you are a reviewer of names
It is of utmost importance for the success of *Naming*, that your evaluations of names are based on

- ***your actual understanding*** of these names without further help, as that is also how other people encounter such names in real life;
- **not on idealized knowledge** that you feel you "ought to know" or "could easily look up", nor
- **not someone else's likely knowledge**, and certainly not that of someone who is "trained".

The last reason is most distorting, as humans can learn ***any*** name with enough effort; that does not make it a good name, which needs to be interpreted correctly by many who have not seen that name before. Thus reviewing names requires an openness in communication style that can be very difficult to achieve for humans if any fear of judgment or other unintended consequences of speech lead to "self-censoring". Thus, please do stay anonymous and do speak your mind. If you say something unusual or highlight something you don't understand, you might speak for millions on a global scale: thus, please be precise.

3. Don't be intimidated by the many questions: Rather pick and choose as you see fit.
Skip what you can't speak to easily if you don't have time; even partial answers can be very useful.

4. If you need more space on a page, use the space below, then attach pages; always annotate clearly:

- **Page number**, then MeaningTag, YYYY_MM_DD Date , and ContributorTags.
- **Question number on a page**, to create a reference back for additional content on extra pages.

5. Please rate all traits of the given expressions on these scales
Check one of the vertical boxes at the extremes if the criterion is not applicable or you want to abstain from rating to strengthen the voice of other raters (by a "non-vote"; else choose some intermediate value):

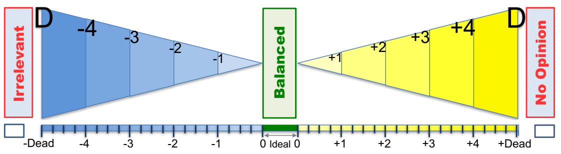

Balance Scale
How well is the given trade-off managed?

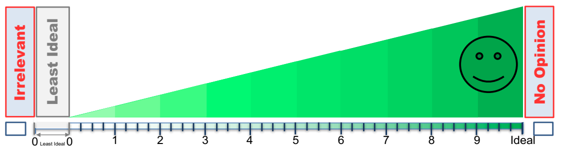

Ideal Scale
How close does this get to being ideal?

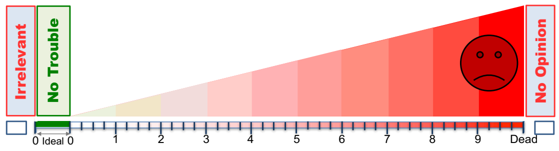

Trouble Scale
How bad are the troubles caused by this?

Naming Forms v1, ready to use for a new naming project, licensed under Creative Commons CC-BY 4.0 International, © 2016 L. Loewe.
Please cite http://evolvix.org/naming and this paper when reporting naming work or introducing names improved by using these forms:
Loewe, Scheuer, Keel, et al. (2016) 'Evolvix BEST Names for semantic reproducibility across code2brain interfaces', Ann.N.Y.Acad.Sci.

S1 Form SubmitTag **Submission** for Naming Project ProjectTag – Request4SubmissionsTag – if known __P-__ Page __ of __ Total Pages

Do not submit any important secrets ever. We work to be confidential, but thieves still exist. **Consider submitting anonymous reviews**. Read below about why your frank opinion is key.

Your ContributorTag(s) to mark *all* pages: **No real person names if you want to increase anonymity** (Any random letter combo works!) ________

Date of Submission: YYYY______/MM____/DD____ Work time __ Optional __ over time period _ Optional _

Your choices about this Submission: If you check nothing, we assume that our default entries YES, NO, or MISS (meaning no info) are assumptions you want us to make about you. Please expand on any non-default options you choose and other relevant details in comments attached to this submission as you see fit. We may or may not use what you submit as Names, Definitions of Meaning or Ways of Expressing it, as well as any reasons arguing for or against these particular syntax or semantics constructs. We will ignore and if feasible may delete all other information not relevant to the Naming discussions facilitated by these forms. Our use of your submission is regulated by the contributor's license agreement that governs all contributions to this open source software project (see its website). Briefly, you may submit only those of your naming ideas that you are happy to see published. If you know or suspect that any license or other intellectual property limitations affect any part of your naming submission, you must clearly say so on this page and attach all relevant details. In all other cases, we can use your submission freely as specified. For each naming contribution you submit to us, you give us the non-exclusive, transferrable, irrevocable, royalty-free and world-wide right to publish it under any approved open source or creative commons license. As a contributor you still own the copyright for your ideas and can use them as you like, but submitting your ideas allows others to use them too if published by this open source project. Hence, please keep to yourself what you do not want to share freely. There is no right for submissions to be used or acknowledgements to be processed, especially not for unused submissions. Many submissions will not outweigh a single submission with a better solution it can take a long search to improve good names that have to meet many requirements. We are happy to acknowledge your work on substantial naming searches if you wish and give us the necessary acknowledgements info.

1. [YES | NO | Comment Attached] For this submission, **you prefer to maximize anonymity**
2. [YES | NO | Comment Attached] You allow us to **use all your ideas in this submission** as detailed above.
3. [NO | YES | Comment Attached] You would like to be **acknowledged** on the web for **general naming work**
4. [NO | YES | Comment Attached] You would like to be **acknowledged** for particularly **especially useful ideas** using this acknowledgement info:

5. **Overall Self-Assessment** only for Naming efforts touched by ***this*** **submission**
   With respect to anything e.g. history, names, meanings, ways of expressing them, problems, concepts, research, etc. relevant for particular Naming efforts advanced by this submission: **do you think you are**
   [_] total outsider, [_] beginner, [_] rare drop-in, [_] trainee, [_] core contributor, or [_] expert **in this *Specific* Naming Effort**
   [_] Are you a leading research expert on some of the issues raised here (stay anonymous or tell us how we can ask questions)

It can help if you answer the following, but **please don't answer if you like more anonymity**
([MISS]: is the default answer we assume if you give no answer; check MISS to confirm this; else check other boxes with answers)

1. [MISS | NO | Yes | Comment Attached] Do you personally enjoy **reading** (any style or genre)?
2. [MISS | NO | Yes | Comment Attached] Do you personally enjoy **writing** (any area; any style)?
3. [MISS | NO | Yes | Comment Attached] Do you personally enjoy **researching** (any area; any style)?
4. [MISS | NO | Yes | Comment Attached] Do you enjoy **explaining/teaching** (any style; any area)?
5. [MISS | NO | Yes | Comment Attached] Do you enjoy **planning/organizing** (any style; any area)?
6. [MISS | NO | Yes | Comment Attached] Have you ***ever*** **programmed** anything in a computer language?
7. [MISS | NO | Yes | Comment Attached] Have you *ever* implemented *any* **function/method** in computer code?
8. [MISS | NO | Yes | Comment Attached] Do you feel that you understand *any* **pointers** in programming?
9. [MISS | Ignore | Like | Comment Attached] Do you **like / ignore algebra** or *any* other type of mathematics?
10. [MISS | Ignore | Like | Comment Attached] Do you **like / ignore programming** or *any* work with computers?
11. **How much learning and research have you done so far?** [MISS] Check all that apply:
    Learning in [MISS] [_] Primary School, [_] Middle School, [_] High School, [_] College for Graduation, [_] __
    Research [MISS] [_] as PostGrad, [_] as Postdoc, [_] in Industry, [_] at Uni, [_] at Home, [_] as Prof, [_] ___
12. **Do you see yourself as literate in these areas?** [MISS] Check all that apply:
    Any [_] arts, [_] business, [_] computational, [_] educational, [_] humanities, [_] law, [_] science, [_] social

Naming Forms v1, ready to use for a new naming project, licensed under Creative Commons CC-BY 4.0 International, © 2016 L. Loewe.
Please cite http://evolvix.org/naming and this paper when reporting naming work or introducing names improved by using these forms:
Loewe, Scheuer, Keel, et al. (2016) 'Evolvix BEST Names for semantic reproducibility across code2brain interfaces', Ann.N.Y.Acad.Sci.

Your Naming Perspective: Detailed Self-Assessment

Do not use if you want to maximize anonymity!
Everybody brings a special perspective to naming work; finding Names that work for a very broad audience benefits from integrating the views of many perspectives. Skip the questions below if you want to maximize anonymity, but if this is not an issue for you, your answers will help improve the overall balance. Please omit answering any questions that you are not comfortable answering here:

1. **Which areas do you work in and what do you use there?** [MISS] Check all that apply if any:
   Working with [MISS] [_] any physical skills [_] architectures [_] arts [_] code [_] computers [_] consulting [_] design [_] education [_] engineering [_] infos [_] laws [_] machines [_] math abstractions [_] medical [_] models [_] news [_] science [_] social skills [_] words [_] workflows [_] _______
   Coding as [MISS] [_] Coder [_] Programmer [_] Software developer [_] Software engineer [_] Designer [_] Computational scientist [_] Big data analyst [_] System administrator [_] Software architect [_] _______

2. **Detailed Self-Assessment on Naming Related Skills**
   Total Outsiders and Beginners are often excellent reviewers of Usability for the General Public, hence they can contribute pivotally important insights, even if they may not feel it at the time.

   Some problems are best solved by experts, and some best by newcomers. Indicating where you see yourself in this particular naming discussion will help maximize the impact of your contributions, especially any comments where you simply indicate “confusion”.

   With respect to the history, problems, concepts, science, names, meanings and ways of expressing them as relevant for **this current Naming discussion, do you consider yourself to be a**

Usability Reviewer

[_] Total Outsider, [_] Beginner, [_] Rare user, [_] Trainee, [_] Advanced user, [_] Expert in Usability for **General Public**
[_] Total Outsider, [_] Beginner, [_] Rare user, [_] Trainee, [_] Advanced user, [_] Expert in Usability for **Rare Users**
[_] Total Outsider, [_] Beginner, [_] Rare user, [_] Trainee, [_] Advanced user, [_] Expert in Usability for **Power Users**
[_] Total Outsider, [_] Beginner, [_] Rare user, [_] Trainee, [_] Advanced user, [_] Expert in Usability for **Expert Users**
[_] Total Outsider, [_] Beginner, [_] Rare user, [_] Trainee, [_] Advanced user, [_] Expert in Usability for **Outside Experts**
[_] Total Outsider, [_] Beginner, [_] Rare user, [_] Trainee, [_] Advanced user, [_] Expert in Usability for **Programmers**
[_] Total Outsider, [_] Beginner, [_] Rare user, [_] Trainee, [_] Advanced user, [_] Expert in Usability for **Administrators**
[_] Total Outsider, [_] Beginner, [_] Rare user, [_] Trainee, [_] Advanced user, [_] Expert in **Visual-Interface**-Usability
[_] Total Outsider, [_] Beginner, [_] Rare user, [_] Trainee, [_] Advanced user, [_] Expert in **Ways-of-Expression**-Usability
[_] Total Outsider, [_] Beginner, [_] Rare user, [_] Trainee, [_] Advanced user, [_] Expert in **General-Overall**-Usability

Syntax and Semantics of Human Language, Code or Problem Area Reviewer

[_] Total Outsider, [_] Beginner, [_] Rare user, [_] Trainee, [_] Advanced user, [_] Expert in **English Ambiguities**
[_] Total Outsider, [_] Beginner, [_] Rare user, [_] Trainee, [_] Advanced user, [_] Expert in **English Language Meaning**
[_] Total Outsider, [_] Beginner, [_] Rare user, [_] Trainee, [_] Advanced user, [_] Expert in **Problem-Area Details**
[_] Total Outsider, [_] Beginner, [_] Rare user, [_] Trainee, [_] Advanced user, [_] Expert in **Problem Domain Concepts**
[_] Total Outsider, [_] Beginner, [_] Rare user, [_] Trainee, [_] Advanced user, [_] Expert in **Ways of Expressing Meaning**
[_] Total Outsider, [_] Beginner, [_] Rare user, [_] Trainee, [_] Advanced user, [_] Expert in **Programming Syntax Design**
[_] Total Outsider, [_] Beginner, [_] Rare user, [_] Trainee, [_] Advanced user, [_] Expert in **Ambiguity in Logic and Code**
[_] Total Outsider, [_] Beginner, [_] Rare user, [_] Trainee, [_] Advanced user, [_] Expert in Meaning in **Logic and Code**
[_] Total Outsider, [_] Beginner, [_] Rare user, [_] Trainee, [_] Advanced user, [_] Expert in Meaning in **Math and Stats**

Software Architecture and Development Reviewer

[_] Total Outsider, [_] Beginner, [_] Rare user, [_] Trainee, [_] Advanced user, [_] Expert in General **Programming**
[_] Total Outsider, [_] Beginner, [_] Rare user, [_] Trainee, [_] Advanced user, [_] Expert in **Solution Implementing**
[_] Total Outsider, [_] Beginner, [_] Rare user, [_] Trainee, [_] Advanced user, [_] Expert in **Solution Design** Details
[_] Total Outsider, [_] Beginner, [_] Rare user, [_] Trainee, [_] Advanced user, [_] Expert in **Solution Architecture** Design
[_] Total Outsider, [_] Beginner, [_] Rare user, [_] Trainee, [_] Advanced user, [_] Expert in **Overall System** Architecture

Specific Naming Effort Reviewer

[_] Total Outsider, [_] Beginner, [_] Rare user, [_] Trainee, [_] Advanced user, [_] Expert in This ***Overall*** **Naming** Effort
[_] Total Outsider, [_] Beginner, [_] Rare user, [_] Trainee, [_] Advanced user, [_] Expert in This ***Specific*** **Naming** Effort
[_] Leading research expert on any issues raised here, preferring to stay anonymous
[_] Leading research expert offering follow-up discussions as needed for clarifying key points
(Please give an email to allow follow up if needed)

Naming Forms v1, ready to use for a new naming project, licensed under Creative Commons CC-BY 4.0 International, © 2016 L. Loewe.
Please cite http://evolvix.org/naming and this paper when reporting naming work or introducing names improved by using these forms:
Loewe, Scheuer, Keel, et al. (2016) ‘Evolvix BEST Names for semantic reproducibility across code2brain interfaces’, Ann.N.Y.Acad.Sci.

MForm MeaningTag defining **1 Meaning** of Temporary Meaning TagName________________________ P- Page_of_Total Pages

ContributorTags: **No personal info here!** Reuse random ContributorTags from Submit-Form! Date: YYYY____/MM__/DD__VV_____v Version_r Release_p Patch_

Before you fill this out or add your name, please READ ALL INSTRUCTIONS **at the beginning.** If you run out of space, **please use the question tags** to clearly identify additional content on extra pages.

m1: ***What is meant** by the concept?*

Define the meaning(s) at the core and/or in the scope of this concept:

m2: ***What is not meant** by the concept?*

*Contrast it with similar concepts that are **outside** of the scope of this concept (to sharpen the definition):*

m3: ***Why is this concept important?***

Explain motivation and need for representing this:

m4: ***Which words / phrases** best convey the concept?*

List diverse ideas/words/phrases/synonyms for inspiring good descriptions of key aspects:

m5: ***What is it supposed to do?***

List what its intended purpose or function is in the context given:

m6: ***Who will use it** how and where?*

Describe one or a few brief examples of typical use (with contexts and users):

m7: ***How mature** is the meaning of the concept itself?*

Assess quality of concept (not of its description): science, stability, maturity, problems…

m8: ***How mature** are the ways of expressing this meaning?*

Assess quality of ways of expressing (not of the concept itself): stability, maturity, problems…

m20: **Rating by Original Authors:**__MIS – KO – OKO – OK as of YYYY_MM_DD Date________________________

--**Internal Evaluation After Submission**--

m21: **Rating by Expert** Reviewers:__MIS – KO – OKO – OK **at** YYYY_MM_DD Date________________________

m21: **Rating by Usability** Reviewers:__MIS – KO – OKO – OK **at** YYYY_MM_DD Date________________________

m23: **Rating by Language** Reviewers:__MIS – KO – OKO – OK **at** YYYY_MM_DD Date________________________

m24: **POST** Workflow **Key Dates**: **AA** at YYYY___MM___DD___ **Hot** at YYYY___MM___DD___ **Slow** at YYYY___MM___DD___ **HH** at YYYY___MM___DD___

Mark below all POST codes that best describe the state of this Naming Form (if all is unchecked, 'Usual State' is selected by default; all others need action):

POST zone		Usual State (default)	Other States (check to activate)			
Mark **Workflow**	as ➔	[] **No Work**	[] **AnyArrival**	[] BackBurner	[] **AnyAimsAdmin**	[] **Priority:**____
Mark **Info**	as ➔	[] **No Info**	[] **CollectedContent**	[] **DesignDoc**	[] AnyHelpArea	[] **LabLog**
Mark **Restricted**	as ➔	[] **No Restricted Sharing**	[] **FeedbackFlow**	[] **EnclosedEffort**	[] Publication material	[] **Private to** Workspace
Mark **Scratch**	as ➔	[] **No Scratch**	[] **JammedJob**	[] KnownKiller	[] InternalItem	[] LabLog
Mark **Archival**	as ➔	[] **No Archival**	[] YesYet	[] UploadUsed	[] VersionVariant	[] HistoryHeap
Mark **InstantStartUp**	as ➔	[] **No InstantStartUp**	[] **GrandGallery**	[] **UploadUsed**	[] HistoryHeap	[] AnyArrival
Mark **Stabilizing**	as ➔	[] **No Internal Stabilizing**	[] MockupModel	[] NewNonfunctional	[] OperatesOften	[] PreProbing
		[] **No Public Stabilizing**	[] **QualityQuest**	[] **ReviewedRelease**	[] **StableSource**	[] **TrustedTested**

Naming Forms v1, ready to use for a new naming project, licensed under Creative Commons CC-BY 4.0 International, © 2016 L. Loewe.
Please cite http://evolvix.org/naming and this paper when reporting naming work or introducing names improved by using these forms:
Loewe, Scheuer, Keel, et al. (2016) 'Evolvix BEST Names for semantic reproducibility across code2brain interfaces', Ann.N.Y.Acad.Sci.

WForm MeaningTag assessing **2 Ways to express 1 meaning** of Temporary Meaning TagName__P- Page_of_Total Pages

ContributorTags: **No personal info here!** Reuse random ContributorTags from Submit-Form! Date: YYYY____/MM__/DD__VV_____v Version_r Release_p Patch_

Before you fill this out or add your name, please READ ALL INSTRUCTIONS **at the beginning.** If you run out of space, **please use the question tags** to clearly identify additional content on extra pages.

Proposal ID *\ Name*	1 \	2 \
Details of expression:<br>*Exact words/punctuation/etc.*<br>*Give example(or formal syntax):*		
Precise meaning:<br>*Specify exact, nuanced meaning to your greatest ability:*		
Connotations:<br>*Specify your interpretation(s) of the meaning to your greatest ability (e.g. what does the expression mean to you?). Mark as + (good) or – (bad):*	1.<br>2.<br>3.	1.<br>2.<br>3.
Associations:<br>*Specify any other concept, thing, etc. that you associate with the expression (e.g. what does your mind "link" the name to?). Mark as + (good) or –(bad):*	1.<br>2.<br>3.	1.<br>2.<br>3.
Ambiguities:<br>*Specify any alternative meanings or interpretations of the expression that could cause different people to understand the expression differently:*	1.<br>2.<br>3.	1.<br>2.<br>3.
Strengths:<br>*Specify what you see are the main benefits of adopting this expression for the given meaning:*	1.<br>2.<br>3.	1.<br>2.<br>3.
Weaknesses:<br>*Specify what you see are the main problems with adopting this expression for the given meaning:*	1.<br>2.<br>3.	1.<br>2.<br>3.
Synonyms:<br>*If possible, specify any synonyms for this expression that you can think of. Mark as + (better) or –(worse):*	1.<br>2.<br>3.	1.<br>2.<br>3.
Syntax Author:		
Resource(s) used:	Please improve this list:<br>[] Merriam-Webster (m-w.com)<br>[] Thesaurus.com<br>[] Wiktionary.com<br>[] Longman's (ldoceonline.com)<br>[] OxfordDictionaries.com<br>[] Other:	Please improve this list:<br>[] Merriam-Webster (m-w.com)<br>[] Thesaurus.com<br>[] Wiktionary.com<br>[] Longman's (ldoceonline.com)<br>[] OxfordDictionaries.com<br>[] Other:
**Decision:**	MIS – KO – OKO – OK	MIS – KO – OKO – OK
Other Comments:		

Naming Forms v1, ready to use for a new naming project, licensed under Creative Commons CC-BY 4.0 International, © 2016 L. Loewe.
Please cite http://evolvix.org/naming and this paper when reporting naming work or introducing names improved by using these forms:
Loewe, Scheuer, Keel, et al. (2016) 'Evolvix BEST Names for semantic reproducibility across code2brain interfaces', Ann.N.Y.Acad.Sci.

R1Form _MeaningTag_ rating **2 ways to express 1 meaning** of _Temporary Meaning TagName_ ______ P- _Page_ _ of _ _Total Pages_

ContributorTags: **No personal info here!** Reuse random ContributorTags from Submit-Form! Date: _YYYY_ _____ / _MM_ __ / _DD_ __ VV ______ v _Version_ _ r _Release_ _ p _Patch_ _

Before you fill this out or add your name, please READ ALL INSTRUCTIONS **at the beginning.** If you run out of space, **please use the question tags** to clearly identify additional content on extra pages.

Please rate all traits of the given expressions on these scales

Check one of the vertical boxes at the extremes if the criterion is not applicable or you want to abstain from rating ("non-votes" strengthen the voices of other raters). You can choose intermediate values.

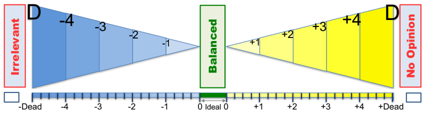

Balance Scale:
How well is the given trade-off managed?

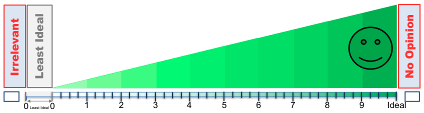

Ideal Scale:
How close does this get to being ideal?

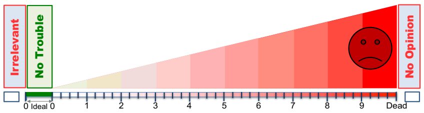

Trouble Scale:
How bad are the troubles caused by this?

Proposal ID *\ Name*	1 \	2 \
Details of expression:<br>*Exact words/punctuation/etc.*<br>*Give example(or formal syntax):*		
Criterion Rating:		
**Ambiguity**<br>Can you interpret the sentence formalities/words/punctuation very differently?	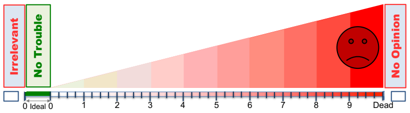	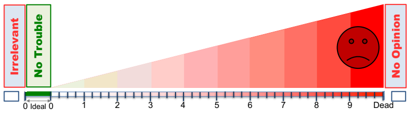
**WritingComplexity**<br>How much learning is needed for writing this?	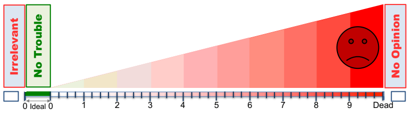	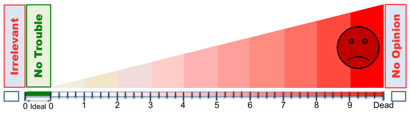
**ReadingComplexity**<br>How much learning is needed for understanding?	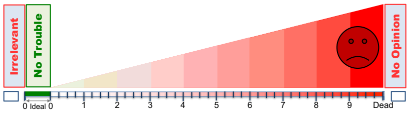	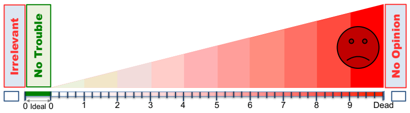
**MemoryComplexity**<br>How much learning is needed for remembering this?	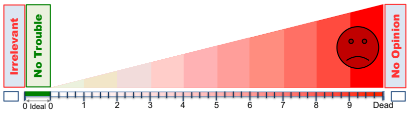	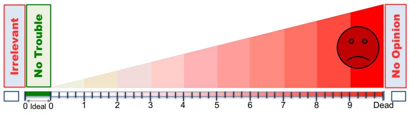
**Artificiality**<br>How contrived/artificial (bad) does this sound?	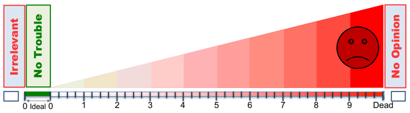	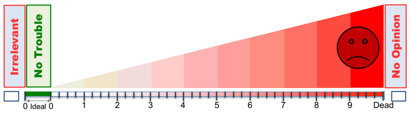
**Offensiveness**<br>How offensive is this for some audiences (dialects?)	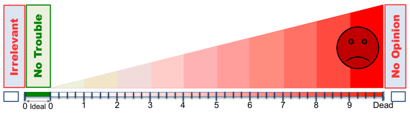	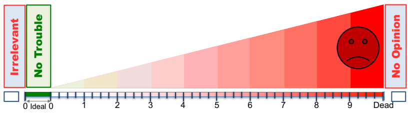
**CommonSense**<br>Will non-programmers understand this? (Give best assessment).	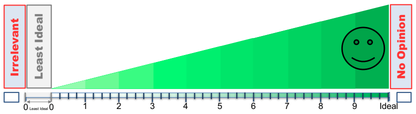	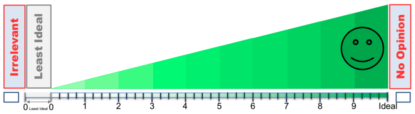

Naming Forms v1, ready to use for a new naming project, licensed under Creative Commons CC-BY 4.0 International, © 2016 L. Loewe.
Please cite http://evolvix.org/naming and this paper when reporting naming work or introducing names improved by using these forms:
Loewe, Scheuer, Keel, et al. (2016) 'Evolvix BEST Names for semantic reproducibility across code2brain interfaces', Ann.N.Y.Acad.Sci.

R2Form MeaningTag rating **2 ways to express 1 meaning** of Temporary Meaning TagName______P-___Page___of___Total Pages

ContributorTags: **No personal info here!** Reuse random ContributorTags from Submit-Form! Date: YYYY____/ MM___/ DD___VV______v Version___r Release___p Patch___

Before you fill this out or add your name, please READ ALL INSTRUCTIONS **at the beginning.** If you run out of space, **please use the question tags** to clearly identify additional content on extra pages.

**CoderSense**<br>Will programmers understand this? (Give best assessment).		
**YouthSense**<br>Will middle-school students understand this? (Give best assessment).		
**Intuitiveness**<br>Is the meaning of the words easy to understand intuitively?		
**Innovation**<br>How innovative/helpful (good) does this sound?		
**Elegance**<br>How elegant/beautiful is the proposed solution to read?		
**VisualAppeal**<br>Does the visual form support function and simplify reading?		
**Brevity**<br>How much typing does this need? Is it fast to use?		
**Practicality**<br>How easy is this to use without documentation?		
Trade off: **Regularity vs English**<br>Code simplifying rules vs natural language rules: well integrated?		
Trade off: **Length vs Meaning**<br>Brevity vs explicit meaning?		
**AlignmentFit**<br>How well does it align with *other words of related types in the language*?		
**OverallFit**<br>How well does it fit in the context of the *rest of the language*?		
Summary of Scores:		
**Decision:**		

Naming Forms v1, ready to use for a new naming project, licensed under Creative Commons CC-BY 4.0 International, © 2016 L. Loewe.
Please cite http://evolvix.org/naming and this paper when reporting naming work or introducing names improved by using these forms:
Loewe, Scheuer, Keel, et al. (2016) 'Evolvix BEST Names for semantic reproducibility across code2brain interfaces', Ann.N.Y.Acad.Sci.

Introducing
Project Organization Stabilizing Tool (POST) system *ReviewedRelease v1*

for evolving order and stability from innovation in chaotic environments

Loewe et al. (2016-09-12) Brief title “**POSTsystem *RRv1***”

Citation: http://dx.doi.org/10.1111/nyas.13192 – Updates: http://evolvix.org/post

Abstract

Organizing is the art of avoiding inessential complexity. Organizing becomes more difficult with project size, as increasing numbers of moving parts tend to throw everything into disarray more quickly. Good abstractions can enable amazing efficiencies by balancing the needs of standardizing and customizing, but they can be (too) costly to find. Much of this cost is related to naming, which in turn is closely related to organizing. Names are made to cut search times by labeling boxes of organized content. Poor naming strategies result in poor organization and increased search times. The quality of a naming/organizing strategy shows as numbers of items increase. Almost no strategy is needed for a few items (tempting many to regard naming as trivial), but finding appropriate strategies for millions of items or more is usually difficult (some say impossible). The lack of sensible strategies can frustrate much further research, a research potential that can be unlocked by the proposal of a workable nomenclature (i.e. naming strategy; see, for example, how modern biology was impacted by the organization and names proposed by Linnaeus' taxonomy).

Cancer cell biology, evolutionary systems biology, and many other areas of current biology are in great need of naming/organizing the millions of parts required for mechanistic computer simulations of relevant biological systems that incorporate all current knowledge. Many diverse ontologies, taxonomies, databases, genome-browsers, models, tools, simulations and other projects have made great progress in consolidating the dizzying jungle of synonyms in biology. However, their independent origins have also generated a new jungle of (near-)synonyms, as each project tends to use idiosyncratic ways of handling equivalent tasks, such as tracking bugs, tasks, reliability, versions, recurring types of modifications and more. These and many more inessential differences make it near impossible to develop programs that treat the scattered big data of biology in a uniform way. Most importantly, there is no universally agreed stability scale that enables researchers from distant fields to easily track the reproducibility, maturity, and reliability of a given result as assessed by relevant experts. A similar stability scale is also essential for developing programming languages aiming to respect the time of their users by not breaking the code of programs when releasing the next version (i.e. by providing long-term backwards compatibility).

The need for such a high-quality stability scale in the modeling language Evolvix inspired the development of the POST system presented here. It uses the BEST Names concept that disentangles conflicting naming priorities by distinguishing Brief, Explicit, Summarizing, and Technical (BEST) Name Dialects. Most Brief Names in POST are double-capital letters such as *RR*, *SS*, and *TT* that mirror Explicit Names like *ReviewedRelease*, *StableSource*, and *TrustedTested* respectively, where *TT* marks the difficult to achieve long-term backwards-compatible end of the POST sequence of stability levels that starts with *MM* for *MockupModel*. The rest of POST evolved around this *StabilizingZone* in order to provide the project organization necessary for the development of the Evolvix programming language and to support the simulation system it implements.

... from so simple a beginning endless forms most beautiful
... are being evolved.[1]

1. Preface[1]

It is a truth universally acknowledged, rarely pure and never simple: managing complex projects is … complex. Surprisingly, none of them start that way. Even the most complex projects are born from simple, elegant ideas in the mind(s) of their initiators before they begin to grow and evolve.

As humans, we generally abhor (ugly) complexity and admire (elegant) simplicity. Thus we will continue to start new projects as we try to 'stand out' while 'blending in', and 'be extraordinary' while 'remaining normal' (defined in as many ways as people exist on the planet).

These tensions are not new, and those before us have pioneered two response strategies: *standardize* and *customize*. *Standardizing* helps with simplicity, blending in, and compatibility to others, while *customizing* enables extraordinary feats of outstanding innovation – albeit at the cost of having to tolerate some inessential and annoying complexity.

Take cars, for example. Most people want a car (*standardize*), usually one that is different from others (*customize*). The pattern continues for driving behavior: roads require driving on the right side (which-ever that may be; *standardize*), while most people are free to choose which road they take (*customize* their journey). Our social contracts to pre-decide on which side of the road to drive are not imposed on us by the physics of cars; yet they have huge practical and technical consequences that simplify and speed up many decisions, save countless lives – and even affect cars physically.

We suggest that parallels exist to navigating multiple complex information-based projects efficiently. When working with multiple projects, it cannot be efficient when each project stores its tasks in a different location or has a different system for indicating stability. The POST system distills the best ideas for standardizing the organization of complex information processing projects of any kind.

---

[1] **Who may want to consider using POST?** We know from experience that most researchers, developers, and organizers are right in *not* looking for systems that can manage multiple complex projects; mostly, because what they already use works well for them and searching for better systems would waste much time (see below for reasons). The POST system was developed because of very real needs encountered in developing Evolvix; we realized only later that POST is much more widely applicable, more flexible, and easier to use than we had thought (and most people suspect). To highlight this generality, we added this broad introduction that explains how POST could be used efficiently for very many information based projects (see text up to example in Figure P1). However, we do not wish to imply that all such projects would benefit from switching (potentially at great cost). POST was designed for complex networks of interacting projects that have much to win from standardizing (see the computational biology use-cases described), and it may also get used by those who like the POSTcode names we found; but *if there is no need for improving coordination, there is little need for considering the POST system presented here.*

Why would most people be wasting time when evaluating systems for managing multiple complex projects? Such searches tend to be too tedious for anybody who wants to get started with actual work. So much so, that *ad hoc* strategies 'made-up along the way' quickly become irresistible; there is rarely a point in paying the large up-front costs of evaluating and introducing a complex system while a project is small and uses only a fraction of the capabilities. In comparison, it is much more efficient to use most capabilities of a system that has no up-front costs (such as naming a few folders and keeping a few tasks on a list). The *adhocery* of the latter does not matter if the project remains small or both grow together; transitioning later to an appropriately capable management system is usually not prohibitive for one such project. Attempting to avoid that cost is tricky, as there is no guarantee that the choice of a given large complex management system will pay off: it may turn out to lack *one* critical feature that forces complete re-organization or limits further growth. Given how unpredictable such critical features are in real life, most small projects do best to pick any system available at (next to) no startup cost – lest the project itself will never get started. Exceptions to these rules of thumb are projects aiming to set up structures that are intended to become long-term backwards compatible and/or projects that need to coordinate across many potentially independent subprojects, and thus require replicated and dependable structures (such as Evolvix).

Thus, a firm requirement for developing this POST system has been to welcome new users by keeping initial startup simple, albeit without killing projects indirectly later by no longer being able to support their growth.

2. Overview

POST aims to *standardize* aspects of organizing that do not really change between projects, and 'pick one side of the road' by choosing standardized names for frequently recurring needs to store certain types of information. It then offers projects to *customize* any given POST Home Folder for its specific purpose by allowing certain types of folders to be present or not. Using POST is no guarantee for the success of a project like driving on the right side of the road will not guarantee that a journey serves its purpose. However, those who give the POST system a try may find some of its catchy Brief and Explicit Names to be memorable, and the organizational support offered to be surprisingly useful. Progress in projects becomes much simpler if there is no need to arrive at customized decisions about which side of the road to pick on each turn of each project's journey. This more general perspective may help to set the frame for the more specific introduction of POST that follows next.

The Project Organization Stabilizing Tool (POST) system has been designed for any

- **Project** or complex set of tasks that depend on information processing in need of good
- **Organization** for improving work efficiency and reducing inessential complexity by
- **Stabilizing** flows of information using well-defined containers, building an automatable
- **Tool** that is easily customized and can help to separate the wheat from the chaff in messy project data while simultaneously encouraging creativity, rapid innovation, testing, and the slow emergence of reliable standards with long-term stability.

First we will introduce the POST system with a brief overview aimed at a more general audience; then we will highlight the original use-case it was developed for in computational biology, before we will switch gears and summarize important features of its design. We will also address the question of how to define progress towards long-term backwards compatibility, including an assessment of its current status, and areas of further development, all of which will be important as the POST system evolves towards increasing stability. An overview of the next sections follows:

3. Introduction (for beginners)
3.1. Motivating problem: the need to organize
3.2. Solution: organize project content using BEST Names (Table P1, Table P2)
3.3. 'Hello world' example: Using a POST Home Folder to write a paper together (Figure P1)
3.4. High-level structure of the POST system
3.5. Overviews of POST system definitions using BEST Names (pointing to Figures P2, P3)
4. Computational biology use-cases (for computational biologists)
4.1. Why TrustedTested (TT) Stability could be pivotal for personalized medicine and evolutionary systems biology.
4.2. Controlled vocabulary in POST
4.2.1. Area-specific controlled vocabulary lists in POST
4.2.2. Extensions of Top Level Key words in Brief Dictionary
4.2.3. Flexible Modifiers of other keywords in the Brief Dictionary
4.3. Defining *TrustedTested* in POST
5. Design (for software designers)
5.1. Functional requirements and features
5.2. Rules for automating and extending POST Home Folders
5.3. Translation in POST folders
6. POST specification: current status, stability and future work
7. References and POST overview figures: (i) InfoFlow in POST, (ii) Brief Dictionary of POST BEST Names.

This overview and the last two highly condensed pages provide perspectives on the POST system, that is defined by its BEST Names that specify a nucleus for developing POST standard semantics.

3. Introduction

This part is aimed at a more general audience of potential POST users.

3.1. Motivating problem: the need to organize

Ever wondered how to stay on top of a dynamic project that requires collecting and processing a lot of information? How to track the many improvements, small and big, that make a polished final version? Where to keep to-do lists, lessons learned, current problems? How can the final product be separated from all the information needed to produce it without making further improvements cumbersome? These challenges compound as projects become more complex, more contributors join, and the quest for quality intensifies in order to develop an outstanding product meant to last for a long time. The reason is:

Every contributor needs the right information at hand to make a difference and be efficient.

It is natural for many people to sort information into hierarchical *ad hoc* file trees and other organizational structures created on the fly. However, such improvisation can make it very difficult to work together efficiently, especially if organizational problems are solved in very different ways by the people involved. For example, different contributors might regularly prepare files with tasks to be delegated to other contributors, but everybody stores them in a folder with a different name, such as 'Delegated', 'Tasks', 'Todo', 'Work', 'Actionable', and so on. As naming preferences multiply, collaborators will spend an increasing amount of time communicating where files are (or risk that tasks remain undone and work is being misplaced). If this confusion is to be avoided a common project management strategy is required, but there is rarely enough time to develop it when the project "just needs to get done". To adopt an existing strategy may trigger a possibly prohibitively complex evaluation process to ensure that the system of choice meets all needs without imposing undue costs from inessential complexity.
Our efforts to support programming language developers and users have revealed the need for a simple-yet-scalable organizational scheme that makes it quick to start small projects in a way that does not prohibit them to gradually grow until they become complex long-term projects. Ideally, this scheme also quantifies the stability of different parts of large, complex projects and provides stable points of reference that can be used to measure progress towards aims of long-term stability (and backwards compatibility) without stifling the innovative processes that put a project on the map in the first place (the *"StabilizingZone"* introduced below aims to accomplish this).

3.2. Solution: organize project content using BEST Names

We developed the POST system described below to organize the development of the Evolvix programming language and its many nested smaller projects in a stable network of well-defined containers. We aimed to avoid recurrent, costly episodes of complete re-organization (such as the unavoidable one that triggered our work on POST). Due to the broad scope of Evolvix, we designed POST to offer a flexible infrastructure for supporting the development of very diverse project types.
At its core is a set of carefully chosen standard "*StabilityCodes",* or *"POSTcodes"*, aimed at separating highly refined information that moves very slowly from more transitory to more stable states as it is reviewed with increasing rigor. These StabilityCodes form a sequence that follows the alphabet and defines "DoubleCaps" as keywords, starting with MM, NN, OO, … and ending with … RR, SS, TT:

**MM**	MockupModel	MockupModel_UsedFor_RapidPrototyping_InformalLearning__ __ExpertimentsToBeThrownAway_StabilizingDesignNotCode
**NN**	NewNonfunctional	NewNonfunctional_UsedFor_NotYetFunctioning_DeepFoundations__ __ForLargerStableDesigns_ThatDoNotYetWorkForUsers
**OO**	OperatesOften	OperatesOften_UsedFor_Systems_PartiallyWorkingForEndUsers__ __while_StillMissing_ImportantFeatures_ToBeImplemented
**PP**	PreProbing	PreProbing_UsedFor_Preparing_PeerReviewAndPublicProbing__ __by_PolishingExistingFeatures_UntilSubmissionFor_Questioning
**QQ**	QualityQuest	QualityQuest_UsedFor_Questioning_AxiomsDataScienceAccuracy__ __RigorClarityUsability_InMany_ExpertBeginnerReviewRounds
**RR**	ReviewedRelease	ReviewedRelease_UsedFor_NewReleasesRecommended_by__ __QualityQuestEditors_after_AnsweringAllReviewerQuestions
**SS**	StableSource	StableSource_UsedFor_StunningSoftware_RunningInProduction__ __with_LongTermSuccess_and_VeryRareRevisionRequests
**TT**	TrustedTested	TrustedTested_UsedFor_Marking_VeryLongTermStableDesigns_in__ __WellUnderstoodDomains_AllowingBackwardsCompatibleGrowth

Table P1: All *StabilityCodes* (MM…TT) defining the *StablizingZone* of POST.

Other *POSTcodes* denote other aspects of project management that are independent from these *StabilityCodes* codes, but also reflect in some ways on the stability of the information they annotate (such as *AnyArrival* for arbitrary project relevant material that has just arrived, or *VersionVariant* for historic releases of variants). Drawing on the alphabet's familiar progression, the *POSTcodes* and *StabilityCodes* roughly organize the initial project phases with earlier letters and the increasingly mature phases with later letters.

These POSTcodes annotate containers, such as folders, with a short description of their intended purposes, and they exist as the first three standardized synonymous names defined by the BEST Names concept:

- **Brief** Names: here mostly double capital letters for speed, brevity, and memorability;
- **Explicit** Names: here mostly memorable keywords reflecting the capital letters; and
- **Summarizing** Names: cheat-sheet-like summary sentences that act like mini-manuals.
- **Technical** Names will be defined later to serve as core for corresponding StableNames.

Our use of BEST Names aims to enhance the intuitiveness and memorability of *POSTcodes*; indeed, without BEST Names it is unlikely that POST would have been developed. Many of its use-cases require very frequent use of POST codes at very short notice, that neither leave much room for typing nor much time for remembering cryptic names, as seen in the example given next.

3.3. Example: Using a POST Home Folder to write a paper together

*It is simple to start using POST***.** It can be as easy as moving old variants of processed files to a *HistoryHeap* (*HH*) folder for decluttering a project folder to increase efficiency (and avoid the much larger cost of determining whether historic variants of a file will ever be needed again for any purpose; storage is usually cheap). Additional folders follow as needed. To select them, the Summarizing Names from the Brief Dictionary of POST in Figure P3 can be extremely helpful. Here is a short list:

**AA**	AnyArrival	AnyArrival_UsedFor_Inbox_QuickDropping_Items_to_ProcessLater
**AAA**	AnyAimsAdmin	AnyAimsAdmin_UsedFor_Managing_StrategyAims_ProjectPlans__ __Workflows_RoleDefs_Tracking_TasksDeadlinesFunds__ __RecruitingTraining_Delegation_CallBacks_Prioritizing_KeyGoals
**BB**	BackBurner	BackBurner_UsedFor_Storing_SeasOfGoodIdeas_to_RevisitLater
**AHA**	AnyHelpArea	AnyHelpArea_UsedFor_OnlineHelp_UserManuals_InfoMessages__ __TeachingOutreachMaterial_Translatable_by_CountryCode
**CC**	CollectedContent	CollectedContent_UsedFor_Collecting_External_TopicIdeas_Data__ __Methods_Evidence_Refs_Reviews_Clarity_CodeExplanations
**FF**	FeedbackFlow	FeedbackFlow_UsedFor_Logging_AnyInputCommentCritiqueBug__ __Ideas_from_ReviewerExpertUserFriendFoeFans_Worldwide
**GG**	GrandGallery	GrandGallery_UsedFor_Presenting_BestExhibits_LatestResults__ __FinalKeyDeliverables_without_NeedForLongTermStability
**HH**	HistoryHeap	HistoryHeap_UsedFor_OldVersionBackup_ProbablyDeletableFiles__ __HoardedDataWithLimitedOrder_KeptFor_PotentialUsefulness
**JJ**	JammedJob	JammedJob_UsedFor_Documenting_BugReports_DecisionNeeds__ __and_ProgressOn_SolvableProblems _that_NeedSolutionsSoon
**LL**	LabLog	LabLog_UsedFor_Logging_AnyLabLabor_Dated_YYYY_MM_DD__ __ForHistoricSequenceOf_AllOutcomesOf_AllTests_IncludingFails
**UU**	UploadUsed	UploadUsed_UsedFor_Archiving_ExternalCommunicationLog_of__ __FullUploadsOf_InputTo_or_OutputFrom_GG_etc

Table P2: Brief, Explicit, and Summarizing Names of basic *POSTCodes* used for choosing in Fig P1.

For example, a research paper in the folder of *MyResearch* might select from this list the folders shown in Figure P1 below. Usually *MyResearch* starts with a very small folder, only storing any arriving ideas and information in an unstructured form in the *AnyArrival* (*AA*) drop box. As the collection of content continues, arriving files are sorted into *CollectedContent*. Planning prioritizes aims in *AnyAimsAdmin* (*AAA*) structures. The final latest and greatest version of the product produced will grow and reside in the *GrandGallery* (*GG*), and a *LabLog (LL)* may help record a chronology of the work done in preparation for the big finale. To declutter, old variants or unused files are moved from *GG* to the *HistoryHeap* (*HH*) and good ideas for later from *AAA* to *BackBurner* (*BB*). Writing and working often result in *JammedJobs* (*JJ*) that need solutions, and increasing project complexity might lead to a reorganization of content that results in new *GG* folders for different workspaces. Shared manuscript variants are stored as *UploadsUsed* (*UU*), and incoming comments collected in *FeedbackFlow* (*FF*). See Figures P2 and P3 for more potential workflows.

The complete POST system includes many more features than are needed in most use cases and is being designed to provide developers with powerful abstractions for facilitating the automation of data flows. However, none of this needs to infringe on personal organizing preferences for users who do not need automation. POST's beauty is in its flexibility: it encourages using of *StabilityCodes* as needed and ignoring of all others – they will wait until content arrives that they can organize.

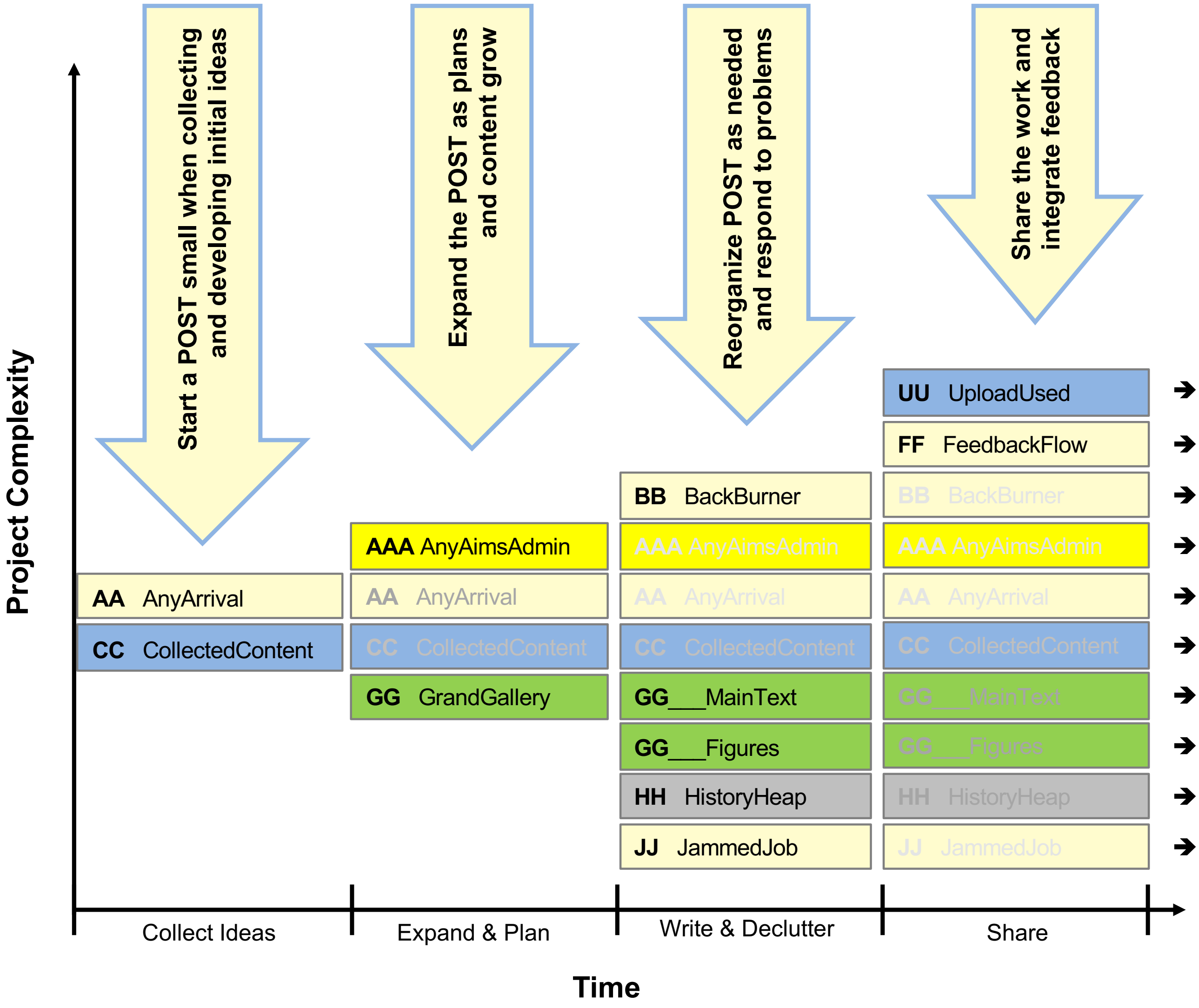

Figure P1: Using a POST Home Folder for organizing a mini-project such as writing a paper. Exemplary activities are shown in columns and the folders they generate in rows as they persist. The names presented here were found to be easy to understand with minimal explanations in our internal tests. More complete definitions using Brief Names (e.g. “AA”), Explicit Names (e.g. “AnyArrival”) and Summarizing Names (e.g. giving a short description of how to use “AA”) are given in Table P2 and the overviews of the POST system in Figures P2 and P3 below. Brief and Explicit Names are given here for clarity, but this would not usually be done in practice (Brief is enough). The POST system can tolerate a great number of variations when used manually, but many contributors usually benefit from stricter following the naming rules to avoid confusion, while POST automation requires following all formal rules. POST can help organize data in diverse storage media, including manual use on paper.

3.4. High-level structure of the POST system

Generality, flexibility and origin. The POST system is meant to be as general as reasonably possible so that anyone can use it for efficiently managing projects, small or large. This is the case even though (or rather because) it was primarily developed for supporting the implementation infrastructure of the general-purpose programming features of the Evolvix modeling language. This language aims to support a very broad set of use cases to enable efficient work in computational biology (hence the generality, which is needed to accommodate the broad diversity of biological use cases). The POST system facilitates standardizing and customizing development work across complex and independent code, text, and project management structures. Users can drop features they do not need initially, only to introduce them as necessary when a project keeps growing in complexity.

POST Home Folders (PHF), manual POSTboxes, and automated POSTnets.
The POST system is designed to encourage automated file management while also providing an effective structure for manual organization that makes it easy to set up and manually operate a single POSTbox that consists of a single POST Home Folder. Simultaneously it aims to support the growth of massively distributed collaborative projects that eventually combine many automatically managed PHFs into larger POSTnets (e.g. to meet citizen science project requirements described elsewhere [2]). Such networks facilitate globally distributed collaboration by automating well-defined information flows between individual PHFs, while clearly communicating the stability of the code involved (albeit without burdening users who do not need this for their POSTboxes).
We define

- a **POST Home Folder** (PHF) as the one top-level folder of a given POSTbox, containing its own independent active area, ZZ* folders and POST_Cabinet folder (which in case of automation must be stored under the name "_POST" or "a_POST", if leading underscores are forbidden; manual use is more flexible); all folders and data in a POSTbox is stored relative to the PHF, which serves as a local anchor in its broader context;
- a **POSTbox** as the implementation of a single PHF that allows for manual management – just like the old-fashioned mail service that requires hands-on file management and is simple to use; a POSTbox may have links to other POSTboxes (see ZZ, ZZA, ZZ* folders);
- a **POSTnet** as a combination of many PHFs that can easily become very intricate and difficult to keep in a consistent state manually; to maintain consistency, POSTnets are usually best managed automatically, especially when multiple PHFs are nested, interact in complicated ways, and/or contain data that is costly to re-synchronize after incomplete manual operations;
- the **POST system** (or simply 'POST') as the abstract type specification defined in this document or a future POST standard; it shows how to implement a POSTbox or POSTnet.

Nesting and automation. PHFs can be nested inside and linked to other PHFs, thus facilitating great flexibility as they can replicate and network at all levels of a project hierarchy. While such networks can be managed manually in principle, it is more likely that they require automated POSTnets to reduce operational errors.

Simple decisions before starting a POST Home Folder. A project using POST should consider:

- Will it be operated manually, like most project, and thus use the "*GrandGallery*" as place for developing and updating the latest content, adding other folders only as necessary?
  For a simple example, see the Figure P1 above.

- Will it aim for some form of stability or backwards-compatibility to help guard the investments of outside users who rely on the stability of the system developed by this POST? Then using the *StabilizingZone* is recommended to encourage stability, even if *TT* may not be the goal. Thus, new content is developed in *MM* or *NN*, and moved up the rungs of the *StabilizingZone* as it matures up to RR or SS, possibly with automated support.
- Will the PHF be automated eventually or manually managed throughout its life-time? Automated PHFs follow stricter rules that are easier to enforce from the start, even if managed manually. Costly re-organizations may be avoided by following the rules right away.

Rigidity of structure. If automation is not needed, then users of a PHF may interpret POST related structures more flexibly. Humans are much better at navigating ambiguity than computers, albeit consistent use has substantial advantages for humans as well, especially when many contributors are involved. For example, it is easy to append tags that serve as *WorkspaceIDs* to further break down navigation complexity, even if not yet supported by automation (e.g. see "*GG*-Figures" in Figure P1); it may not matter to humans, whether peer reviews are stored as *UploadUsed* (by reviewers) or next to potentially a lot of files inside of *FeedbackFlow* from the web, but automated PHFs need a standard answer allowing them to expect defined content in a fixed place and thereby reducing inessential code complexity. Most areas where the POST system requires additional work are such decisions of locating within the folders already defined in Figures P2 and P3. Such choices do not alter the core meaning of POST *StabilityCodes* and hence rarely matter in a manual POSTbox.

3.5. Overviews of POST system definitions using BEST Names

Nucleus for a POST system standard

While Figure P1 above provides an incomplete introductory overview of POST parts frequently used in simple projects, the Figures at the very end of this text provide complete overview perspectives of the core of all top level *POSTcodes,* including the *StabilityCodes* that facilitate the project organizing and stabilizing aspects of POST:

- Figure P2: Information flow overview illustrating some inner connections between *StabilityCodes* and which folder is located where (if a POST is implemented as folders in a file system, which is not the only possible option).
- Figure P3: Brief Dictionary of all core *POSTcodes* giving interchangeable BEST Names

The core *POSTcodes* presented in these figures can be complemented by more specific *POSTcodes* that belong to reserved and controlled vocabulary lists of keywords and that streamline the naming of more specific areas, such as organizing observed data, estimates of parameters and simulation results. Discipline, research-area or industry specific keywords lists are conceivable, whose applicability to a given POST Home Folder is defined by the type assigned to the PHF. Overall, the POST system design aims to minimize the number of these keywords; if possible to maintain, POST development will aim to make these sets of words non-redundant and free of “near-synonyms” to ensure overall simplicity of use by maximizing the re-use of core names.

The core names defined in these lists also form the core material for generating Technical Names that become part of the StableName and StableMeaning as defined by the BEST Names concept to ensure unambiguity in the presence of potentially many translations and (perfect) synonyms.

4. Computational biology use-cases

Next we will present details of the the use-cases that motivated the development of POST.

4.1. Why *TrustedTested* (*TT*) Stability could be pivotal for personalized medicine and evolutionary systems biology.

Not all software systems let alone non-IT systems require *TrustedTested* or *TT* stability. However, the equivalent of *TT* stability is critical for systems that depend on the results of very large numbers of contributors who need to build upon each other's work over very long periods of time to achieve overall success. This requires using a common language to improve their efficiency of communication; if the number of required contributors is so large that newcomers need to be recruited, then chances of success will increase dramatically if learning this shared language is easy. Modeling biological systems has shown that computational techniques can in principle be applied to vastly more biological systems than currently modeled. While this discrepancy may have many reasons, on important reason is certainly the lack of a *TT* stable modeling language that efficiently helps biologists to formally describe their mechanistic understanding of molecular and other systems they investigate (including experimental results) to allow for corresponding computational systems biology analyses. The emergence of some standardization (e.g. see SBML.org [3]) is encouraging, but there is a long way to go until the computational tools for biology can integrate current biological understanding well enough to

- enable **personalized medicine** analyses that efficiently build on all known evidence (and not only the datasets that happen to be available),[4] or
- enable **evolutionary systems biology** analyses predicting phenotypes and fitness from genotypes and environments, in order to simulate how populations are likely to evolve,[5]

or enable similarly bold and challenging visions in predictive cancer research, conservation biology, or development of policies to slow antibiotics resistance evolution in bacteria. All these areas will eventually need to combine biological observations, models and results from many decades in the past and in the future. Only a reliable computational integration can enable future biologists to efficiently build on the foundation of past results without losing them to semantic rot or requiring the steep cost of continually re-implementing past modeling code. Experiences with the first simulation project that used Evolvix [6] highlighted the potential for automating such an integration.

To efficiently enable personalized medicine or evolutionary systems biology it is essential to find a way to keep active the models that are currently being buried in immutable online publications. Activation could be greatly simplified by implementing models in code that is easy to read and has the *TTv1* stability allowing future biology students to easily extend old model code using new *TTv1* downloads.

This vision drives Evolvix, and inspired the quest to define a StabilizingZone leading to TrustedTested and the POST system. Evolvix adopts POST to increase code stability. There is no reason why other projects cannot use the StabilizingZone and POST to improve their stability as well.

4.2. Area-specific controlled vocabulary lists in POST

To avoid a confusing chaos of folder names, POST defines some keywords for the purpose of streamlining the locations that programs would have to know in order to find corresponding files. For example, in POST, simulation results should always be in a folder that has "**Sim**" in the path, even if many other aspects remain flexible, while "**Obs**" always denotes some observation of a system that is not a simulation. The following list of Brief and Explicit Names is given to reduce confusion. In addition, we offer a short explanation that will be used to create a Summarizing name at a later point.

This list is by no means complete. As long realized in biomedical research, there are numerous benefits to using a system that can combine terms and fragments to derive new composed terms denoting new meaning (often based on Latin or Greek roots [7]). POST will not need all conceivable terms (which gives away the advantage of simplicity); indeed, a case can be made for introducing discipline/application/industry specific vocabularies, which should be defined in combination with the specification of a controlled type for the PHF. Ultimately any such efforts require the construction of an ontology [8, 9] or type system to keep POST well defined.

4.2.1. Top Level Key words in Brief Dictionary

Other Keywords with reserved top folders and independent subfolder namespaces:

- **Def** Definition,
- **Est** Estimate,
- **Obs** Observation,
- **Sim** Simulation,
- **Res** Result,
- **Lab** LabAutomation,
- **Log** LogAutomation,
- **WebS** WebSubscribed,
- **WebP** WebPublished,
- **MO** Mode_of_Computing,
- **FSM** FiniteStateMachine,
- **Ma** Machine,
- **Me** Memory,
- **Sp** Space,
- **Fu** Function,
- **Te** Template,
- **Ro** Role,
- **QAS** QualityAssessmentSummary (Human),
- **Pbc** ProblemTreeCompilation (Automated),
- **Data** Data (Unspecified),
- **Type** Type (Unspecified),
- **Code** Code (Unspecified),
- **Idea** Idea (Unspecified),
- **Opi** Opinion

4.2.2. Extensions of Top Level Key words in Brief Dictionary

Combine the given 'NameStarter' from above with any of its reserved follow-on 'KeyNameFragment' below (or use subfolders; defining the full intended meaning in Def/POST):

- **Preprocessed (Obs-)**:
  - **Adj** Adjusted,
  - **Chk** CheckedIntegrity,
  - **Maj** Majority,
  - **Mis** Mistake,
  - **Mod** Modified,
  - **Odd** Outlier,
  - **Raw** AsReceived,
  - **Sca** Scaled,
  - **Tra** Transformed,
  - **Try** Risk;

- **Estimation (Est-)**:
  - **Gue** Guessed,
  - **Pre** Predicted (=Gue*Lkh),
  - **Evi** Evidence,
  - **Upd** Updated,
  - **Lkh** Likelihood,
  - **Xpl** Explained,
  - **Fct** Forecast,
  - **Dst** Distance,
  - **Add** Addition
  - **Mul** Multiplication
  - **Dif** Difference,
  - **Div** Division
  - **Difl** Differential,
  - **Cal** CalculatedAnalytic,
  - **Sim** SimulatedComputationally,
  - **EIndx** EstimatedResultsIndex;

- **SimulatedModelResult (Sim-):**
  - **MFr** ModelFrame,
  - **MSt** ModelStructure,
  - **MVa** ModelVariant,
  - **MRe** ModelRepeat,
  - **MIndx** ModelResultsIndex;

- **ProblemTreeCompilation** **(PBC-)**:
  - **Err** ErrorTree,
  - **Wrn** WarningTree,
  - **Rpt** ReportTree,
  - **Ifo** InfoBlockTree,
  - **Why** WhyCausalTree,
  - **Slo** SlowProgressTree
  - **Dbg** DebugTree,
  - **Ast** AbstractSyntaxTree;

  PBC is to be expanded substantially. All these entries are compiled automatically (in contrast to Pbh entries below, which are entirely based on human annotation).

- **QualityAssessmentSummary** **(QAS-)**:
  - **Pbh** ProblemTree (Human annotations),
  - **Lim** LimitationsTree,
  - **Cal** CalendarTree,
  - **Mat** MaturityTree,
  - **Exi** ExistenceTree,
  - **QAT** QualityAssessmentTransformation;

  QAS is to be expanded substantially to facilitate a wide range of human annotations using controlled vocabulary for quickly annotating repeating quality assessment scenarios, including diverse fuzzy situations, where it is difficult to assess quality because of many unknowns.

4.2.3. Flexible Modifiers of other keywords in the Brief Dictionary

These modifiers cannot stand alone, but otherwise work very well in combination with some of the keyword fragments defined above:

- **Compared**:
    - **U** Usual,
    - **Q** Other

    \-
    - **Wt** Wildtype,
    - **Mt** Mutant,
    - **Wtl** Wildtypelike,
    - **Mtl** Mutantlike,

    \-
    - **Ni** Noise,
    - **Si** Signal

    \-
    - **Tst** TestExperiment,
    - **Ctr** ControlExperiment;

- **Seq**uence**Chain**:
    - **Frst** First,
    - **Prev** Previous,
    - **Curr** Current,
    - **Next** Next,
    - **Last** Last,
    - **CIndx** ChainElementIndex;

- **Relevance**:
    - **Core** KeyResults,
    - **Keep** InArchives,
    - **Late** LatestFewResults,
    - **Logg** LoggingAllResults,
    - **Fail** FailedTriedRisk,
    - **Bugs** BugsAnyReport
    - **Prob** ProblemsInModel,
    - **Reso** ResourceWarning,
    - **Temp** TemporaryIntermediateResults;

4.3. Defining *TrustedTested* in POST

The unusual nature and long-term importance of *TrustedTested* as a *StabilityLevel* merit a separate list of requirements and comments to explain how *TT* works.

Projects aiming to reach *TT* are encouraged to ask questions about how to enable long-term stability as early as possible and are cautioned against elevating code too quickly to *SS* without evidence that its design has what it takes to go all the way to *TT*; thus *SS* can be used as a testing ground that buffers *TT* from integrating solutions that have not received sufficient review. There is no need to risk elevating new features too quickly to *TT*. Many successful software products have demonstrated that remaining at the equivalent of *RR* does not hamper success, and many high-quality IT standards that provide stability at *SS* do so without the need for *TT* promises. *TT* stability is very difficult but not impossible to achieve, as IT systems change continually and *TT* systems need to anticipate and abstract these changes well enough to isolate them from affecting any code written for such *TT* systems. Thus, in the absence of evidence for extraordinary stability or if in any doubt, software projects providing a single high-quality implementation of their design are recommended to stay at *RR*, and official standards without guarantees for long-term backwards compatibility at *SS*. However, a precise definition of the POST requirements for transitioning from *QQ* to *RR* to *SS* and to *TT* is beyond the scope of this study and remains to be reported elsewhere.

a. **Simplify use for outsiders.** Any downloadable system developed using POST and marked "*TTv1*" indicates that it belongs to the version variant family
   "*TrustedTested version 1*" = "*TTv1*"   expect long-term stability
   which follows a well-defined set of requirements providing the following capabilities (where some details may need to be defined by the corresponding POST project):

b. **Work with all older code written for stability.** The latest downloadable release or patch of a *TT* version variant family can correctly interpret any code produced for any *previous* release or patch of this *TT* version variant family (e.g. *TT* version 1 can interpret any code for any release or patch back to v1r0p0, the original *version* 1 *release* 0 *patch* 0)

c. **Use Stabilizing Versioning.** It is beyond the scope of this study to describe the stabilizing version variant naming system implied here; once fully defined, it is to become part of POST.

d. **Avoid breaking backwards compatibility.** In the POST system standard, breaking the compatibility of a new *TTv1* release with any previous variant of *TTv1* is not allowed and requires the definition and implementation of *TT* version 2 with an automated translator that correctly produces *TTv2* code from any previous *TTv1* code that is no longer compatible. Thus the release of any new *TT* version essentially terminates the series of releases for the previous *TTv* and triggers the need to automate the migration of a potentially very large code-base (making this a very costly operation not to be undergone lightly if unavoidable at all).

e. **Clearly mark code that is not (yet) stable** either as *TTv0* or by omitting *TT* or by adding other *StabilityLevels*:

"*TTv0*"	do not yet expect **long**-term stability
"*SSv1*"	do not expect **long**-term stability
"*RRv1*"	do not expect **medium**-term stability
"*QQv1*"	do not expect **short**-term stability
…	expect less and lesser **short**-term stability
"*MMv1*"	expect least **short**-term stability

Given the many strict requirements and prolonged review processes required for developing code at the *TT StabilityLevel*, projects aiming for *TT* first need to denote many version variants that do not yet meet *TT* criteria. Code that does not contain any *TT* level code is to be annotated at the appropriate level (*MM…SS*). Code modifying any pure *TTv1* variant with less reliable changes is to be denoted by adding the *Brief Names* of its lower *StabilityLevels* to the version variant label, thereby indicating the loss of *TT* stability. For example, in

"*TTv1r2* "	has only features of *TT* version 1 release 2
"*TTv1r2_OOv3r4*"	*TT* core (v1r2) with *OO* extensions v3r4
"*TTv1r2_OOv3r4_MMv0r0p3*"	further extended by a hack at *MM* level,

the overall stability is specified by the lowest *StabilityLevel* present, even if parts of the system are more stable since they were not modified. However, the presence of any modifications makes it difficult to exclude interactions that destabilize the whole system. Hence all modifications of any code above *TTv1r0p0* require full review in the context of how they affect the whole system they have been added to.

f. **Minimize contradictions**. All output from *TT* systems is required to be accurate to the best of current knowledge, which may advance as research progresses. This implies the emergence of new algorithms and/or fixes for bugs in known algorithms. Bug-fixing *TT* patches can in principle change output if the older system produced output that is demonstrably wrong. However, appropriate review at other stability levels is expected to catch such bugs before an algorithm is advanced to *TT*. In areas that are known to be difficult to standardize, such as numbers and arithmetic systems, alternative systems can be distinguished by corresponding differences in names, thus enabling the addition of new interpretation systems without breaking the compatibility of code that rely on previously implemented systems. Similarly, the discovery of improved algorithms does not require abandoning the possibility of using previously used algorithms. However, new algorithms shall eventually result in *TT* releases that automate running new algorithms in addition to old ones, facilitating comparisons of results. Design and algorithm choices that are *TrustedTested* are expected to have survived multiple rounds of rigorous conceptual and usability review, repeated design simplifications, prolonged use in professional production environments, and many automated test cases. A full list of criteria for justifying the progression of code to *TT* is beyond this study, except to say that it should not happen too fast to minimize contradictions and to simplify design (see next).

g. **Use rigorous review for reducing clutter in namespaces**. Careless design decisions can quickly and irrevocably clutter the *TTv1* namespace of a given project and thereby increase its inessential complexity[10]. This can quickly degrade the prospects of long-term survival for the project. The special nature of *TTv1* features causes this, features that signal to users and

programmers that they will all remain available on the long term. Thus the leadership of POST based projects is recommended to be slow and careful when allowing names to enter into the *TTv1* namespace. The POST *StabilizingZone* does not require such rigor for *TTv0* or any full versions of other stability levels (*MM* … *SS*), providing many opportunities for experimenting with mutually incompatible competing implementations of a new feature before selecting one of them for *TTv1*. While POST generally expects an increase of stability from *MM* to *SS* and with increased version numbers within a *StabilityLevel*, it expects even more that systems become public at *RR* and noticeably reduce the changes that remain possible as stability moves through the levels *RR* -> *SS* -> *TT*, while incorporating worthwhile improvements suggested in a *FeedbackFlow* from public users.

The path to TrustedTested: an overview of the *StabilizingZone*.

Here *GG* serves as the “Null-Element”, that moves equivalent content outside of the *StabilizingZone*, if there is no aim to ever attain long-term backwards compatibility. There is no difference in the assurance of stability between GG and MM, only a difference in ultimate intention.

VV serves as the installation location and ultimate archive for any products produced by the *StabilizingZone*.

XX provides temporary build space needed for generating the final installation-ready files (its stability will be copied from independent upstream code that produces it, hence XenoXero).

**GG**	GrandGallery	GrandGallery_UsedFor_Presenting_BestExhibits_LatestResults__ __FinalKeyDeliverables_without_NeedForLongTermStability
**MM**	MockupModel	MockupModel_UsedFor_RapidPrototyping_InformalLearning__ __ExpertimentsToBeThrownAway_StabilizingDesignNotCode
**NN**	NewNonfunctional	NewNonfunctional_UsedFor_NotYetFunctioning_DeepFoundations__ __ForLargerStableDesigns_ThatDoNotYetWorkForUsers
**OO**	OperatesOften	OperatesOften_UsedFor_Systems_PartiallyWorkingForEndUsers__ __while_StillMissing_ImportantFeatures_ToBeImplemented
**PP**	PreProbing	PreProbing_UsedFor_Preparing_PeerReviewAndPublicProbing__ __by_PolishingExistingFeatures_UntilSubmissionFor_Questioning
**QQ**	QualityQuest	QualityQuest_UsedFor_Questioning_AxiomsDataScienceAccuracy__ __RigorClarityUsability_InMany_ExpertBeginnerReviewRounds
**RR**	ReviewedRelease	ReviewedRelease_UsedFor_NewReleasesRecommended_by__ __QualityQuestEditors_after_AnsweringAllReviewerQuestions
**SS**	StableSource	StableSource_UsedFor_StunningSoftware_RunningInProduction__ __with_LongTermSuccess_and_VeryRareRevisionRequests
**TT**	TrustedTested	TrustedTested_UsedFor_Marking_VeryLongTermStableDesigns_in__ __WellUnderstoodDomains_AllowingBackwardsCompatibleGrowth
**VV**	VersionVariant	VersionVariant_UsedFor_InstallingAllLocalProducts_FullyArchived__ __Checksummed_ReproduciblyWorking_AllFiles_Ready2Publish

5. Design

The following information is advanced and meant for software architects, designers, and developers; it is not intended for a general audience.

5.1. Functional requirements and features

The POST system was optimized in a trade-off between the following requirements:

a. **Easy entry point** for newcomers with as few required structures as possible (none).

b. **Simple growth** by adding only structures that are needed locally (one by one).

c. **Conceptual clarity**, providing roles for each structure that are well defined and of the highest importance for corresponding large projects.

d. **Brief memorable keywords**, including the use of all double capital letters of the English alphabet as *Brief Names* for *StabilityCodes* with matching *Explicit Names* carefully chosen as memorable reminders of their meaning (example: *HH* for "*HistoryHeap*").

e. **Memorable organization** of *StabilityCode* names along the alphabet roughly tracking stages of project progression (start with early letters for early stages; minimize exceptions).

f. **Use of the alphabet** to group *StabilityCodes* by types for well-defined use cases (choosing the clearest *Explicit Names* with corresponding initials to sort accordingly with the alphabet).

g. **Flexible** to allow extremely complex or simple project structures to exist concurrently.

h. **Clear transitions and support for both manual and automated operation** of POST in many diverse storage structures (folders in file systems, Git repositories, sets with nested subsets and elements, arbitrary data structures, diverse off-line paper-based systems, etc.)

i. **No upper limits for nesting** full-scale POST systems within other POST systems unless explicitly named (such as absolute file path length limits; storage size limits, etc.).

j. **Clearly defined rules for making it easy to create tools for automating** tedious tasks (such as activating or versioning the content of folders with various levels of stability).

k. **Support for all key aspects of system development** from first idea over the various stages of the software development life cycle to archiving the last results.

l. **Defined information flows** between various containers with different *StabilityCodes* (specifying which flows are allowed and which are not).

m. **Utmost reduction of inessential complexity to maximize usability**, but without sacrificing scalability or other functions that are essential for helping to organize and stabilize projects

(together with alphabetical and mnemonic requirements this resulted in considerable naming challenges, especially for identifying memorable *Explicit Names*; we cannot see how to meet these challenges without at least separating *Brief* and *Explicit Names*; much of the value of POST is defined by the quality of this integration task).

n. ***Summarizing Names*** for *StabilityCodes* that serve as "mini documentation", reduce the need to consult a manual, and provide a nucleus for standardizing POST system semantics.

o. **Define a *StabilizingZone* with defined *StabilityLevels*** to serve as anchor for a stabilizing version variant numbering system that can help software projects to steer code towards long-term backwards compatibility by explicitly disentangling the speed of fast 'agile' prototype development (such as *MockupModel, MM*), from slow highly organized 'waterfall' project development (typically published as *ReviewedRelease, RR*), and in turn from the glacial pace of development of most international standards (usually *StableSource, SS*, albeit without excluding future changes or promising migration capabilities); keep these use cases separate from the endpoint of the *StabilizingZone* (*TrustedTested*, *TT*), which is required to remain 'stable forever' to the best of current knowledge or provide a migration path (see below).

p. **Encourage long-term backwards compatibility** of systems developed with POST by helping end users to quickly recognize version variants offering such maximal stability by providing a clear and memorable label (*TrustedTested*, *TT*) linked to a rigorous review process.

These requirements must be included in future extensions of the POST system that ideally extend and refine this list in order to move POST closer to the stability denoted as *TrustedTested*.
The POST definitions given here are not yet at *StabilityLevel TT*, but were designed with the aim to encourage and measure progress towards developing *TT* stable code in and for Evolvix (in order to reduce the cost of irreproducible research). This implies that the POST system defined here (and in particular its *StabilizingZone*) is closer to *TT* than any other code developed in or for Evolvix (see statement on status in section below). Thus, good reasons need to be provided for either

- **removing** any requirement specified above to justify why the corresponding POST use cases are best expressed in other ways, or
- **adding** requirements to justify why the corresponding use cases are important enough to merit inclusion in POST more than they merit removal to serve the aim of reducing the cognitive complexity for all users of POST (since beginners and experts pay for clutter caused by inessential complexity in POST, albeit in different ways).

5.2. Rules for automating and extending POST Home Folders

To be automatable, a PHF such as ‘*MyProjectFolder’* must adopt these structures and rules:

**File system path to folder**	***!! Comment explaining the folder on this line***
MyProjectFolder	*!! The POST Home Folder is the anchor link base for automation.*
…/_POST	*!! POST_Cabinet holds folders for all StabilityCodes as needed.*
…/_POST/AHA	*!! All folder names beginning/ending with Brief Name*
	!! StabilityCodes are reserved in here*, and populated as needed.*
*…/_POST/ZZ**	*!! Reserve names starting with “ZZ*” in all POST_Cabinets; ZZ is*
	!! always outside; other ZZ folders may be inside or outside.*
*…/_POST/**	*!! To enable the development of a controlled POST vocabulary,*
	!! reserve all remaining names in all POST_Cabinets for future
	!! use (see Section 4.2 for examples); the names can be
	!! “borrowed”, as long as it is understood, that POST may claim
	!! any name inside _POST at any time. When that happens, the
	!! PHF will be equipped with a well-defined type that specifies all
	!! names that have been reserved (and the remaining liberties).
…/…	*!! Active area of the PHF, that can be replaced by content of*
	!! another StabilityLevel or VersionVariant.
*…/*ZZ	*!! Content stored here is ‘inside’, but not managed by this PHF;*
	!! ZZ can contain anything, including other PHFs; ZZ is never
	!! replaced with content of other StabilityLevels or VVs.
*…/*ZZ*	*!! Reserve names starting with “ZZ*” (+lower case) in all PHFs.*
*…/*ZZA	*!! Anchors: declared links to folders or files outside of PHF;*
	!! any file/folder/web/etc link referenced in this PHF that is not
	!! inside and controlled by this PHF must go through ZZA.

Naming rules for files/ folders or elements/sets aiming to avoid name clashes with PHF POST infrastructure shall exclude these reserved POST names (in *all* upper and lower case variants):

a. Core PHF names: ‘_POST’, ‘ZZ’, and each name starting with ‘ZZ’ (without separators)

b. Core names inside of _POST: each name that is, starts or ends with any Brief or Explicit Name of any *StabilityCode* in Figures P2 and P3; and ‘WEB’, ‘POST’, ‘BOX’, ‘NET’, or any combination of the last three; where ‘_’, or ‘.’, or ‘-‘ can lead, or trail, or separate these labels from the remainder of the name (or be the name) in any combination; also, each ZZ* name.

c. When developing scripts for a PHF, it is currently recommended to also avoid in the PHF namespace all core names explicitly defined for the inside of ‘_POST’ (i.e. the POST_Cabinet), or to use them there as defined by POST.

d. Rules for names inside of automated top-level POST_Cabinet folders (e.g. ‘AAA’,‘HH’, etc) have not yet been specified and will restrict some of their namespace while automation of the corresponding functionality is switched on (but not during manual operation).

Restrictions are to be reviewed to reduce them to the minimum necessary to support all key functions, which shall include internationalization and adaptations to allow in principle simultaneous work with diverse compilers, clouds and operating systems (as far as technically possible). Developers of scripts for PHFs are recommended to be cautious when making assumptions about POST names inside of top-level folders (and consider contacting the architect of POST). This caution can be ignored for the folders specified below and for purely manual POSTboxes.

e. All content of the following top-level POST_Cabinet folders shall be guaranteed to remain completely free from any restrictions or required additions. When future POST standards facilitate their automated management, corresponding metadata on content of these free-form folders must be stored elsewhere in their local PHF to maximize free content choice by users as much as relevant clouds and operating systems allow. The FreeFormFolders are:

   II, *GG*, *MM*, *NN*, *OO*, *PP*, *QQ*, *RR*, *SS*, *TT*, and *ZZ*.

   Note that users can turn any of these folders into PHFs or add PHFs of some type; this may make it necessary to distinguish the top PHF's '_POST' and 'ZZ*' folders from those of lower-level folders; a corresponding re-naming-when-active shall be developed as needed.

5.3. Translation in POST folders

Texts that need any sort of translation are generally stored as programmatically accessible stable links in the *AHA* folder which immediately splits into relevant languages (using ISO language codes). While this facilitates the translation of all user documentation and potentially important texts within applications, it does little for translating the POST folder itself, which remains overwhelmingly "English" or rather "abbreviated English". To customize its appearance for English and/or international users, we recommend to create links, similar to the approach of the link-farm manager "Stow" [11].

6. POST specification: current status, stability and future work

The POST specification details in this document define RRv1r0p0, or, omitting zeros, RRv1, both of which are Brief Names for this Explicit Name: *ReviewedRelease* version 1 release 0 patch 0.

Influences that shaped RRv1 include

(i) analyzing key requirements for the efficient management and development of complex software projects as observed by the main architect of POST over 15+ years in professional computational biology research environments;

(ii) surviving intense questioning in multiple rounds of review for more than a year, focusing on the aim to minimize inessential complexity without sacrificing essential functionality;

(iii) integrating a substantial and detailed (albeit still informal) feedback flow from diverse usability and expert reviewers, including practical experiences made by several authors of this study while using manual *POSTboxes* in clouds on different operating systems;

(iv) initial work gathering requirements and implementing file system folder structures in Evolvix with the goal to make them more uniform and easier to manage.

Completeness in all areas is not provided by these efforts as indicated by the reserved names:

(i) Important details about *POSTnet* automation and related substructures of the folders associated with *StabilityCodes* remain to be specified.

(ii) POST reserves *all* folder and file names starting with 'ZZ' for future extensions (briefly summarized as 'ZZ*'), both in the PHF and in its '_POST' folder to meet diverse needs in areas known to be important, but not yet addressed by POST so far (including orderly storage of simulation results in file-systems, clearly defining folders for data import or export, and many more). All these ZZ* codes need to cover certain areas of computational and storage related semantics to facilitate efficient POST automation, including aspects related to swapping the active content of different stability levels, Git, Memory, Backup, renaming, metadata and many other data structures and operations.

(iii) There is a need to define all POST relevant version variant algebra sets and operations to implement the systems that automate storing, increasing and switching version variants.

(iv) As long as POST is at RRv1 any attempts to simplify without loss of expressivity are welcome. While *RR* establishes some interest to keep continuity, radical changes are still possible if a need is found to justify them. This liberty is shrinking fast on the path towards *TT* and will be completely gone once *TT* is reached. Therefore we recommend to simplify as much as possible now to reap the benefits later.

(v) Recommendations are needed for what to require to advance to the next level on the *StabilizingZone* for projects eager to do so. While any levels up to *QQ* are essentially to be managed internally by a project, POST demands that *RR* actually is a public 'release' that has been 'reviewed' by someone with a position that is authorized to do so within the project. However, *RR* does not require outside interactions and thus the quality of *RR* releases is expected to vary substantially among projects. Progress from *RR* to *SS* to *TT* requires increasingly stringent external evidence of substantial long-term stability in the face of repeated rounds of real in-depth review. Details remain to be specified, as well as how to document them automatically.

It is beyond the scope of this work to finalize POST or to discuss more semantic details of the *StabilityCodes* we named below. Our definition of POST is one of several examples from Evolvix development that we could have given to illustrate how the BEST Names concept might facilitate the navigation of complex areas and how it can affect the development of a programming language by enabling filled programming language design approach (which was extensively used to develop POST; this would have been impossible to achieve otherwise). POST is a particularly fitting example for the power of BEST Names, as conflicts in naming requirements for different reasonable use cases of POST *StabilityCodes* are likely to make POST-like systems either so terse or so verbose that beginners or power-users respectively, will find them almost unusable (unless an equivalent of the BEST Names concept is used to disentangle both legitimate needs). We certainly would not have developed POST without the possibility to implement a BEST Names infrastructure.

We used POST here as an example for BEST Names. However, this does not diminish the importance of the POST specification published in this particular publication, which is rated at RRv1 by its architect and current guardian. The BEST Names given in Figures P2-P3 thus mark an important milestone of POST design, which now provides a substantial organizational infrastructure that integrates a broad spectrum of key areas required for managing projects small or large. It has reached a substantial level of refinement that has been deemed enough to merit implementation in the core of Evolvix that aims for long-term stability.

The *StabilizingZone* from *MM* to *TT* is of paramount importance for core Evolvix as it is essential for achieving the goal of reliable long-term backwards compatibility by disentangling the faster development of experimental code innovations from the much slower pace that characterizes the development of robust long-term standards (which highly benefit from many rounds of review and is therefore slow). Given this essential role, it is important to collect experiences with this approach to develop backwards compatible code. Accordingly, great care was taken to structure the POST system presented here in a way that allows its formal features like *StabilityCodes* to remain unchanged as POST is expanding to meet new needs. This allows older code to remain stable as the remaining features are added to POST without removing existing functionality. The internal goal in POST development has been to propose a syntax and semantics that stands a chance to be of *TT* quality, even though it is currently ‘only’ released as *RRv1* to reserve the possibility to change core aspects of the design in case a new essential need is to be met.

Justification of *RRv1*: Many aspects of POST are much more stable than *RR* (e.g. the concept of a *StabilizingZone* itself and the “double caps keyword” idea have not changed in a long time despite much turnover and no lack of attempts to improve, therefore these are possibly close to or at “*TT*”). However, other POST aspects have not been explored and tested enough to warrant an overall label beyond *RR* for the system as a whole (which in POST is limited by the stability of its weakest elements). The precise nature of the hurdles that need to be overcome by any idea to reach “*SS*” or “*TT*” is an open area of development in POST and beyond the scope of this paper even though it can ultimately be reduced to a naming problem.

RR with the aim to reach *TT* implies the strongest possible invitation to anybody anywhere to join the Quality Quest to scrutinize, test, improve and polish any such proposed standards as much and as soon as possible in all conceivable aspects to improve the chances of long-term stability, usability and usefulness. To ensure an appropriately balanced integration of all received feedback that remains as simple as possible in the context of its bigger system, the POST system requires the existence of one

single guardian brain equally dedicated to protect and expand the simplicity, usability, expressivity, accuracy, and security of a *TT* grade standard that can serve users internationally on the long term. While this guardian is likely to critically depend on external input that may come from a supporting committee that helps to represent many voices and perspectives appropriately, such committees usually do not easily arrive at solutions as elegant and effective as capable guardians that are dedicated to finding the best possible solution to serve the community on the long-term.

Feedback submission: Until more efficient ways of integrating feedback are implemented, please contact the architect of POST (L. Loewe) with any general improvements and in particular with comments on the *StabilizingZone*. To reach long-term stability as fast as possible, an important aim of current POST development is to get the *StabilizingZone* itself as fast as possible to *TT* level stability – albeit without allowing weaknesses to remain in the definitions of names from *MM* to *TT*. Such weaknesses could easily become disastrous for code relying on *TT* stability that would then no longer be able to rely on stable names for labeling stability.

Please consult the POST home on the web at

http://evolvix.org/post

for updates or further details on how to contribute to POST development.

7. References for POST

1. Darwin, C. 1859. *On the origin of species by means of natural selection*. J. Murray. London,.
2. Loewe, L. 2007. Evolution@home: observations on participant choice, work unit variation and low-effort global computing. *Softw Pract Exper*. **37**: 1289-1318.
3. Hucka, M., A. Finney, H.M. Sauro*, et al.* 2003. The systems biology markup language (SBML): a medium for representation and exchange of biochemical network models. *Bioinformatics*. **19**: 524-531.
4. Auffray, C. & L. Hood. 2012. Editorial: Systems biology and personalized medicine - the future is now. *Biotechnol J*. **7**: 938-939.
5. Loewe, L. 2016. "Systems in Evolutionary System Biology". In *Encyclopedia of Evolutionary Biology*, Vol. 4. Richard M. Kliman (Chief) & Hiroshi Akashi (Section), Eds.: pp. 297-318, Oxford: Academic Press (Elsevier). (http://evolutionarysystemsbiology.org/pdf/Loewe-2016-evosysbio.pdf).
6. Ehlert, K. & L. Loewe. 2014. Lazy Updating of hubs can enable more realistic models by speeding up stochastic simulations. *J Chem Phys*. **141**: 204109.
7. McKeown, J.C. & J.M. Smith. 2016. *The Hippocrates code : unraveling the ancient mysteries of modern medical terminology*. Hackett Publishishing Company, Inc. Indianapolis, Indiana.
8. Arp, R., B. Smith & A.D. Spear. 2015. *Building ontologies with Basic Formal Ontology*. Massachusetts Institute of Technology. Cambridge, Massachusetts http://ontology.buffalo.edu/smith/.
9. van Renssen, A.S.H.P. 2005. *Gellish: A Generic Extensible Ontological Language - Design and Application of a Universal Data Structure*. Delft University Press. Delft, Netherlands. http://resolver.tudelft.nl/uuid:de26132b-6f03-41b9-b882-c74b7e34a07d.
10. Raymond, E.S. 2003. *The Art of Unix Programming*. Addison-Wesley (Pearson). Boston, MA, USA. http://www.catb.org/~esr/writings/taoup/.
11. Glickstein, B. & K. Hodgson. 2015-11-09. GNU Stow 2.2.2, a symlink farm manager. https://www.gnu.org/software/stow/.

InfoFlows in a Project Organization Stabilizing Tool (POST) Home Folder (PHF)

POST aims to reduce organizational burdens in info-processing projects of any size by associating frequently used types of meaning with standardized Brief and Explicit *'StabilityCode'* Names chosen to be memorable. You choose the *StabilityCodes* your project needs from the general scaffolding, organizing and stabilizing blueprint given by POST; it does not matter whether you are writing a small text or a big complex nested software system that needs long-term backwards compatibility. **Arrows below** show frequent InfoFlows between *StabilityCodes.* See *Brief Dictionary of* POST and *Intro* for more details.

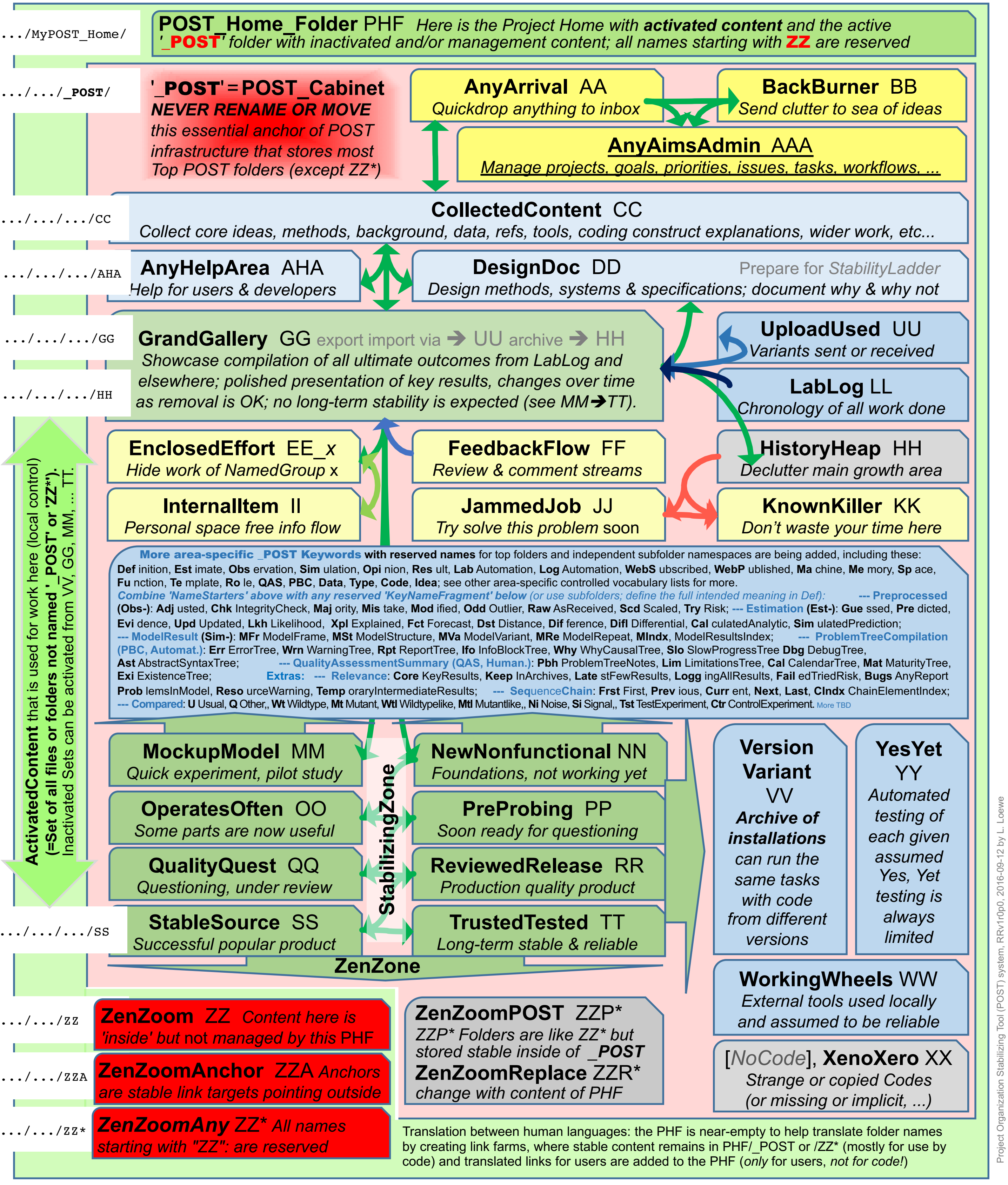

Brief Dictionary of the Project Organization Stabilizing Tool (POST) system

Synonymous **Brief**, **Explicit**, and **Summarizing** Names of POST *StabilityCodes* define keywords in Evolvix for fast data annotation

Use Case	Type:POST	Brief	Explicit Name	Summarizing Name of *POSTcode* or *StabilityCode*	Mainly used by ...
**Getting organized**	AnyZone	**AA**	AnyArrival _WorkspaceID	AnyArrival_UsedFor_Inbox_QuickDropping_Items_to_ProcessLater	Developer, Lead, Anybody
	Workflow Zone	**AAA**	AnyAimsAdmin _WorkspaceID = Name of Workspace (if needed)	AnyAimsAdmin_UsedFor_Managing_StrategyAims_ProjectPlans__ __Workflows_RoleDefs_Tracking_TasksDeadlinesFunds__ __RecruitingTraining_Delegation_CallBacks_Prioritizing_KeyGoals	Project **Leaders** (Lead) Expert **Developers** (Dev) Any Contributor
		**BB**	BackBurner _WorkspaceID	BackBurner_UsedFor_Storing_SeasOfGoodIdeas_to_RevisitLater	Dev, Lead
**Ordering collected info**	Info Zone	**AHA**	AnyHelpArea	AnyHelpArea_UsedFor_OnlineHelp_UserManuals_InfoMessages__ __TeachingOutreachMaterial_Translatable_by_CountryCode	New outside **Users** (User) Dev, Lead
		**CC**	CollectedContent _WorkspaceID	CollectedContent_UsedFor_Collecting_External_TopicIdeas_Data__ __Methods_Evidence_Refs_Reviews_Clarity_CodeExplanations	System **Designers** (Design) Lead, Dev
		**DD**	DesignDoc _WorkspaceID	DesignDoc_UsedFor_Describing_ImplementableTechSpecification__ __and_ProsCons_of_DesignDecisions_Methods_AlternativeIdeas	*Produced* by Design, Lead *Used* by Dev,...
**Restricting access for reviewing**	Review Zone	**EE**	EnclosedEffort _WorkspaceID	EnclosedEffort_UsedFor_Collaborating_InClosedGroups__ __with_AccessRestricted_as_AgreedBy_TheNamedGroup	Anybody from named group
		**FF**	FeedbackFlow _WorkspaceID	FeedbackFlow_UsedFor_Logging_AnyInputCommentCritiqueBug__ __Ideas_from_ReviewerExpertUserFriendFoeFans_Worldwide	*Produced* by User, Dev, Lead *Analyzed* by Design, Dev, ...
**Keeping instant startups simple**	Instant StartUp Zone	**GG**	GrandGallery _WorkspaceID	GrandGallery_UsedFor_Presenting_BestExhibits_LatestResults__ __FinalKeyDeliverables_without_NeedForLongTermStability	Anybody as agreed
		**HH**	HistoryHeap _WorkspaceID	HistoryHeap_UsedFor_OldVersionBackup_ProbablyDeletableFiles__ __HoardedDataWithLimitedOrder_KeptFor_PotentialUsefulness	Anybody as agreed
**Managing problems Try things**	Scratch Zone	**II**	InternalItem _WorkspaceID	InternalItem_UsedFor_ScratchPad_AnyChaoticContent_MeantFor__ __YourCreativeMuse_IsReadableUnlessYou_RestrictAccess	Personal (my space has no order anybody can understand)
		**JJ**	JammedJob _WorkspaceID	JammedJob_UsedFor_Documenting_BugReports_DecisionNeeds__ __and_ProgressOn_SolvableProblems _that_NeedSolutionsSoon	Dev, Lead, User, Design, ...
		**KK**	KnownKiller _WorkspaceID	KnownKiller_UsedFor_Documenting_DeprecatedCode_FaultyData__ __FailedIdeas_BadSolutions_etc_ThatKillTimeWhenRevisited	Dev, Design, Lead, Review, ...
		**LL**	LabLog _WorkspaceID	LabLog_UsedFor_Logging_AnyLabLabor_Dated_YYYY_MM_DD__ __ForHistoricSequenceOf_AllOutcomesOf_AllTests_IncludingFails	Dev, Design, Lead, Review, ...

More area specific keywords with reserved top folders and independent subfolder namespaces are being added, including the following: **Def** inition, **Est** imate, **Obs** ervation, **Sim** ulation, **Opi** nion, **Res** ult, **Lab** Automation, **Log** Automation, **WebS** ubscribed, **WebP** ublished, **Ma** chine, **Me** mory, **Sp** ace, **Fu** nction, **Te** mplate, **Ro** le, **Data**, **Type**, **Code**, **Idea**; see other area-specific controlled vocabulary lists.

Use Case	Type:POST	Brief	Explicit Name	Summarizing Name of *POSTcode* or *StabilityCode*	Mainly used by ...
**Maximizing stability** for projects of **importance**, by climbing inside of the ***Stabilizing Zone*** and becoming ideally: **Stable Forever** Only fully long-term backwards compatible systems shall get to TTv1	Stabilizing Zone	**MM**	MockupModel	MockupModel_UsedFor_RapidPrototyping_InformalLearning__ __ExpertimentsToBeThrownAway_StabilizingDesignNotCode	Dev, Design, Lead, ...
		**NN**	NewNonfunctional	NewNonfunctional_UsedFor_NotYetFunctioning_DeepFoundations__ __ForLargerStableDesigns_ThatDoNotYetWorkForUsers	Dev, Design, Lead, ...
		**OO**	OperatesOften	OperatesOften_UsedFor_Systems_PartiallyWorkingForEndUsers__ __while_StillMissing_ImportantFeatures_ToBeImplemented	Dev, Design, Lead, ...
		**PP**	PreProbing	PreProbing_UsedFor_Preparing_PeerReviewAndPublicProbing__ __by_PolishingExistingFeatures_UntilSubmissionFor_Questioning	Dev, Design, Lead, ...
		**QQ**	QualityQuest	QualityQuest_UsedFor_Questioning_AxiomsDataScienceAccuracy__ __RigorClarityUsability_InMany_ExpertBeginnerReviewRounds	Outside expert and usability **Reviewers**, Dev, Lead
		**RR**	ReviewedRelease	ReviewedRelease_UsedFor_NewReleasesRecommended_by__ __QualityQuestEditors_after_AnsweringAllReviewerQuestions	User, Dev, ...
		**SS**	StableSource	StableSource_UsedFor_StunningSoftware_RunningInProduction__ __with_LongTermSuccess_and_VeryRareRevisionRequests	User, Dev (rarely), ...
		**TT**	TrustedTested	TrustedTested_UsedFor_Marking_VeryLongTermStableDesigns_in__ __WellUnderstoodDomains_AllowingBackwardsCompatibleGrowth	User only; development is over if review worked well, ...
**Keeping records repro-ducible**	Archival Zone	**UU**	UploadUsed _WorkspaceID	UploadUsed_UsedFor_Archiving_ExternalCommunicationLog_of__ __FullUploadsOf_InputTo_or_OutputFrom_GG_etc	Anybody as agreed (Log key imports & exports)
		**VV**	VersionVariant	VersionVariant_UsedFor_InstallingAllLocalProducts_FullyArchived__ __Checksummed_ReproduciblyWorking_AllFiles_Ready2Publish	All installations are easy to activate and use in situ
		**WW**	WorkingWheels _WorkspaceID	WorkingWheels_UsedFor_AllExternal_CodeProgramToolVersions__ __TrustedEnough_for_Integration_IntoThis_POSTHomeFolder	Dev, Design, Lead, ...
		**YY**	YesYet _WorkspaceID	YesYet_UsedFor_AutomatedTesting_of_EachUnitAssumingYes__ __and_EachFeatureOrWeaknessThatNeeds_YetToBeAutoTested	Dev, Design, Lead, ... most values are auto-produced tests
**Missing, conflicting, automated *StabilityCodes***	Flexible Zone	**XX**	XenoXero _WorkspaceID	XenoXero_UsedFor_StrangeAndCopiedCodes_DefinedElsewhere_ __AutoGenerated_Temporary_Unspecified_Unclear_or_BadCodes	Automatically, Dev, ...
**Managing links, limits, controls, and more.**	ZenZone	**ZZ**	ZenZoom	ZenZoom_UsedFor_Nesting_POST_ContentHierarchies_Requiring__ __StorageInsideOf_ButManagementIndependentFrom_ThisPOST	Automatically, ZZ contains independent POST Homes
		**ZZA**	ZenZoomAnchor	ZenZoomAnchor_UsedFor_Anchoring_StableLinkChainTargets__ __UsedInThisPOST_ToLinkToEasilyAdjustable_OutsideContent	Automatically, Dev, ...
		**ZZ***	Names starting with ZZ... are reserved	ZenZoom... All names starting with 'ZZ' are reserved for future POST automation use and must not be used in a POST Home Folder.	Automatically, Dev, ...
		**_POST**	POST_Cabinet	POST_Cabinet_UsedFor_StableAutomating_and_Decluttering__ __of_POSTHomeFolder_Stowing_AllNonActivatedPOSTContent	Automatically, Dev, ...

Project Organization Stabilizing Tool (POST) system, RRv1r0p0, 2016-09-12 by L. Loewe
